# Phenalenones from a Marine-Derived Fungus *Penicillium* sp.

**DOI:** 10.3390/md17030176

**Published:** 2019-03-18

**Authors:** Sung Chul Park, Elin Julianti, Sungjin Ahn, Donghwa Kim, Sang Kook Lee, Minsoo Noh, Dong-Chan Oh, Ki-Bong Oh, Jongheon Shin

**Affiliations:** 1 Natural Products Research Institute, College of Pharmacy, Seoul National University, San 56-1, Sillim, Gwanak, Seoul 151-742, Korea; sungchulpark@snu.ac.kr (S.C.P.); elin_julianti@fa.itb.ac.id (E.J.); sungjinahn@snu.ac.kr (S.A.); dkim0719@snu.ac.kr (D.K.); sklee61@snu.ac.kr (S.K.L.); minsoonoh@snu.ac.kr (M.N.); dongchanoh@snu.ac.kr (D.-C.O.); 2 School of Pharmacy, Bandung Institute of Technology, Jl. Ganesha 10, Bandung 40132, Indonesia; 3 Department of Agricultural Biotechnology, College of Agriculture and Life Science, Seoul National University, San 56-1, Sillim, Gwanak, Seoul 151-921, Korea

**Keywords:** herqueinones, phenalenones, *Penicillium* sp., marine-derived fungi, adipogenesis, anti-angiogenesis, anti-inflammatory

## Abstract

Six new phenalenone derivatives (**1**–**6**), along with five known compounds (**7**–**11**) of the herqueinone class, were isolated from a marine-derived fungus *Penicillium* sp. The absolute configurations of these compounds were assigned based on chemical modifications and their specific rotations. 4-Hydroxysclerodin (**6**) and an acetone adduct of a triketone (**7**) exhibited moderate anti-angiogenetic and anti-inflammatory activities, respectively, while *ent*-peniciherqueinone (**1**) and isoherqueinone (**9**) exhibited moderate abilities to induce adipogenesis without cytotoxicity.

## 1. Introduction

Fungi produce a wide variety of polyketide-derived metabolites. Among these, phenalenones are a class of hexa- or heptaketides bearing a perinaphtenone-type tricyclic system [1]. As summarized in a recent comprehensive review, these compounds can have immense structural variations, such as homo- and heterodimerization, the incorporation of additional carbon frameworks, and a high degree of oxygenation and nitrogenation as well as being complexed with metals [2]. A frequently occurring variation is the incorporation of an isoprene unit by forming either a linear ether or a trimethylhydrofuran moiety, and this variation is well-represented in the herqueinones from *Penicillium* sp. [3,4,5]. Fungi-derived phenalenone compounds have attracted significant interest due to their chemical structures, bioactivities, and biosynthesis [1]. With their diverse phylogenic origins, phenalenones are widely recognized as a representative group of fungal polyketides [1,2].

During the course of our search for novel compounds from marine-derived fungi, we reported the structures of herqueiazole and herqueioxazole, unusual pyrrole- and oxazole-containing phenalenones from a *Penicillium* sp. strain [6]. Herqueidiketal, a cytotoxic sortase A inhibitory congener, also possessed a novel skeleton containing a highly oxidized naphthoquinone moiety [6]. Despite its carbon skeleton being different from typical phenalenones, the presence of naphthalene and dihydrofuran moieties in herqueidiketal may further emphasize the wide structural variations of phenalenones. In our continuing search for such compounds, we isolated several structurally related phenalenones from a large-scale cultivation of this *Penicillium* sp. strain. Here, we report the isolation of eleven compounds (**1**–**11**) as well as the structure determination of six new compounds (**1**–**6**) in the herqueinone subclass. 4-Hydroxysclerodin (**6**) exhibited moderate anti-angiogenic activity on human umbilical vascular endothelial cells (HUVECs). The acetone adduct of a triketone (**7**) exhibited moderate anti-inflammatory activity in mouse macrophage RAW 264.7 cells. In addition, *ent*-peniciherqueinone (**1**) and isoherqueinone (**9**) moderately induced adipogenesis in human bone marrow-mesenchymal stem cells (hBM-MSCs). All of these bioactivities were found to occur without cytotoxicity.

## 2. Results and Discussion

The molecular formula of **1** was deduced to be C_20_H_20_O_8_ with 11 degrees of unsaturation by HRFABMS analysis. The ^13^C NMR data of this compound showed a signal of a ketone carbon at δ_C_ 197.6 (Table 1). The signals at δ_C_ 178.1 and 174.6 could belong to either carbonyl or highly deshielded olefinic carbons. The ^13^C NMR spectrum, in combination with DEPTs and HSQC spectra (Supplementary Figure S4), displayed nine nonprotonated sp^2^ carbon signals in the δ_C_ 103.5–162.8 region. The deshielded carbons must be one carbonyl carbon and one olefinic carbon, accounting therefore for seven degrees of unsaturation. The ^13^C NMR data also showed two oxygen-bearing quaternary sp^3^ carbons (δ_C_ 89.5 and 79.0), one methoxy carbon (δ_C_ 60.9), one shielded quaternary sp^3^ carbon (δ_C_ 46.9), and four shielded methyl carbons (δ_C_ 16.4, 16.4, 14.9, and 13.3) (Table 2). Combining the NMR data and the degrees of unsaturation, **1** must possess four rings featuring the herqueinone class of phenalenones.

**Figure 1:**
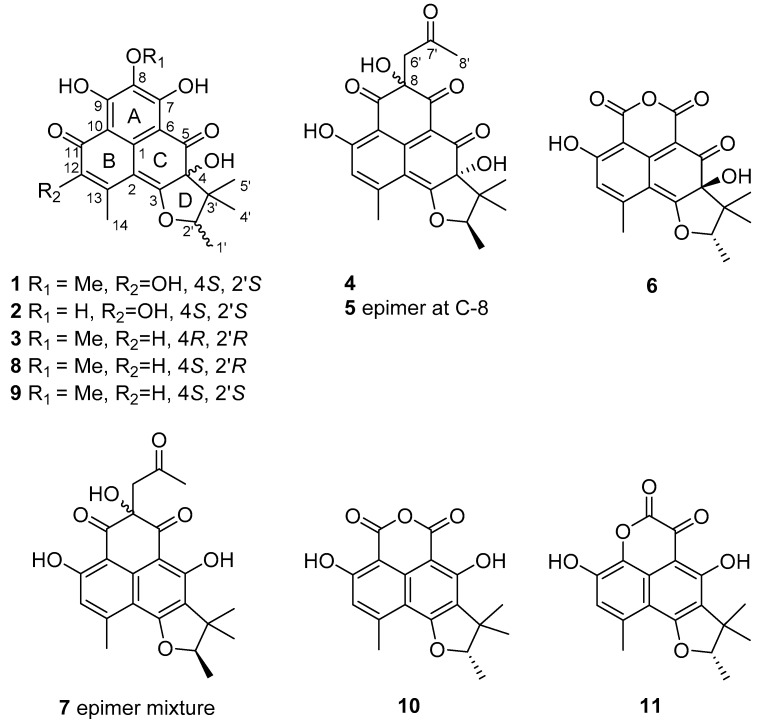
The structures of **1**–**11**.

Due to the lack of COSY correlations except for that from the methyl doublet at δ_H_ 1.40 (Me-1′) to the quartet at δ_H_ 4.99 (H-2′), the structure determination of **1** had to be carried out through extensive HMBC analyses under diverse measuring conditions (Figure 1). First, the long-range couplings from OH-7 (δ_H_ 13.23) to C-6 (δ_C_ 103.5), C-7 (δ_C_ 162.8), and C-8 (δ_C_ 131.6); from OCH_3_-8 (δ_H_ 3.92) to C-8 (δ_C_ 131.6); and OH-9 (δ_H_ 13.99) to C-8 (δ_C_ 131.6), C-9 (δ_C_ 161.7), and C-10 (δ_C_ 108.7) lead to a delineation of the C-6 to C-10 fragment. Aided by the four-bond couplings from OH-7 (δ_H_ 13.23) to C-1 (δ_C_ 137.3) and OH-9 (δ_H_ 13.99) to C-1 (δ_C_ 137.3) by decoupled HMBC (D-HMBC) [7] experiments, the presence of a hexa-substituted benzene ring (C-1, C-6–C-10; ring A) was confirmed. In addition, the combined HMBC and D-HMBC correlations from OH-12 (δ_H_ 6.66) and H_3_-14 (δ_H_ 2.47) to neighboring carbons revealed the presence of an *α*-hydroxy-*β*-methyl-*α*,*β*-unsaturated ketone group (OH-12 (δ_H_ 6.66) to C-11 (δ_C_ 178.1), C-12 (δ_C_ 143.7), and C-13 (δ_C_ 124.0); H_3_-14 (δ_H_ 2.47) to C-2 (δ_C_ 103.7), C-11 (δ_C_ 178.1), C-12 (δ_C_ 143.7), and C-13 (δ_C_ 124.0)), which was directly connected to the benzene ring based on the D-HMBC correlation from OH-7 (δ_H_ 13.23) to C-11 (δ_C_ 178.1).

In addition to the correlation from H_3_-14 (δ_H_ 2.47) to C-2 (δ_C_ 102.7), a correlation from H_3_-14 (δ_H_ 2.47) to a highly deshielded C-3 (δ_C_ 175.4) in the D-HMBC spectrum was crucial evidence for the attachment of an electron-withdrawing oxygen at this position. Subsequently, long-range correlations from OH-4 (δ_H_ 7.23) to C-3 (δ_C_ 8), C4 (δ_C_ 8), and C5 (δ_C_ 8) defined not only its connectivity to the C-2 double bond but also placed a carbonyl carbon (δ_C_ 198.2) at C-5. These carbon–proton correlations constructed an *α*,*β*-dioxycyclohexadienone moiety (C-1–C-6; ring C). The assignment of ring C also secured the formation of the conjugated carbonyl group to another six-membered ring (C-1, C-2, C-10–C-13; ring B).

**Figure 2:**
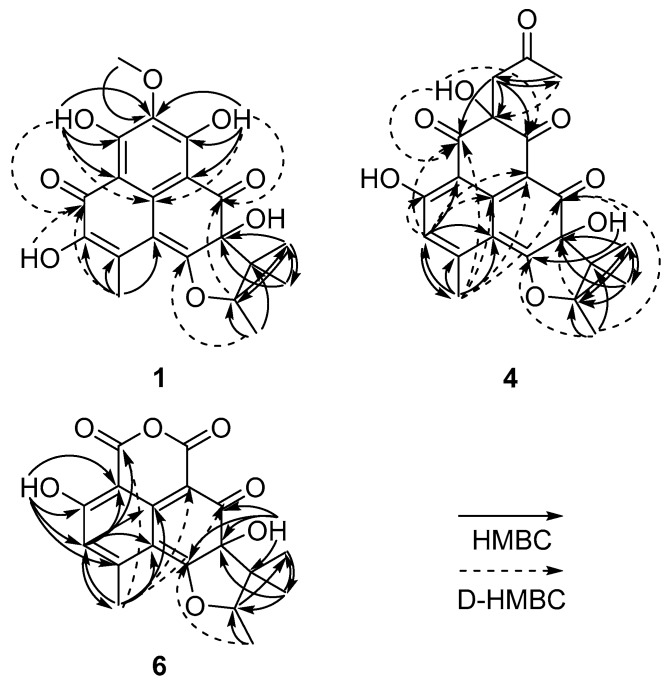
Key correlations of HMBC (arrows) and decoupled HMBC (D-HMBC) (dashed arrows) of **1**, **4**, and **6**.

The remaining C_5_ fragment (C-1′–C-5′) of **1** was readily defined as a 2,3-disubstituted 2-methylbutane moiety by a combination of COSY and HMBC data (Figure 2 and Supplementary Figures S3–S5). The cyclization of this moiety to the three-ring system was also accomplished by a series of long-range carbon–proton correlations. That is, the connection between C-4 and C-5′ was confirmed by the HMBC correlations from H_3_-4′ (δ_H_ 1.43) and H_3_-5′ (δ_H_ 0.86) to C-4 (δ_C_ 79.0) as well as a long-range correlation from H-2′ to C-5. The diagnostic chemical shifts of the CH-2′ methine group (δ_C_ 89.3, δ_H_ 4.91) suggested its attachment to C-3 via an ethereal bridge. This interpretation was corroborated by the correlation from H_3_-1′ (δ_H_ 1.40) to C-3 (δ_C_ 174.6), which established a hydrofuran moiety (C-3, C-4, C-2′, and C-4′; ring D). Thus, the structure of **1** was defined as a herqueinone-type tetracyclic phenalenone.

**Figure 3:**
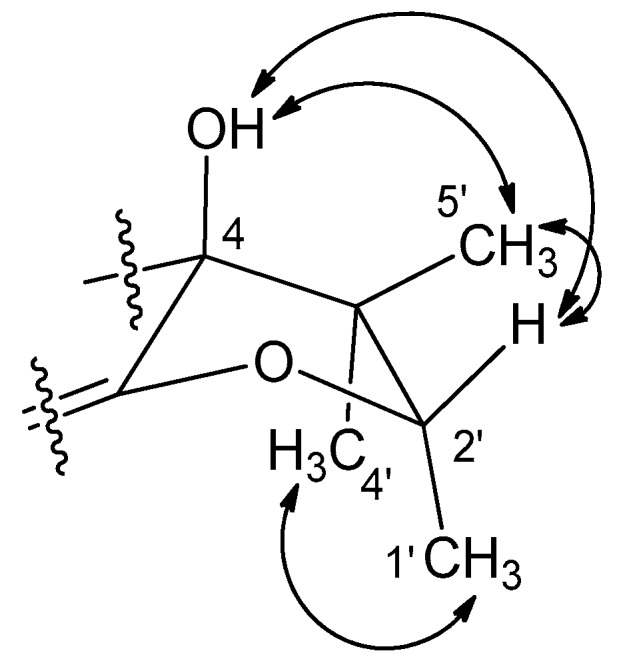
NOESY correlations of the hydrofuran moiety of **1**.

**Figure 4:**
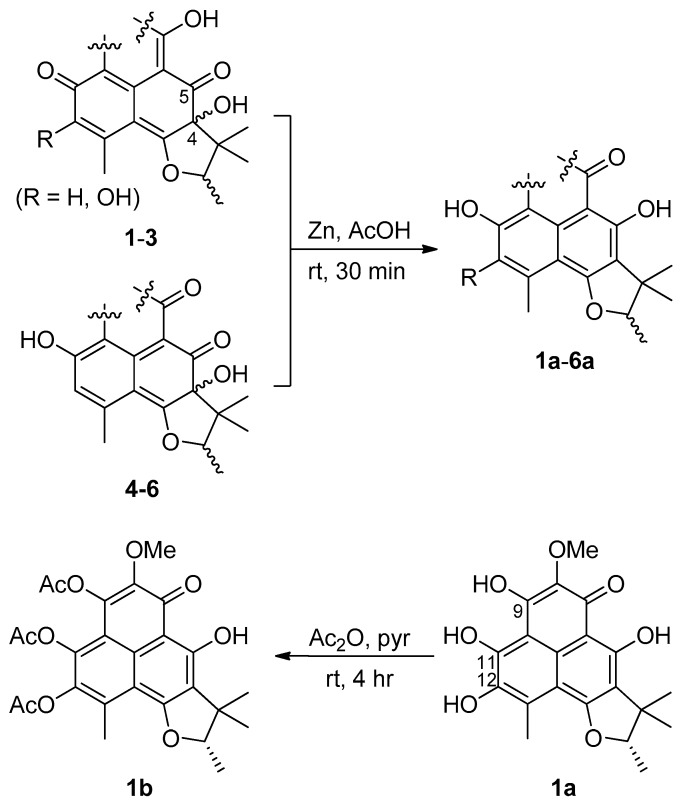
Phenolic derivatization of herqueinones.

The planar structure of **1** was found to be the same as that of the recently reported peniciherqueinone from the fungus *Penicillium herquei* [8]. In our study of the configurations of the C-4 and C-2′ stereogenic centers by NOESY analysis (Figure 3), the OH-4, H-2′, and H_3_-5′ protons were oriented toward the same face of the hydrofuran ring based on their mutual cross-peaks. The opposite face was occupied by H_3_-1′ and H_3_-4′ based on the cross peak between the methyl protons, suggesting that **1** has the same relative configuration (4S* and 2′S*) as peniciherqueinone. Interestingly, despite the same signs of optical rotations, there was a remarkable difference in their values of the specific rotations: [α]${}_{D}^{25}$ (CHCl_3_) +203 (**1**) and +92 (peniciherqueinone). Since the absolute configurations at C-4 and C-2′ of herqueinones have been the subject of comprehensive investigations [9,10], the discrepancy in the specific rotations of **1** and herqueinones needed to be justified. Using a pre-established chemical modification technique [11,12,13], **1** was reduced to **1a**, which showed a negative specific rotation ([α]${}_{D}^{25}$ (CHCl_3_) −23); thus, the 2′*S* configuration was confirmed. The absolute configuration was further evaluated via the acetylation of **1a** to corresponding 9,11,12-triacetyl derivative **1b** (Figure 4). The sign of the specific rotation of **1b** ([α]${}_{D}^{25}$ (MeOH) −42) was opposite to that of herqueinone (**8**) but the same as that of isoherqueinone (**9**), which proved a 2′*S* configuration [9,10]. Therefore, the absolute configuration of **1** was assigned as 4*S* and 2′*S*. Thus, **1**, designated as *ent*-peniciherqueinone, is a new herqueinone-type phenalenone.

The molecular formula of **2** was deduced to be C_19_H_18_O_8_ based on HRFABMS analysis. The NMR data of this compound were very similar to those of **1**, with the absence of a methyl group. A detailed examination of the ^13^C and ^1^H NMR data revealed that the OMe-8 of **1** (δ_C_ 60.9, δ_H_ 3.92) was replaced by a hydroxyl group (δ_H_ 8.96) in **2**, and this assignment was confirmed by a combination of two-dimensional (2D) NMR analyses. The NOESY data and specific rotation of the reduction product **2a** indicated the same 4*S* and 2′*S* configuration as in **1**. Thus, **2**, designated as 12-hydroxynorherqueinone, was determined to be 8-demethyl-*ent*-peniciherqueinone.

Compound **3** was isolated as an orange amorphous solid with a molecular formula of C_20_H_20_O_7_, based on HRFABMS analysis. The ^13^C and ^1^H NMR data of this compound were similar to those obtained for **1**. The most noticeable difference was the replacement of a hydroxyl-bearing olefinic carbon with the sp^2^ methine carbons (δ_C_ 122.8, δ_H_ 6.36). The structural difference was found to be at C-12 based on the HMBC correlations from H-12 (δ_H_ 6.36) to C-2 (δ_C_ 103.0), C-10 (δ_C_ 109.2), and C-14 (δ_C_ 23.8) as well as from H_3_-14 (δ_H_ 2.48) to C-2 (δ_C_ 103.0), C-12 (δ_C_ 122.8), and C-13 (δ_C_ 150.9). However, the sign of the specific rotation of **3** ([α]${}_{D}^{25}$ (MeOH) −69) was opposite to those of **1** and **2**, implying a configurational difference. Since the NOESY spectrum showed the same cross-peaks for the hydrofuran moiety as those in the congeners, **3** was proposed to possess the opposite absolute configuration at C-4 and C-2′. As the reduction product of **3** (**3a**) is dextrorotatory (specific rotation ([α]${}_{D}^{25}$ (MeOH) +39)), the configuration of C-4 and C-2′ are 4*R*, 2′*R*, respectively. Thus, **3**, designated as *ent*-isoherqueinone, is a new herqueinone-type phenalenone derivative.

The molecular formula of **4** was also established as C_22_H_22_O_8_ by HRFABMS analysis. Although its spectroscopic data resembled those of **1**–**3**, several differences were found in both ^13^C and ^1^H NMR data. First, aided by the HSQC data, it was found that three additional carbons, i.e., one carbonyl (δ_C_ 206.9), one methylene sp^2^ (δ_C_ 48.5, δ_H_ 3.30), and one methyl (δ_C_ 29.8, δ_H_ 2.09), were present in this compound (Table 1 and Table 2). In the ^13^C NMR spectrum, resonances of three ketone groups (δ_C_ 200.6, 193.1, and 189.9) were found for **4**, unlike **1**–**3**. In addition, an aromatic or olefinic carbon had been replaced by an oxygen-bearing nonprotonated sp^3^ carbon (δ_C_ 76.9). A detailed examination of its NMR data revealed that **4** contained the same B and D rings as **1**–**3**, and the structural differences were located in the remaining portion of the molecule.

The planar structure of **4** was established by extensive HMBC experiments (Figure 2). Several HMBC correlations were found from an aromatic proton (δ_H_ 6.75, H-12) and a benzylic methyl proton (δ_H_ 2.57, H_3_-14) to their neighboring carbons (H-12 (δ_H_ 6.75) to C-2 (δ_C_ 116.3), C-10 (δ_C_ 109.4), and C-14 (δ_C_ 23.5); H_3_-14 (δ_H_ 2.57) to C-2 (δ_C_ 116.3), C-12 (δ_C_ 117.5), and C-13 (δ_C_ 152.3)). Aided by the D-HMBC correlations from H-12 (δ_H_ 6.75) to C-1 (δ_C_ 142.7) and C-11 (δ_C_ 165.6) and from H_3_-14 (δ_H_ 2.57) to C-1 (δ_C_ 142.7), the long-range carbon–proton correlations led to the establishment of a hydroxyl- and methyl-bearing pentasubstituted benzene as ring B. Additional D-HMBC correlations from these protons to the conspicuous H_3_-1′ at δ_H_ 1.30 (H-12 (δ_H_ 6.75) to C-6 (δ_C_ 101.2); H_3_-14 (δ_H_ 2.57) to C-3 (δ_C_ 175.1), C-5 (δ_C_ 193.1), and C-6 (δ_C_ 101.2); H_3_-1′ (δ_H_ 1.30) to C-3 (δ_C_ 175.1) and C-5 (δ_C_ 193.1)) defined ring C as a hydroxyl-bearing cyclohexadienone. The ring D was found to be the same as that in other herqueinones by a 2D NMR spectrum.

The remaining portion of **4** consists of three ketone carbonyl (δ_C_ 206.9, 200.6, and 189.9) and one nonprotonated sp^3^ (δ_C_ 76.9), one methylene sp^3^ (δ_C_ 48.5), and one methyl (δ_C_ 29.8) carbons. These carbons were initially assembled into a 2-keto-propyl group (C-6′–C-8′) by the HMBC correlations from the methylene and methyl protons to their neighboring carbons (H_2_-6′ (δ_H_ 3.30) to C-7′ (δ_C_ 206.9) and C-8′ (δ_C_ 29.8); H_3_-8′ (δ_H_ 2.09) to C-6′ (δ_C_ 48.5), and C-7′ (δ_C_ 206.9)) (Figure 2). Then, this fragment was connected to the core structure by the HMBC correlations from H_2_-6′ (H_2_-6′ (δ_H_ 3.30) to C-7 (δ_C_ 189.9), C-8 (δ_C_ 76.9), and C-9 (δ_C_ 200.6)). The confirmation of this assignment as well as the linkage with the B-ring was accomplished by the key D-HMBC correlations from OH-8 (δ_H_ 6.68) to C-7 (δ_C_ 189.9) and C-9 (δ_C_ 200.6); from H-12 (δ_H_ 6.75) to C-9 (δ_C_ 200.6); from H_3_-14 (δ_H_ 2.57) to C-9 (δ_C_ 200.6); and from H_3_-8′ (δ_H_ 2.09) to C-8 (δ_C_ 76.9). Although it could not be confirmed by 2D-NMR-based carbon–proton correlations, the presence of the four rings, required by the molecular formula and NMR data, directly connected C-6 and C-7 carbonyl carbons to be part of a diketo-bearing six-membered ring as ring A. Thus, the structure of **4** was determined to be a phenalenone related to an acetone adduct of a triketone [14,15].

The molecular formula of **5** was the same as that of **4**, C_22_H_22_O_8_. Moreover, the ^13^C and ^1^H NMR data of these compounds were very similar (Table 1 and Table 2). Two-dimensional NMR analyses showed the same carbon–proton correlations throughout the entire molecule, indicating that they have the same planar structure. Therefore, **5** could be an epimer of **4**.

**Figure 5:**
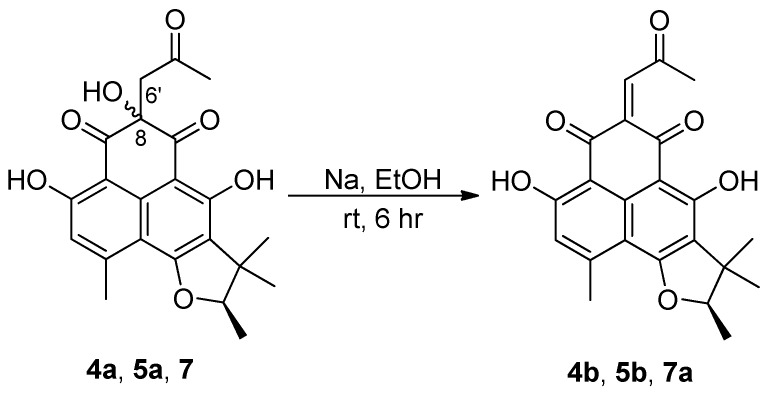
Dehydrations of **4a**, **5a**, and **7**.

In order to clarify the difference in stereochemistry between **4** and **5**, NOESY experiments were carried out. The NOESY spectra of both compounds showed the same cross-peaks around the D ring as those observed in other herqueinones, suggesting the 4*R*,2′*R* or 4*S*,2′*S* configurations. Then, by chemical conversions to remove the other two stereogenic centers, the absolute configurations at C-4 were determined. That is, **4** and **5** were reduced to **4a** and **5a**, respectively (Figure 4), then the compounds were dehydrated to yield 8,15-unsaturated derivatives **4b** and **5b**, respectively (Figure 5), and the MS and NMR data of these compounds were identical. Furthermore, their specific rotations were also very similar ([α]${}_{D}^{25}$ (CHCl_3_) −27 and −26 for **4b** and **5b**, respectively), implying that these were indeed the same compound. The negative specific rotations allow us to confidently assign the 2′*R* configuration for both natural products. Thus, **4** and **5**, designated as oxopropylisoherqueinones A and B respectively, were elucidated as new phenalenones possessing C_3_ side chains. These compounds possessed 4*R*, 2′*R* configurations. However, the configurations at C-8 remain unassigned despite various chemical and spectroscopic analyses.

In order to determine the absolute configurations at C-8 of **4** and **5**, a comparison of the experimental and calculated ECD spectra was carried out. Initially, the experimental CD profiles of these compounds showed opposite signs in the region of 285–340 nm, possibly reflecting the different configuration at C-8 (Supplementary Figure S51). Despite all the efforts, however, the calculated ECD profiles based on the postulated conformational populations failed to assign the absolute configurations satisfactorily (Supplementary Figure S52). This could be due to a weak contribution of a single and remote stereogenic center to the ECD in the molecule possessing several UV chromophores and stereogenic centers.

The molecular formula of **6** was deduced to be C_18_H_16_O_7_, which corresponds to 11 degrees of unsaturation, by HRFABMS analysis. The ^13^C and ^1^H NMR data of this compound revealed that it is a phenalenone derivative based on the presence of signals for two aromatic rings and a trimethylhydrofuran moiety, which account for eight degrees of unsaturation (Table 1 and Table 2). However, only the carbon signals of two nonprotonated quaternary sp^2^ carbons (δ_C_ 164.4 and 155.4) had replaced the NMR signals of the A ring of the other compounds. Therefore, in addition to satisfying the three remaining degrees of unsaturation, the C_2_O_3_ portion must account for two carbonyls and a cyclic ether or ester group.

The planar structure of **6** was determined with the aid of HMBC experiments (Figure 2). First, the long-range couplings of key protons, such as the four methyl groups (H_3_-14 (δ_H_ 2.58), H_3_-1′ (δ_H_ 1.36), H_3_-4′ (δ_H_ 0.78), and H_3_-5′ (δ_H_ 1.26)), an aromatic proton (H-12 (δ_H_ 6.81)), and two hydroxy protons (OH-4 (δ_H_ 7.41) and OH-11 (δ_H_ 11.43)), with their neighboring carbons confirmed the presence of the same B-D polycyclic moiety as in **4** and **5**. An additional coupling to OH-11 placed a carbonyl carbon (δ_C_ 164.4) at C-9, which was supported by the key D-HMBC correlation from H_3_-14 (δ_H_ 2.58) to C-9 (δ_C_ 164.4). The other carbon (δ_C_ 155.5) must be located at C-7 due to the shielding of C-6 (δ_C_ 92.3). Although it was not directly proved by NMR spectra, both the MS data and the shielded chemical shifts of the C-7 and C-9 carbonyls were indicative of an oxygen bridge between these positions, leading to a six-membered cyclic acid anhydride moiety as ring A. The NMR data of the ring portion of **6** were similar to those of sclerodin (**10**), which was previously reported from the fungus *Gremmeniella abietina* thus supporting the structure of **6** [14,16].

The NOESY correlations of **6** placed the OH-4, H-2′, and H_3_-5′ on one side and H_3_-1′ and H_3_-4′ on the other side of the hydrofuran moiety, leading to the same relative configuration (4S* and 2′S*) as that in **1**–**3**. Then, the specific rotation of **6** was similar to that of **3** ([α]${}_{D}^{25}$ −69 and −52 for **3** and **6**, respectively), suggesting they have the same absolute configuration (4*R* and 2′*R*). However, to remove the effect of structural differences in ring A, the reduction of **6** produced the 4-deoxy derivative **6a** (=**10**), which possessed only the C-2′ stereogenic center (Figure 4). Interestingly, the specific rotation of **6a** showed the same sign as those of **1a** and **2a** but opposite to those of **3a** ([α]${}_{D}^{25}$ +34 and −18 for **3a** and **6a**, respectively), confirming the 2′*S* configuration. Our results were in good agreement with the specific rotations of natural **6a** (**10**) and 2′-*epi*-**6a**, which are levorotatory and dextrorotatory, respectively [14]. Overall, the configuration of this compound was assigned as 4*S*,2′*S*. Notably, changing the phenolic A ring to an acid anhydride inverted the sign of the specific rotation of the herqueinone. Thus, **6**, designated as 4-hydroxysclerodin, is a new phenalenone derivative and structurally related to sclerodin (**10**).

In addition to **1**–**6**, five previously reported phenalenones (**7**–**11**) were also isolated. Based on a combination of spectroscopic analyses and a literature survey, these compounds were identified as an acetone adduct of the triketone (**7**) [14], herqueinone (**8**) [3,17,18], isoherqueinone (**9**) [19,20], sclerodin (**10**) [14], and scleroderolide (**11**) [21]. The NMR data of these compounds were in good agreement with the reported values in the literature. Compound **7** was obtained as an unseparated epimeric mixture, which was consistent with the literature [14,15]. Compound **7** was dehydrated to **7a** by the same method used for **4** and **5**, and the 2′*R* configuration was thus assigned. In this way, the epimerization of **7** was found to occur not at C-2′ (in the hydrofuran moiety) but at the hydroxy-bearing C-8 stereogenic center.

Compounds **4**, **5**, and **7** possessed a C_3_ oxopropyl moiety (C-6′–C-8′) whose structural resemblance raised the hypothesis that **7** could be the acetone adduct formed during the separation process. This hypothesis has a reliable experimental basis of chemical transformation of a triketone to **7** [14]. In order to verify if **7** is an acetone adduct or a true natural compound biosynthesized by the fungus, the production of these compounds was monitored by time-scale cultivation and LC-ESI-MS analysis. Weekly mass analysis of the culture media showed that the major metabolite **7** was clearly detected after 6 weeks without using acetone (Supplementary Figure S53). Thus, these compounds were unambiguously proved to be the natural products produced by the *Penicillum* sp. fungus.

Although fungal phenalenones exhibit diverse bioactivities [1,2], herqueinone-type compounds have not frequently shown remarkable bioactivities. The mild antioxidant and radical scavenging activities of isoherqueinone (**9**) [9], the antibacterial activity of scleroderolide (**11**) [22], and human leukocyte elastase inhibition of atrovenetinone can be considered exceptions [23]. Regarding the bioactivities of herqueinones, it is interesting to note that the presence of both OH-5 and OH-11 groups are required for the antibacterial activity [1]. The cytotoxicity assay revealed that **1**–**11** were inactive (IC_50_ > 10 μM) against the K562 (human chronic myeloid leukemia) and A549 (adenocarcinomic human alveolar basal epithelial) cancer cell lines. These compounds were also inactive (MIC > 128 μM) against various bacterial and fungal strains, which was consistent with the report on the structure-activity relationships of herqueinones [1].

Compound **7** moderately inhibited NO production in RAW 264.7 cells with an IC_50_ value of 3.2 μM, while the rest of the isolated compounds were inactive (IC_50_ > 20 μM). In the angiogenesis assay, **6** inhibited tube formation in HUVECs with an IC_50_ of 20.9 μM (Supplementary Table S1 and Figures S54 and S55), while **1** and **9** induced adipogenesis through PPARγ binding and adiponectin secretion-promoting activity in hBM-MSCs and in a concentration-dependent manner, which was determined by adiponectin secretion-promoting effects with their IC_50_ values of 57.5 μM and 39.7 μM, respectively (Table S1 and Figure S56). All of these bioactivities were found to occur without significant cytotoxicity.

In summary, 11 polyketide-derived phenalenones, including six previously unreported phenalenones, were isolated from the culture broth of a marine-derived *Penicillium* sp. The absolute configurations of the stereogenic centers in the hydrofuran ring were assigned by chemical modifications and measurements of specific rotations. Compounds **1**, **6**, **7**, and **9** exhibited diverse bioactivities, such as anti-inflammatory, anti-angiogenetic, and adipogenesis-inducing activities.

## 3. Materials and Methods

### 3.1. General Experimental Procedures

Optical rotations were measured on a JASCO P-1020 polarimeter (Easton, MD, USA) using a cell with a 1-cm path length. UV spectra were acquired using a Hitachi U-3010 spectrophotometer (Tokyo, Japan). CD spectra were recorded on an Applied Photophysics Ltd. Chirascan plus CD spectrometer (Applied Photophysics Ltd., Leatherhead, Surrey, UK). IR spectra were recorded on a JASCO 4200 FT-IR spectrometer (Easton, MD, USA) using a ZnSe cell. NMR spectra were recorded in DMSO-*d_6_* or CDCl_3_ solutions on Bruker Avance-400, -500, or -600 instruments (Billerica, MA, USA). High-resolution FABMS data were acquired using a JEOL JMS 700 mass spectrometer (Tokyo, Japan) with 6 keV-energy, emission current 5.0 mA, xenon as inert gas, and meta-nitrobenzyl alcohol (NBA) as the matrix at the Korea Basic Science Institute (Daegu, Korea). Low-resolution ESIMS data were recorded on an Agilent Technologies 6130 quadrupole mass spectrometer (Santa Clara, CA, USA) with an Agilent Technologies 1200 series HPLC (Santa Clara, CA, USA). HPLC separations were performed on a SpectraSYSTEM p2000 equipped with a refractive index detector (SpectraSYSTEM RI-150 (Waltham, MA, USA)) and a UV-Vis detector (Gilson UV-Vis-151 (Middleton, WI, USA)). All solvents used were of spectroscopic grade or were distilled prior to use.

### 3.2. Fungal Material

The fungal strain *Penicillium* sp. was isolated from marine sediments collected from Gagudo, Korea, in October 2008. The isolate was identified using standard molecular biological protocols by DNA amplification and sequencing of the ITS region. Genomic DNA extraction was performed using Intron’s i-genomic BYF DNA Extraction Mini Kit according to the manufacturer’s protocol. The nucleotide sequence was deposited in the GenBank database under the accession number JF901804. The 18S rDNA sequence of this strain showed 99% identity with *Penicillium herquei* GA4 (GenBank accession number EF536027).

### 3.3. Extraction and Isolation

The fungus was cultivated on YPG medium (5 g of yeast extract, 5 g of peptone, 10 g of glucose in 1 L of artificial seawater) in 2.8 L Fernbach flasks at 30 °C under static conditions in the dark for 6 weeks. The mycelia and culture broth were separated by filtration, and the broth (20 L) was extracted with EtOAc (20 L × 3). The solvent was evaporated under reduced pressure to afford a crude EtOAc extract (6.2 g), which was fractionated by C18 reversed-phase vacuum flash chromatography using mixtures of H_2_O-MeOH, from 50:50 to 0:100, and acetone and EtOAc as the eluents.

Based on the ^1^H NMR and LC-MS analyses, the moderately polar fractions (30:70–10:90 H_2_O-MeOH) were chosen for further separation. The fraction (220 mg) that eluted with H_2_O-MeOH (30:70) was separated by a semi-preparative reversed-phase HPLC (YMC-ODS-A column, 10 × 250 mm; H_2_O-MeOH, 45:55; 1.7 mL/min) to yield **4** (*t*_R_ = 18.4 min, 5.5 mg) and **5** (*t*_R_ = 18.9 min, 7.7 mg). The fraction (570 mg) that eluted with H_2_O-MeOH (20:80) was separated by a semi-preparative reversed-phase HPLC (H_2_O-MeOH, 32:68; 1.7 mL/min) to afford **1** (*t*_R_ = 37.5 min), **2** (*t*_R_ = 27.8 min), **3** (*t*_R_ = 29.1 min), **8** (*t*_R_ = 25.1 min), and **9** (*t*_R_ = 21.8 min). Compounds **1** (311.5 mg), **3** (5.6 mg), **8** (16.5 mg), and **9** (4.4 mg) were purified by an analytical HPLC (YMC-ODS-A column, 4.6 × 250 mm; H_2_O-MeOH, 37:63; 0.7 mL/min; *t*_R_ = 38.8, 34.5, 30.9, and 27.1 min, respectively). Compound **2** (1.7 mg) was also purified by an analytical HPLC (H_2_O-MeCN, 48:52; 0.7 mL/min; *t*_R_ = 35.0 min). The fraction (230 mg) eluted with H_2_O-MeOH (10:90) was separated by a semi-preparative reversed-phase HPLC (H_2_O-MeOH, 22:78; 1.7 mL/min) to yield **7** (*t*_R_ = 19.7 min, 73.4 mg), **10** (*t*_R_ = 22.8 min), and **11** (*t*_R_ = 23.5 min). Compounds **10** (3.9 mg) and **11** (3.3 mg) were further purified by an analytical HPLC (H_2_O-MeOH, 26:74; 0.7 mL/min; *t*_R_ = 26.8 and 30.1 min, respectively).

*ent*-Peniciherqueinone (**1**): red, amorphous solid; [α]${}_{D}^{25}$ +203 (*c* 1.7, CHCl_3_), +254 (*c* 1.0, MeOH); UV (MeOH) λ_max_ (log ε) 217 (4.32), 248 (4.27), 311 (4.20), 427 (3.75) nm; IR (ZnSe) ν_max_ 3413 (br), 1629, 1590, 1385 cm^−1^; ^1^H and ^13^C NMR data, Table 1 and Table 2; HRFABMS *m*/*z* 389.1239 [M + H]^+^ (calcd for C_20_H_21_O_8_, 389.1239).

12-Hydroxynorherqueinone (**2**): red, amorphous solid; [α]${}_{D}^{25}$ +124 (*c* 0.1, MeOH); UV (MeOH) λ_max_ (log ε) 217 (4.32), 248 (4.31), 311 (4.36), 430 (3.80) nm; IR (ZnSe) ν_max_ 3445 (br), 1629, 1579, 1461 cm^−1^; ^1^H and ^13^C NMR data, Table 1 and Table 2; HRFABMS *m*/*z* 375.1079 [M + H]^+^ (calcd for C_19_H_19_O_8_, 375.1080).

*ent*-Isoherqueinone (**3**): orange, amorphous solid; [α]${}_{D}^{25}$ −69 (*c* 0.2, MeOH); UV (MeOH) λ_max_ (log ε) 217 (4.32), 248 (4.29), 311 (4.22), 428 (3.79) nm; IR (ZnSe) ν_max_ 3422 (br), 1631, 1460 cm^−1^; ^1^H and ^13^C NMR data, Table 1 and Table 2; HRFABMS *m*/*z* 373.1285 [M + H]^+^ (calcd for C_20_H_21_O_7_, 373.1283).

Oxopropylisoherqueinone A (**4**): brown, amorphous solid; [α]${}_{D}^{25}$ +92 (*c* 0.2, MeOH); UV (MeOH) λ_max_ (log ε) 224 (4.36), 274 (4.30), 357 (3.57) nm; IR (ZnSe) ν_max_ 3382 (br), 1678, 1639, 1297 cm^−1^; ^1^H and ^13^C NMR data, Table 1 and Table 2; HRFABMS *m*/*z* 415.1396 [M + H]^+^ (calcd for C_22_H_23_O_8_, 415.1393).

Oxopropylisoherqueinone B (**5**): brown, amorphous solid; [α]${}_{D}^{25}$ +43 (*c* 0.2, MeOH); UV (MeOH) λ_max_ (log ε) 224 (4.36), 274 (4.30), 357 (3.57) nm; IR (ZnSe) ν_max_ 3415 (br), 1679, 1640, 1297 cm^−1^; ^1^H and ^13^C NMR data, Table 1 and Table 2; HRFABMS *m*/*z* 415.1396 [M + H]^+^ (calcd for C_22_H_23_O_8_, 415.1393).

4-Hydroxysclerodin (**6**): yellow, amorphous solid; [α]${}_{D}^{25}$ −52 (*c* 0.2, MeOH); UV (MeOH) λ_max_ (log ε) 213 (3.94), 280 (4.21), 312 (3.68) nm; IR (ZnSe) ν_max_ 3424 (br), 3069, 1729, 1460, 1286 cm^−1^; ^1^H and ^13^C NMR data, Table 1 and Table 2; HRFABMS *m*/*z* 345.0977 [M + H]^+^ (calcd for C_18_H_17_O_7_, 345.0974).

### 3.4. Reduction of Herqueinones (***1***–***6***)

To a solution of 44.3 mg (114 μM) of **1** in 0.5 mL of glacial acetic acid was added 100.0 mg (1.53 mM) of zinc dust under nitrogen atmosphere. The mixture was stirred at room temperature for 30 min and filtered through cotton with 1.0 mL of distilled water. The filtrate was left to stand for 45 min and extracted with 1.5 mL of ethyl acetate. Purification by analytical HPLC (YMC-ODS-A column, 4.6 × 250 mm; H_2_O-MeCN (50:50); 0.7 mL/min) afforded the 4-deoxy derivative (**1a**, 6.8 mg) (*t*_R_ = 15.8 min) as a pure compound. Compounds **2**–**6** were reduced in a similar manner.

4-Deoxy-*ent*-peniciherqueinone (**1a**): [α]${}_{D}^{25}$ −23 (*c* 0.5, CHCl_3_); ^1^H NMR (CDCl_3_, 400 MHz) δ_H_ 13.14 (1H, s), 13.10 (1H, s), 13.07 (1H, s), 4.57 (1H, q, *J* = 6.5 Hz), 3.99 (3H, s), 2.71 (3H, s), 1.50 (3H, s), 1.44 (3H, d, *J* = 6.5 Hz), 1.25 (3H, s); ESIMS *m*/*z* 373.1 [M + H]^+^ (calcd for C_20_H_21_O_7_, 373.1).

4-Deoxy-12-hydroxynorherqueinone (**2a**): [α]${}_{D}^{25}$ −20 (*c* 0.3, CHCl_3_); ^1^H NMR (DMSO-*d*_6_, 400 MHz) δ_H_ 14.32 (1H, s), 13.52 (1H, s), 9.28 (1H, s), 8.72 (1H, s), 4.64 (1H, q, *J* = 6.5 Hz), 2.66 (3H, s), 1.49 (3H, s), 1.38 (3H, d, *J* = 6.5 Hz), 1.26 (3H, s); ESIMS *m*/*z* 359.1 [M + H]^+^ (calcd for C_19_H_19_O_7_, 359.1).

4-Deoxy-*ent*-isoherqueinone (**3a**): [α]${}_{D}^{25}$ +34 (*c* 0.5, CHCl_3_); ^1^H NMR (DMSO-*d*_6_, 400 MHz) δ_H_ 13.52 (1H, s), 8.13 (1H, s), 7.48 (1H, br s), 7.14 (1H, br s), 4.69 (1H, q, *J* = 6.5 Hz), 3.13 (3H, s), 2.66 (3H, s), 1.47 (3H, s), 1.42 (3H, d, *J* = 6.5 Hz), 1.22 (3H, s); ESIMS *m*/*z* 357.3 [M + H]^+^ (calcd for C_20_H_21_O_6_, 357.3).

4-Deoxy-oxopropylisoherqueinone A (**4a**): [α]${}_{D}^{25}$ +8 (*c* 0.5, CHCl_3_), +10 (*c* 0.5, MeOH); ^1^H NMR (DMSO-*d*_6_, 400 MHz) δ_H_ 13.27 (1H, s), 8.48 (1H, s), 6.18 (1H, s), 5.73 (1H, s), 4.13 (1H, q, *J* = 6.5 Hz), 3.16 (2H, s), 2.80 (3H, s), 2.05 (3H, s), 1.35 (3H, s), 1.25 (3H, d, *J* = 6.5 Hz), 1.05 (3H, s); ESIMS *m*/*z* 399.1 [M + H]^+^ (calcd for C_22_H_23_O_7_, 399.1).

4-Deoxy-oxopropylisoherqueinone B (**5a**): [α]${}_{D}^{25}$ +3 (*c* 0.5, CHCl_3_), +5 (*c* 0.5, MeOH); ^1^H NMR (DMSO-*d*_6_, 400 MHz) δ_H_ 13.27 (1H, s), 8.47 (1H, s), 6.18 (1H, s), 5.71 (1H, s) 4.10 (1H, q, *J* = 6.5 Hz), 3.16 (2H, s), 2.80 (3H, s), 2.05 (3H, s), 1.34 (3H, s), 1.26 (3H, d, *J* = 6.5 Hz), 1.05 (3H, s); ESIMS *m*/*z* 399.1 [M + H]^+^ (calcd for C_22_H_23_O_7_, 399.1).

Sclerodin (**6a** = **10**): [α]${}_{D}^{25}$ −18 (*c* 0.5, CHCl_3_); ^1^H NMR (CDCl_3_, 400 MHz) δ_H_ 11.5 (1H, s), 6.75 (1H, s), 5.06 (1H, q, *J* = 6.5 Hz), 2.69 (3H, s), 1.48 (3H, d, *J* = 6.5 Hz), 1.41 (3H, s), 0.92 (3H, s); ESIMS *m*/*z* 329.1 [M + H]^+^ (calcd for C_18_H_17_O_6_, 329.1).

### 3.5. Acetylation of 4-Deoxy-ent-peniciherqueinone (***1a***)

To a solution of 3.0 mg (2.7 mM) of **1a** in 3.0 mL of pyridine was added 0.4 mL of Ac_2_O. After stirring the mixture for 4 h at room temperature, the pyridine and excess Ac_2_O were removed under vacuum. Purification by analytical HPLC (YMC-ODS column, 4.6 × 250 mm; 0.7 mL/min; H_2_O-MeCN (40:60)) yielded 4-deoxy-9,11,12-triacetyl-*ent*-peniciherqueinone (**1b**) (*t*_R_ = 35.8 min): [α]${}_{D}^{25}$ −35 (*c* 0.5, CHCl_3_), −42 (*c* 0.5, MeOH); ^1^H NMR (CDCl_3_, 400 MHz) δ_H_ 4.72 (1H, q, *J* = 6.5 Hz), 4.05 (3H, s), 2.71 (3H, s), 2.404 (3H, s), 2.401 (3H, s), 2.39 (3H, s), 1.59 (3H, s), 1.50 (3H, d, *J* = 6.5 Hz), 1.35 (3H, s); ESIMS m/z 499.5 [M + H]^+^ (calcd for C_26_H_27_O_10_, 499.5).

### 3.6. Dehydration of Herqueinones (***4a***, ***5a***, and ***7***)

To a solution of 0.5 mg (44 mM) of Na in 500 μL of anhydrous ethanol was added 1.5 mg (7.5 mM) of **4a** under nitrogen atmosphere. After stirring the mixture for 6 h at room temperature, the solvent was removed under vacuum. Purification by analytical HPLC (YMC-ODS column, 4.6 × 250 mm; 0.7 mL/min; H_2_O-MeCN (40:60)) afforded the 8(6′)-dehydroxy derivative (**4b**) (*t*_R_ = 12.2 min) as a pure compound. Compounds **5a** and **7** were dehydrated to **5b** and **7a**, respectively, in the same manner.

4-Deoxy-8(6′)-dehydroxyoxopropylisoherqueinone A (**4b**): [α]${}_{D}^{25}$ −27 (*c* 0.5, CHCl_3_); ^1^H NMR (CDCl_3_, 400 MHz) δ_H_ 13.31 (1H, s), 12.79 (1H, s), 6.33 (1H, s), 5.61 (1H, s) 4.42 (1H, q, *J* = 6.5 Hz), 2.56 (3H, s), 2.38 (3H, s), 1.46 (3H, s), 1.38 (3H, d, *J* = 6.5 Hz), 1.20 (3H, s); ESIMS *m*/*z* 381.1 [M + H]^+^ (calcd for C_22_H_21_O_6_, 381.1).

4-Deoxy-8(6′)-dehydroxyoxopropylisoherqueinone B (**5b**): [α]${}_{D}^{25}$ −26 (*c* 0.5, CHCl_3_); ^1^H NMR (CDCl_3_, 400 MHz) δ_H_ 13.31 (1H, s), 12.79 (1H, s), 6.33 (1H, s), 5.61 (1H, s) 4.42 (1H, q, *J* = 6.5 Hz), 2.56 (3H, s), 2.38 (3H, s), 1.46 (3H, s), 1.38 (3H, d, *J* = 6.5 Hz), 1.20 (3H, s); ESIMS *m*/*z* 381.1 [M + H]^+^ (calcd for C_22_H_21_O_6_, 381.1).

8(6′)-Dehydroxy derivative of **7** (**7a**): [α]${}_{D}^{25}$ −26 (*c* 0.5, CHCl_3_); ^1^H NMR (CDCl_3_, 400 MHz) δ_H_ 13.31 (1H, s), 12.79 (1H, s), 6.33 (1H, s), 5.61 (1H, s) 4.42 (1H, q, *J* = 6.5 Hz), 2.56 (3H, s), 2.38 (3H, s), 1.46 (3H, s), 1.38 (3H, d, *J* = 6.5 Hz), 1.20 (3H, s); ESIMS *m*/*z* 381.1 [M + H]^+^ (calcd for C_22_H_21_O_6_, 381.1).

### 3.7. ECD Calcualtions

The conformational searches for the C-8 position of **4** and **5** were performed using Macromodel (Version 9.9, Schrodinger LLC.) software with “Mixed torsional/Low Mode sampling” in the GAFF force field. The experiments were conducted in the gas phase with a 50 kJ/mol energy window limit and a maximum of 10,000 steps to thoroughly examine all low-energy conformers. The Polak–Ribiere conjugate gradient (PRCG) method was utilized for minimization processes with 10,000 maximum iterations and a 0.001 kJ (mol Å)^−1^ convergence threshold on the RMS gradient. Conformers within 10 kJ/mol of each global minimum for *R* and *S* form of **4** and **5** were used for gauge-independent atomic orbital (GIAO) shielding constant calculations without geometry optimization employing TmoleX Version 4.2.1 (COSMOlogic GmbH & Co. KG, Leverkusen, Germany) at the B3LYP/6-31G(d,p) level in the gas phase. The ECD spectra were simulated by overlapping each transition, where *σ* is the width of the band at 1/*e* height. Δ*E_i_* and *R_i_* are the excitation energies and rotatory strengths, respectively, for transition *i*. In the current work, the value was 0.10 eV.

$$\Delta \in \left(E\right)=\frac{1}{2.297\times 10^{-39}}\frac{1}{\surd 2\pi \sigma }\overset{A}{\underset{i}{\sum }}\Delta E_{i}R_{i}e^{\left[-{\left(E-\Delta E_{i}\right)}^{2}/{\left(2\sigma \right)}^{2}\right]}$$

### 3.8. Cytotoxicity and Antibacterial Assays

The cytotoxicity assay was performed in accordance with the published protocols [24]. The antimicrobial assay was performed according to the method described previously [25].

#### 3.8.1. iNOS Assay

Mouse macrophage RAW 264.7 cells obtained from the American Type Culture Collection (ATCC, Rockville, MD, USA) were cultured in Dulbecco’s modified Eagle’s medium (DMEM) supplemented with 10% heat-inactivated fetal bovine serum (FBS) with antibiotics-antimycotics (PSF; 100 units/mL penicillin G sodium, 100 ng/mL streptomycin, and 250 ng/mL amphotericin B) [26,27]. The cells were seeded in 24-well plates (2 × 10^5^ cells/mL). The next day, the culture media was changed to 1% FBS-DMEM, and the samples were treated with the test compounds. After pretreatment with the drug for 1 h, 1 μg/mL lipopolysaccharides (LPS) was added to stimulate NO production. The cells were incubated for an additional 18 h, and the amount of NO produced in the supernatant was determined by Griess reaction. Then, the absorbance was measured at 540 nm, and the nitrite concentration was determined by comparison with a sodium nitrite standard curve. The percent inhibition was calculated using the following formula: [1 − (NO level of test samples/NO levels of vehicle-treated control)] × 100. The IC_50_ values were calculated through nonlinear regression analysis using TableCurve 2-D v5.01 (Systat Software Inc., San Jose, CA, USA). At the same time, MTT assays were also performed to test cell viability. MTT solution (final concentration of 500 μg/mL) was added to the cells, and they were incubated for 4 h at 37 °C. The culture media was removed, and the remaining dyes were dissolved in DMSO. The absorbance of each well was measured at 570 nm using a VersaMax ELISA microplate reader (Molecular Devices, Sunnyvale, CA, USA). The percent survival was determined by comparison with a control group (LPS+).

#### 3.8.2. Tube Formation Assay

Human umbilical vascular endothelial cells (HUVECs) were purchased from the American Type Culture Collection (ATCC, Rockville, MD, USA), and cultured in EGM-2 (Lonza, Walkerswille, MD, USA) supplemented with 10% FBS and antibiotics-antimycotics (PSF) [28,29]. The cells were maintained at 37 °C under a humidified atmosphere containing 5% CO_2_. A 96-well plate was coated with Matrigel (Corning) for 30 min at 37 °C under a humidified atmosphere containing 5% CO_2_. HUVECs (1.8 × 10^4^ cells/well) were mixed with the test compounds in 0.5% FBS EBM-2 medium with VEGF (50 ng/mL) or 0.5% FBS EBM-2 medium only for the VEGF negative control. The cells were incubated for 6 h and photographed using an inverted microscope (Olympus Optical Co. Ltd., Tokyo, Japan). Images were quantified with an angiogenesis analyzer using ImageJ software. Tube formation activity was calculated using the following formula: [(Total segment # (tested compound) − Total segment # (VEGF−)]/[Total segment # (VEGF+) − Total segment # (VEGF−)] × 100 (# stands for tubule segment number). The IC_50_ values were calculated through nonlinear regression analysis using TableCurve 2-D v5.01 (Systat Software Inc., San Jose, CA, USA). Cell viabilities were evaluated with the MTT assay. HUVECs (0.8 × 10^4^ cells/well) were seeded into a 96-well plate and indicated for 1 day. The culture medium was replaced with serum-free medium, and the cells were incubated overnight. After starvation, the cells were treated with the test compounds and VEGF (50 ng/mL) in 2% FBS EBM-2 medium. Cells were incubated for a further 24 h, and MTT solution (final concentration of 500 μg/mL) was added to the cells to measure the cell viability. The formazan products were dissolved in DMSO. The absorbance of each well was measured at 570 nm using a VersaMax ELISA microplate reader (Molecular Devices, Sunnyvale, CA, USA).

#### 3.8.3. Adiponectin Production Assay

Human bone marrow-mesenchymal stem cells (hBM-MSCs) were purchased from Lonza, Inc. (Walkersville, MD, USA) and cultured in low-glucose (1 g/L) DMEM supplemented with 10% FBS, penicillin-streptomycin, and Glutamax^TM^ (Invitrogen, Carlsbad, CA, USA). To induce adipogenesis, the cell growth medium was replaced with high-glucose (4.5 g/L) DMEM supplemented with 10% FBS, penicillin-streptomycin, 10 μg/mL insulin, 0.5 μM dexamethasone, and 0.5 mM 3-isobutyl-1-methylxanthine (IBMX) (IDX conditions) [30]. IBMX, pioglitazone, and aspirin were purchased from Sigma-Aldrich (St. Louis, MO, USA). hBM-MSCs were stained with 0.2% oil red O (ORO) reagent for 10 min at 24 °C, and then washed with H_2_O four times. Following a 10-min elution with isopropanol, the absorbance was measured at 500 nm using a spectrophotometer. To visualize the nucleus, the hBM-MSCs were counterstained with hematoxylin reagent for 2 min and then washed twice with H_2_O. The level of adipocyte differentiation was observed and counted using an inverted phase microscope. A Quantikine immunoassay kit (R&D Systems, Minneapolis, MN, USA) was used for quantitative determination of adiponectin in the cell culture supernatants.

#### 3.8.4. Receptor Binding Assay

The time-resolved fluorescence resonance energy transfer (TR-FRET)-based nuclear receptor binding assay to evaluate binding of the ligand to PPARγ was performed using Lanthanscreen^TM^ competitive binding assay kits (Invitrogen) [30]. All assay measurements were performed using a CLARIOstar instrument (BMG LABTECH, Ortenberg, Germany) with the settings described in the TR-FRET manufacturer’s instructions.

## 4. Conclusions

Six new phenalenone derivatives (**1**–**6**) and five known compounds (**7**–**11**) of the herqueinone class were isolated from a marine-derived fungus *Penicillium* sp. The structure elucidation of compounds **1**–**6** were established by combined spectroscopic methods. The absolute configurations were determined by chemical modifications and their specific rotations. 4-Hydroxysclerodin (**6**) and an acetone adduct of a triketone (**7**) exhibited moderate anti-angiogenetic and anti-inflammatory activities, respectively, while *ent*-peniciherqueinone (**1**) and isoherqueinone (**9**) exhibited moderate abilities to induce adipogenesis without cytotoxicity.

## 5. Patents

Shin, J.; Oh, K.-B. Phenalenone derivatives and antimicrobial composition. KR 2014112273 A 20140923, 2014.

## Figures and Tables

**Table 1:** ^13^C NMR (125 and 150 MHz) of **1**–**6**.

Position	δ_C_, Type						
	1 *^a^*	1 *^b^*	2 *^b^*	3 *^b^*	4 *^b^*	5 *^b^*	6 *^b^*
1	137.3, C	137.1, C	133.5, C	139.0, C	142.7, C	142.9, C	141.8, C
2	103.7, C	103.1, C	102.9, C	103.0, C	116.3, C	116.7, C	115.4, C
3	174.6, C	175.4, C	173.9, C	178.2, C	175.1, C	175.6, C	180.4, C
4	79.0, C	78.4, C	78.2, C	78.5, C	78.5, C	78.5, C	78.9, C
5	197.6, C	198.2, C	198.3, C	197.7, C	193.1, C	193.3, C	192.2, C
6	103.5, C	102.7, C	102.6, C	102.6, C	101.2, C	101.4, C	92.3, C
7	162.8, C	161.7, C	157.2, C	161.9, C	189.9, C	189.3, C	155.5, C
8	131.6, C	131.0, C	129.2, C	131.2, C	76.9, C	75.8, C	
9	161.7, C	161.7, C	156.2, C	163.0, C	200.6, C	200.3, C	164.4, C
10	108.7, C	108.6, C	108.4, C	109.2, C	109.4, C	109.4, C	102.0, C
11	178.1, C	178.7, C	178.7, C	186.4, C	165.6, C	165.8, C	164.0, C
12	143.7, C	144.0, C	143.9, C	122.8, CH	117.5, CH	117.5, CH	117.3, C
13	124.0, C	124.5, C	123.8, C	150.9, C	152.3, C	152.2, C	152.4, C
14	14.9, CH_3_	14.6, CH_3_	14.4, CH_3_	23.8, CH_3_	23.5, CH_3_	23.4, CH_3_	23.2, CH_3_
15	60.9, CH_3_	60.0, CH_3_		60.0, CH_3_			
1′	13.3, CH_3_	12.9, CH_3_	12.9, CH_3_	12.9, CH_3_	12.7, CH_3_	12.8, CH_3_	12.8, CH_3_
2′	89.5, CH	89.3, CH	88.7, CH	90.6, CH	88.6, CH	88.8, CH	90.8, CH
3′	46.9, C	45.9, C	45.9, C	46.0, C	45.6, C	45.3, C	45.7, C
4′	16.4, CH_3_	15.9, CH_3_	15.9, CH_3_	15.9, CH_3_	16.1, CH_3_	16.1, CH_3_	16.0, CH_3_
5′	16.4, CH_3_	16.2, CH_3_	16.1, CH_3_	16.1, CH_3_	16.3, CH_3_	16.3, CH_3_	16.3, CH_3_
6′					48.5, CH_2_	48.6, CH_2_	
7′					206.9, C	207.1, C	
8′					29.8, CH_3_	29.7, CH_3_	

*^a,b^* The spectra were recorded in CDCl_3_ and DMSO-*d*_6_, respectively.

**Table 2:** ^1^H NMR (400 and 600 MHz) of **1**–**6**.

Position	δ_H_, Mult. (*J* in Hz)						
	1 *^a^*	1 *^b^*	2 *^b^*	3 *^b^*	4 *^b^*	5 *^b^*	6 *^b^*
12				6.36, s	6.75, s	6.76, s	6.81, s
14	2.47, s	2.39, s	2.39, s	2.48, s	2.57, s	2.55, s	2.58, s
15	3.92, s	3.77, s		3.77, s			
1′	1.40, d (6.6)	1.35, d (6.3)	1.34, d (6.4)	1.37, d (6.5)	1.30, d (6.4)	1.29, d (6.5)	1.36, d (6.5)
2′	4.99, q (6.6)	4.91, q (6.3)	4.88, q (6.4)	4.99, q (6.5)	4.77, q (6.4)	4.79, q (6.5)	4.92, q (6.5)
4′	1.43, s	1.30, s	1.30, s	0.78, s	0.79, s	0.78, s	0.78, s
5′	0.86, s	0.75, s	0.75, s	1.32, s	1.25, s	1.25, s	1.26, s
6′					3.30, s	3.38, s	
8′					2.09, s	2.12, s	
OH-4	7.23, s	7.38, s	7.28, s	7.52, s	7.15, s	7.23, s	7.41, s
OH-7	13.23, s	13.30, s	13.14, s	13.26, s			
OH-8			8.96, s		6.68, s	6.82, s	
OH-9	13.99, s	14.97, s	14.55, s	15.73, s			
OH-11					12.80, s	12.75, s	11.43, br s
OH-12	6.66, s	9.15, s	9.00, s				

*^a,b^* The spectra were recorded in CDCl_3_ and DMSO-*d*_6_, respectively.

## Supplementary Materials

The following are available online at https://www.mdpi.com/1660-3397/17/3/176/s1. Figures S1–S41: annotated 1D NMR and selected 2D NMR spectra of **1**–**6**, Figures S42–S47: ^1^H NMR spectra of **1a**–**6a**, Figures S48–S50: ^1^H NMR spectra of **4b**, **5b**, and **7a**, Figures S51 and S52: The CD and ECD spectrum of **4**–**5**, Figure S53: The time-scale LC-MS analysis of **7**, Figure S54: iNOS assay and MTT assay, Figure S55: Tube formation assay and MTT assay, Figure S56: adiponectin production assay and PPARγ binding assay, Table S1: The results of bioactivity tests.

## References

[1] Elsebai M.F., Saleem M., Tejesvi M.V., Kajula M., Mattila S., Mehiri M., Turpeinen A., Pirttila A.M. (2014). Fungal phenalenones: chemistry, biology, biosynthesis and phylogeny. Nat. Prod. Rep..

[2] Nazir M., Maddah F., Kehraus S., Egereva E., Piel J., Brachmann A.O., König G.M. (2015). Phenalenones: Insight into the biosynthesis of polyketides from the marine alga-derived fungus *Coniothyrium* cereal. Org. Biomol. Chem..

[3] Galarraga J.A., Neill K.G., Raistrick H. (1955). Colouring matters of *Penicillium herquei*. Biochem. J..

[4] Frost D.A., Halton D.D., Morrison G.A. (1977). Naturally occurring compounds related to phenalenone. Part 8. Structure and synthesis of demethylherqueichrysin. J. Chem. Soc. Perkin Trans..

[5] Narasimhachari N., Vining L.C. (1972). Herqueichrysin, a new phenalenone antibiotic from *Penicillium herquei*. J. Antibiot..

[6] Julianti E., Lee J.-H., Liao L., Park W., Park S., Oh D.-C., Oh K.-B., Shin J. (2013). New Polyaromatic Metabolites from a marine-Derived Fungus *Penicillium* sp.. Org. Lett..

[7] Furihata K., Seto H. (1995). Decoupled HMBC (D-HMBC), an improved technique of HMBC. Tetrahedron Lett..

[8] Tansakul C., Rukachaisirikul V., Maha A., Kongprapan T., Phongpaichit S., Hutadilok-Towatana N., Borwornwiriyapan K., Sakayaroj J. (2014). A new phenalenone derivative from the soil fungus *Penicillium herquei* PSU-RSPG93. J. Nat. Prod. Res..

[9] Barton D.H.R., Mayo P., Morrison G.A., Raistrick H. (1959). The constitutions of atrovenetin and of some related herqueinone derivatives. Tetrahedron.

[10] Brooks J.S., Morrison G.A. (1970). The constitution of herqueinone and its relationship to isoherqueinone. Tetrahedron Lett..

[11] Rani B.R., Ubukata M., Osada H. (1995). Reduction of arylcarbonyl using zinc dust in acetic acid. Bull. Chem. Soc. Jpn..

[12] Bonner T.G., McNamara P. (1968). The pyridine-catalysed acetylation of phenols and alcohols by acetic anhydride. J. Chem. Soc. B.

[13] Lugemwa F.N., Shaikh K., Hochstedt E. (2013). Facile and efficient acetylation of primary alcohols and phenols with acetic anhydride catalyzed by dried Sodium bicarbonate. Catalysts.

[14] Ayer W.A., Hoyano Y., Pedras M.S., Altena I. (1986). Metabolites produced by the Scleroderris canker fungus, *Gremmeniellaabietina*. Part 1. Can. J. Chem..

[15] Krohn K., Sohrab M.D.H., Aust H.-J., Draeger S., Schulz B. (2004). Biologically active metabolites from fungi, 19: New isocoumarins and highly substituted benzoic acids from the endophytic fungus, *Scytalidium* sp.. Nat. Prod. Res..

[16] Homma K., Fukuyama K., Katsube Y., Kimura Y., Hamasaki T. (1980). structure and absolute configuration of an atrovenetin-like metabolite from *Aspergillus silvaticus*. Agric. Biol. Chem..

[17] Stodola F.H., Raper K.B., Fennell D.I. (1951). Pigments of *Penicillium herquei*. Nature.

[18] Suga T., Yoshioka T., Hirata T., Aoki T. (1983). ^13^C NMR Signal Assignments of Herqueinone and Its Phenalenone Derivatives. Bull. Chem. Soc. Jpn..

[19] Harman R.E., Cason J., Stodola F.H., Adkins A. (1955). Structural features of herqueinone, a red pigment from *Penicillium herquei*. J. Org. Chem..

[20] Cason J., Koch C.W., Correia J.S. (1970). Structures of herqueinone, isoherqueinone and norherqueinone. J. Org. Chem..

[21] Ayer W.A., Hoyano Y., Pedras M.S., Clardy J., Arnold E. (1987). Metabolites produced by the scleroderris canker fungus, *Gremmeniellaabietina*. Part 2. The structure of scleroderolide. Can. J. Chem..

[22] Ayer W.A., Kamada M., Ma Y.T. (1989). Sclerodin and related compounds from a plant disease causing fungus. Scleroderris yellow. Can. J. Chem..

[23] Shiomi K., Matsui R., Isozaki M., Chiba H., Sugai T., Yamaguchi Y., Masuma R., Tomoda H., Chiba T., Yan H. (2005). Fungal Phenalenones Inhibit HIV-1 Integrase. J. Antibiot..

[24] Vichai V., Kirtikara K. (2006). Sulforhodamine B colorimetric assay for cytotoxicity screening. Nat. Protoc..

[25] Kim C.-K., Woo J.-K., Kim S.-H., Cho E., Lee Y.-J., Lee H.-S., Sim C.J., Oh D.-C., Oh K.-B., Shin J. (2015). Meroterpenoids from a Tropical *Dysidea* sp.. Sponge. J. Nat. Prod..

[26] Nakane M., Klinghofer V., Kuk J.E., Donnelly J.L., Budzik G.P., Pollock J.S., Basha F., Carter G.W. (1995). Novel potent and selective inhibitors of inducible nitric oxide synthase. Mol. Pharmacol..

[27] Chung H.-J., Koh W., Kim W.K., Shin J.-S., Lee J., Lee S.K., Ha I.-H. (2018). The anti-inflammatory effects of Shinbaro3 is mediated by downregulation of the TLR4 signalling pathway in LPS-stimulated RAW 264.7 macrophages. Mediat. Inflamm..

[28] Carmeliet P., Jain R.K. (2000). Angiogenesis in cancer and other diseases. Nature.

[29] Yu S., Oh J., Li F., Kwon Y., Cho H., Shin J., Lee S.K., Kim S. (2017). New scaffold for angiogenesis inhibitors discovered by targeted chemical transformations of wondonin natural products. ACS Med. Chem. Lett..

[30] Anh S., Lee M., An S., Hyun S., Hwang J., Lee J., Noh M. (2018). 2-Formyl-komarovicine promotes adiponectin production in human mesenchymal stem cells through PPARγ partial agonism. Bioorg. Med. Chem..

