# Supplementary material for: Phenalenones from a Marine-Derived Fungus Penicillium sp
Source: Mar Drugs. 2019 Mar 18;17(3):176. doi: 10.3390/md17030176 (PMC6470642; doi:10.3390/md17030176)
Supplement: Supplementary file 1 [file marinedrugs-17-00176-s001.pdf]

Supporting Information

Phenalenones from a Marine-Derived Fungus *Penicillium* sp.

Sung Chul Park [1], Elin Julianti [1,2], Sungjin Ahn [1], Donghwa Kim [1], Sang Kook Lee [1], Minsoo Noh [1], Dong-Chan Oh [1], Ki-Bong Oh [3,*], and Jongheon Shin [1,*]

[1] Natural Products Research Institute, College of Pharmacy, Seoul National University, San 56-1, Sillim, Gwanak, Seoul 151-742, Korea; sungchulpark@snu.ac.kr (S.C.P.); sungjinahn@snu.ac.kr (S.A.); dkim0719@snu.ac.kr (D.K.); minsoonoh@snu.ac.kr (M.N.); sklee61@snu.ac.kr (S.K.L.); dongchanoh@snu.ac.kr (D.-C.O.); shinj@snu.ac.kr (J.S.)

[2] School of Pharmacy, Bandung Institute of Technology, Jl. Ganesha 10, Bandung 40132, Indonesia; elin_julianti@fa.itb.ac.id (E.J.)

[3] Department of Agricultural Biotechnology, College of Agriculture and Life Science, Seoul National University, San 56-1, Sillim, Gwanak, Seoul 151-921, Korea; ohkibong@snu.ac.kr (K.-B.O.)

* Correspondence: shinj@snu.ac.kr (J.S.); ohkibong@snu.ac.kr (K.-B.O.); Tel.: +82-2-880-2484 (J.S.); +82-2-880-4646 (K.-B.O.)

Contents

Figure S1. The ^{1}H NMR (600 MHz, DMSO-d_6) spectrum of **1** S4
Figure S2. The ^{13}C NMR (150 MHz, DMSO-d_6) spectrum of **1** S5
Figure S3. The COSY (600 MHz, DMSO-d_6) spectrum of **1** S6
Figure S4. The HSQC (600 MHz, DMSO-d_6) spectrum of **1** S7
Figure S5. The HMBC (500 MHz, DMSO-d_6) spectrum of **1** S8
Figure S6. The NOESY (500 MHz, DMSO- d_6) spectrum of **1** S9
Figure S7. The D-HMBC (800 MHz, J_{CH} = 4 Hz, $CDCl_3$) spectrum of **1** S10
Figure S8. The D-HMBC (800 MHz, J_{CH} = 2 Hz, $CDCl_3$) spectrum of **1** S11
Figure S9. The D-HMBC (800 MHz, J_{CH} = 1 Hz, $CDCl_3$) spectrum of **1** S12
Figure S10. The ^{1}H NMR (400 MHz, $CDCl_3$) spectrum of **2** S13
Figure S11. The ^{13}C NMR (125 MHz, $CDCl_3$) spectrum of **2** S14
Figure S12. The COSY (400 MHz, $CDCl_3$) spectrum of **2** S15
Figure S13. The HSQC (400 MHz, $CDCl_3$) spectrum of **2** S16
Figure S14. The HMBC (400 MHz, $CDCl_3$) spectrum of **2** S17
Figure S15. The NOESY (400 MHz, $CDCl_3$) spectrum of **2** S18
Figure S16. The ^{1}H NMR (600 MHz, DMSO-d_6) spectrum of **3** S19
Figure S17. The ^{13}C NMR (150 MHz, DMSO-d_6) spectrum of **3** S20
Figure S18. The COSY (600 MHz, DMSO-d_6) spectrum of **3** S21
Figure S19. The HSQC (600 MHz, DMSO-d_6) spectrum of **3** S22
Figure S20. The HMBC (600 MHz, DMSO-d_6) spectrum of **3** S23
Figure S21. The NOESY (600 MHz, DMSO- d_6) spectrum of **3** S24
Figure S22. The ^{1}H NMR (600 MHz, DMSO-d_6) spectrum of **4** S25
Figure S23. The ^{13}C NMR (150 MHz, DMSO-d_6) spectrum of **4** S26
Figure S24. The COSY (600 MHz, DMSO-d_6) spectrum of **4** S27
Figure S25. The HSQC (600 MHz, DMSO-d_6) spectrum of **4** S28
Figure S26. The HMBC (600 MHz, DMSO-d_6) spectrum of **4** S29
Figure S27. The NOESY (600 MHz, DMSO- d_6) spectrum of **4** S30
Figure S28. The D-HMBC (800 MHz, J_{CH} = 1 Hz, DMSO-d_6) spectrum of **4** S31
Figure S29. The ^{1}H NMR (600 MHz, DMSO-d_6) spectrum of **5** S32
Figure S30. The ^{13}C NMR (150 MHz, DMSO-d_6) spectrum of **5** S33
Figure S31. The COSY (600 MHz, DMSO-d_6) spectrum of **5** S34
Figure S32. The HSQC (600 MHz, DMSO-d_6) spectrum of **5** S35

Figure S33. The HMBC (600 MHz, DMSO-d_6) spectrum of **5** S36
Figure S34. The NOESY (400 MHz, DMSO-d_6) spectrum of **5** S37
Figure S35. The ^{1}H NMR (600 MHz, DMSO-d_6) spectrum of **6** S38
Figure S36. The ^{13}C NMR (150 MHz, DMSO-d_6) spectrum of **6** S39
Figure S37. The COSY (600 MHz, DMSO-d_6) spectrum of **6** S40
Figure S38. The HSQC (600 MHz, DMSO-d_6) spectrum of **6** S41
Figure S39. The HMBC (600 MHz, DMSO-d_6) spectrum of **6** S42
Figure S40. The NOESY (600 MHz, DMSO- d_6) spectrum of **6** S43
Figure S41. The D-HMBC (800 MHz, J_{CH} = 1 Hz, DMSO-d_6) spectrum of **6** S44
Figure S42. The ^{1}H NMR (400 MHz, $CDCl_3$) spectrum of **1a** S45
Figure S43. The ^{1}H NMR (400 MHz, DMSO-d_6) spectrum of **2a** S46
Figure S44. The ^{1}H NMR (400 MHz, DMSO-d_6) spectrum of **3a** S47
Figure S45. The ^{1}H NMR (400 MHz, DMSO-d_6) spectrum of **4a** S48
Figure S46. The ^{1}H NMR (400 MHz, DMSO-d_6) spectrum of **5a** S49
Figure S47. The ^{1}H NMR (400 MHz, $CDCl_3$) spectrum of **6a** S50
Figure S48. The ^{1}H NMR (600 MHz, $CDCl_3$) spectrum of **1b** S51
Figure S49. The ^{13}C NMR (150 MHz, $CDCl_3$) spectrum of **1b** S52
Figure S50. The ^{1}H NMR (400 MHz, $CDCl_3$) spectrum of **4b** S53
Figure S51. The CD spectra of **4-5** S54
Figure S52. The ECD spectra of **4-5** S54
Figure S53. The time-scale LC-MS analysis of **7** S55
Figure S54. iNOS assay and MTT assay S56
Figure S55. Tube formation assay and MTT assay S57
Figure S56. Adiponectin production assay and PPARγ binding assay S58
Table S1. Results of bioactivity tests S59

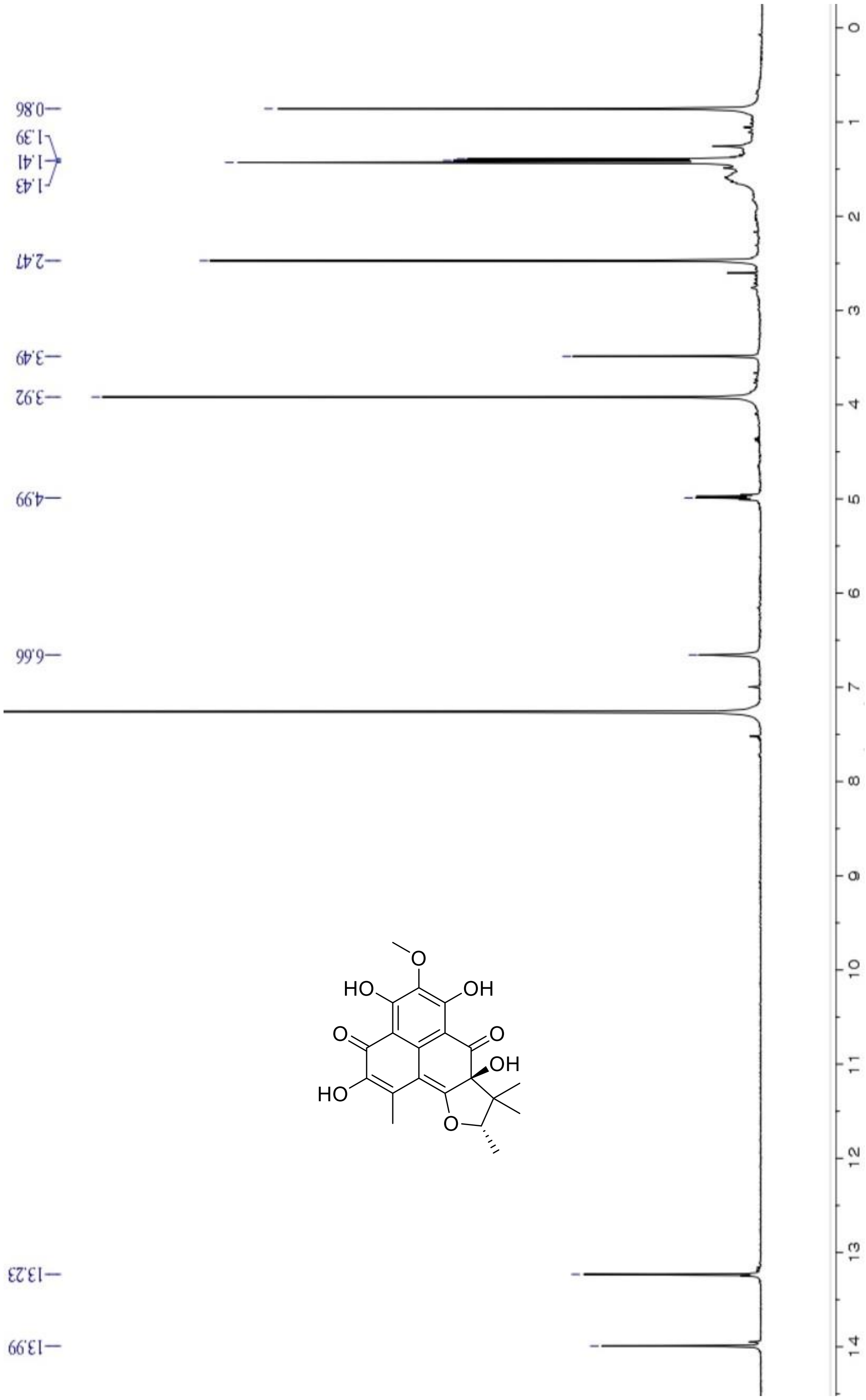

Figure S1. The ^{1}H NMR (400 MHz, $CDCl_3$) spectrum of **1**

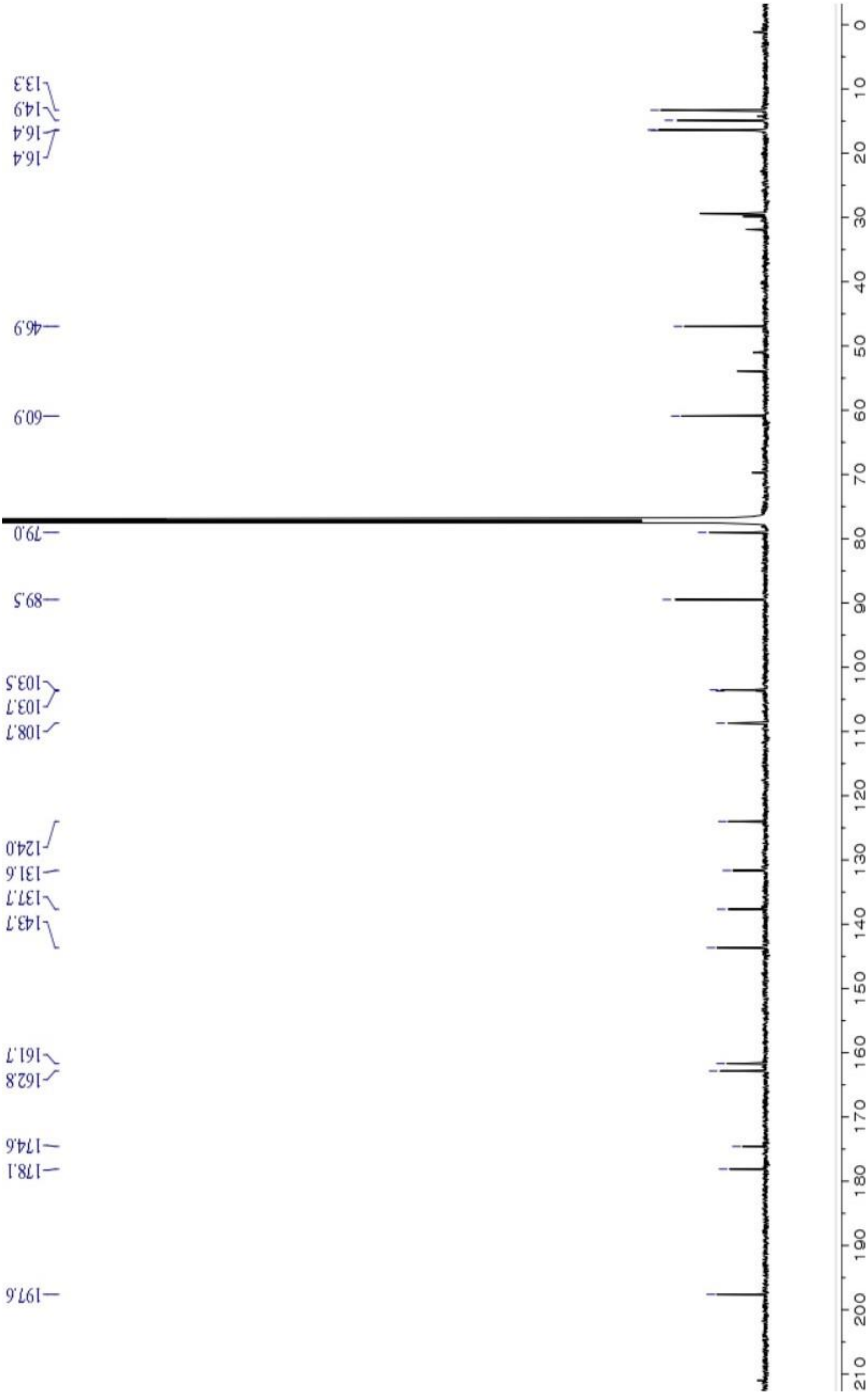

Figure S2. The ^{13}C NMR (125 MHz, $CDCl_3$) spectrum of **1**

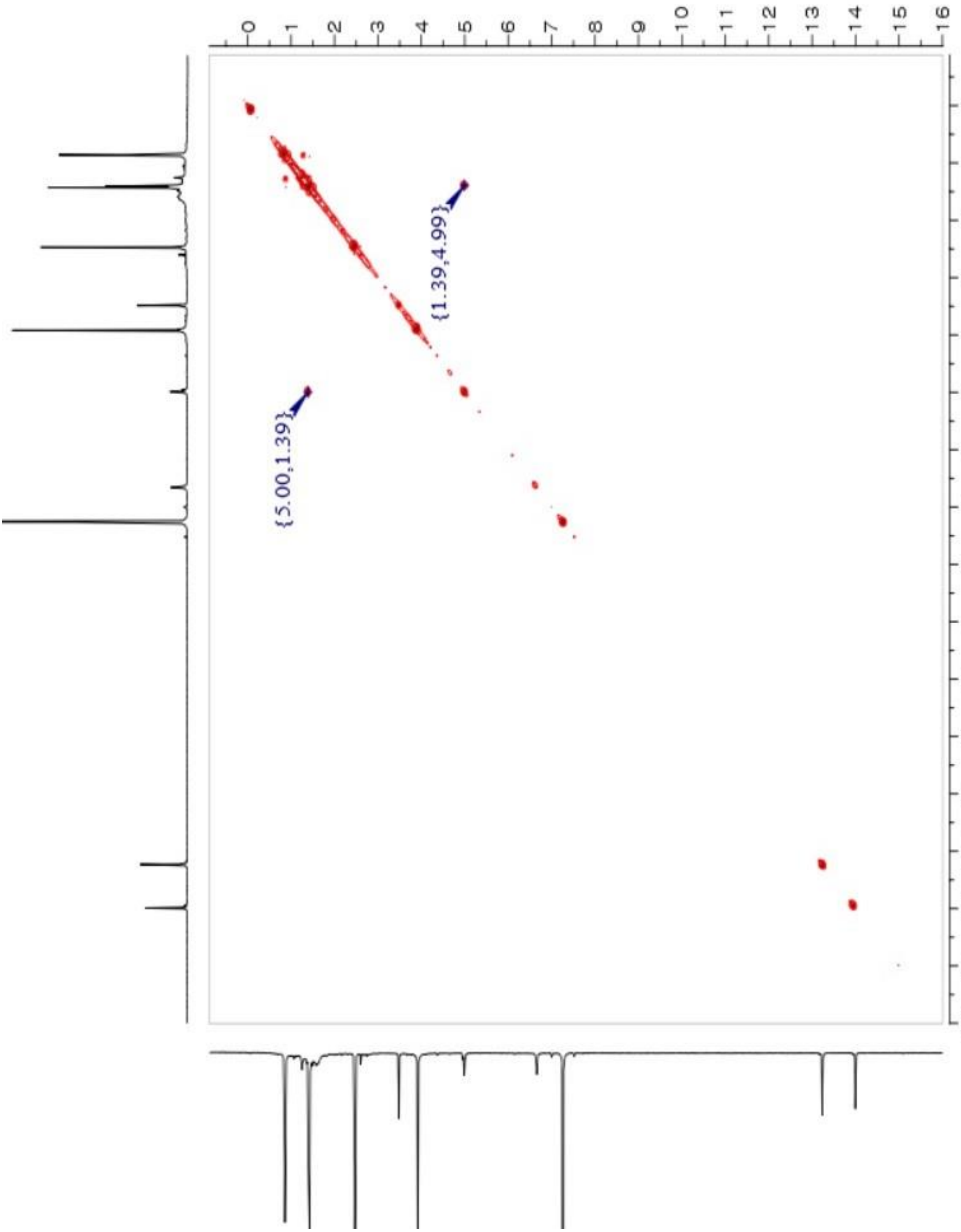

Figure S3. The COSY (400 MHz, $CDCl_3$) spectrum of **1**

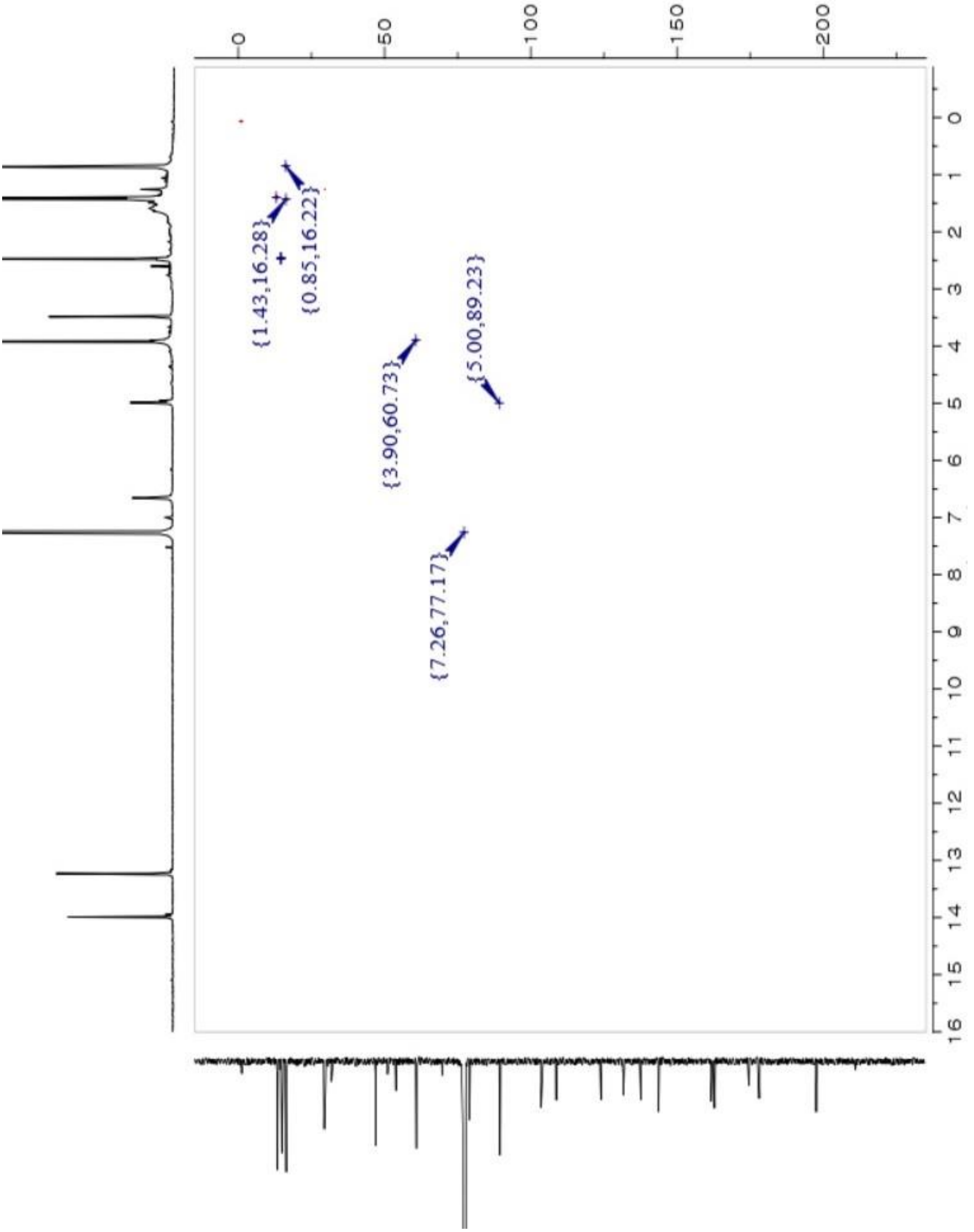

Figure S4. The HSQC (400 MHz, $CDCl_3$) spectrum of **1**

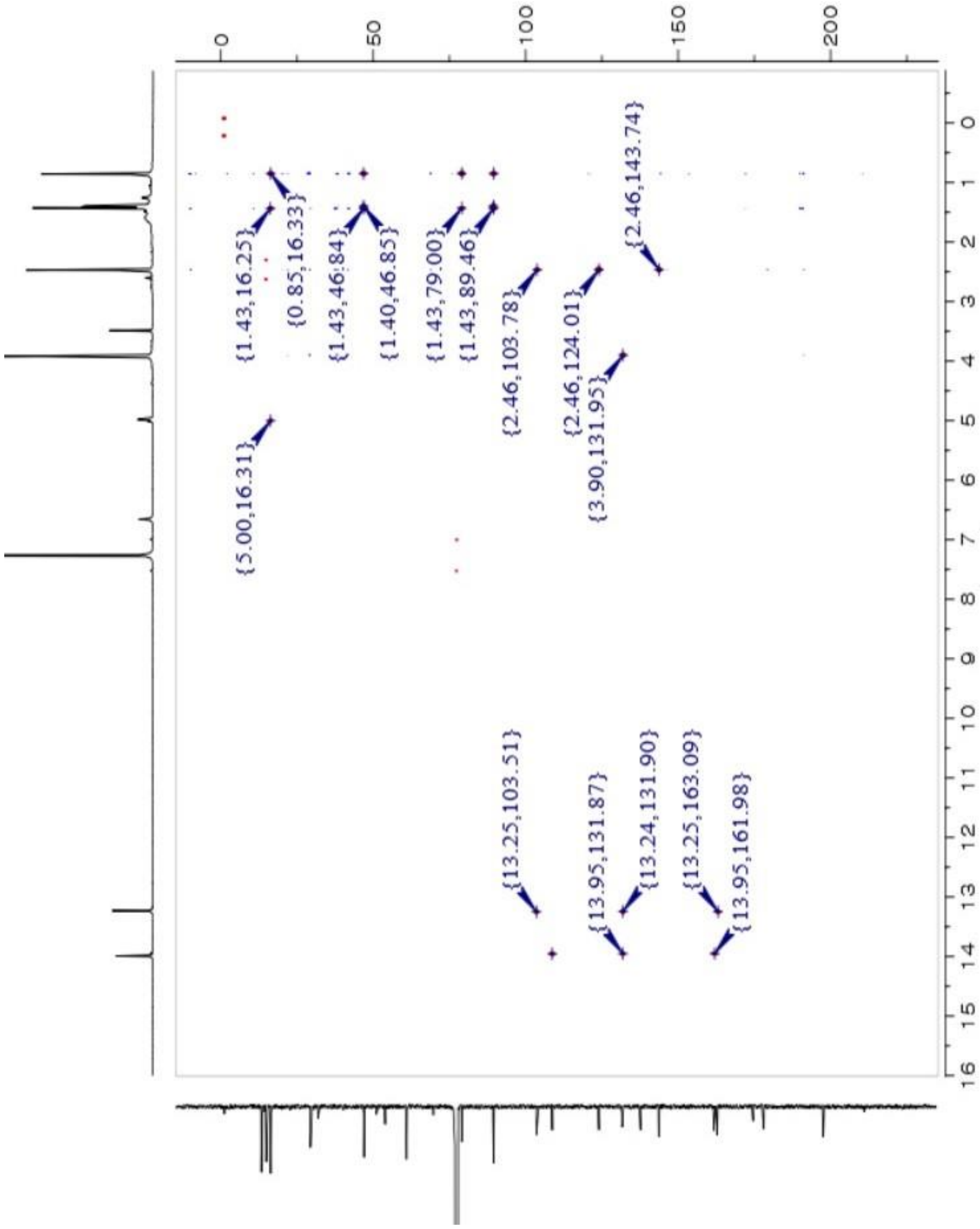

Figure S5. The HMBC (400 MHz, $CDCl_3$) spectrum of **1**

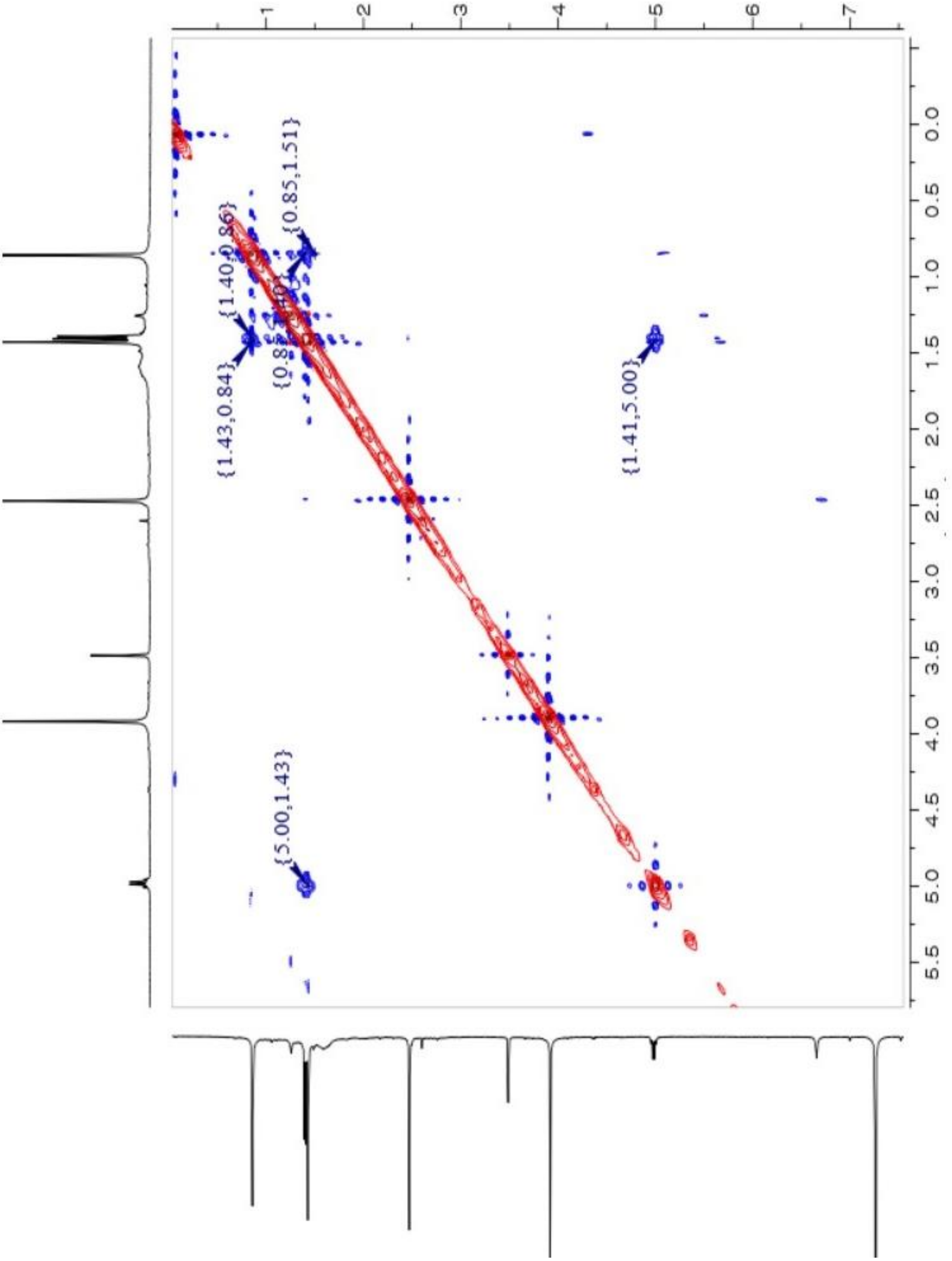

Figure S6. The NOESY (400 MHz, $CDCl_3$) spectrum of **1**

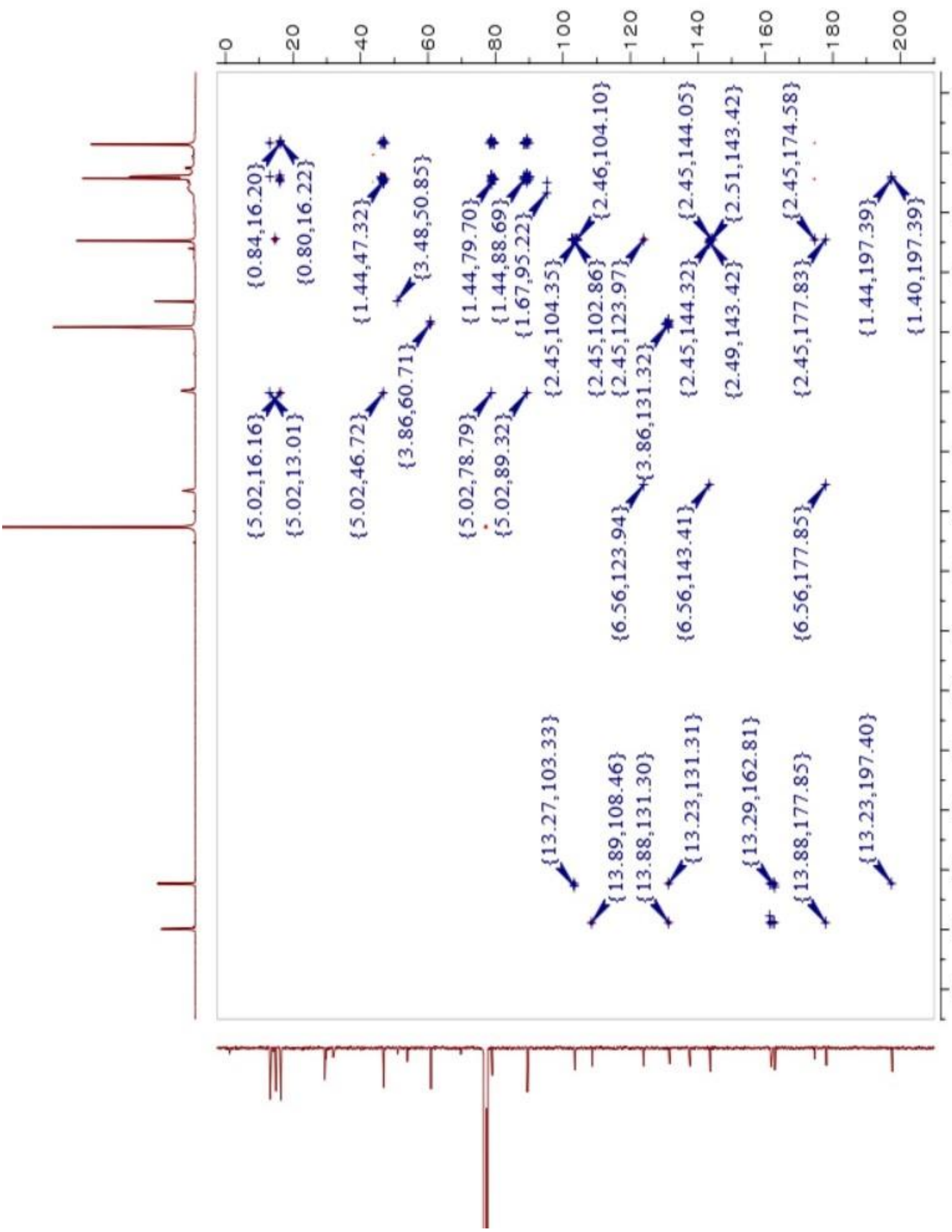

Figure S7. The D-HMBC (800 MHz, J_{CH} = 4 Hz, $CDCl_3$) spectrum of **1**

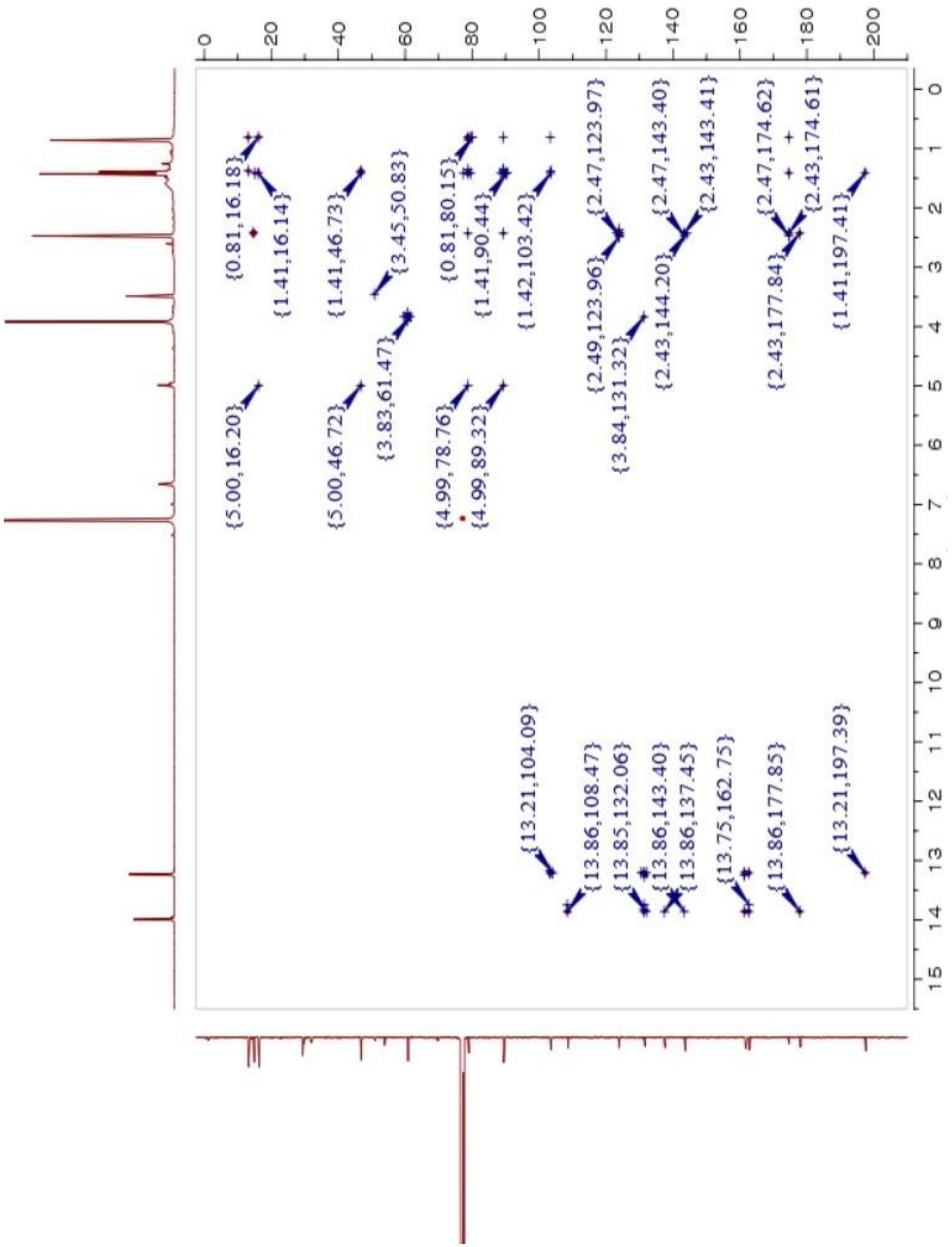

Figure S8. The D-HMBC (800 MHz, J_{CH} = 2 Hz, $CDCl_3$) spectrum of **1**

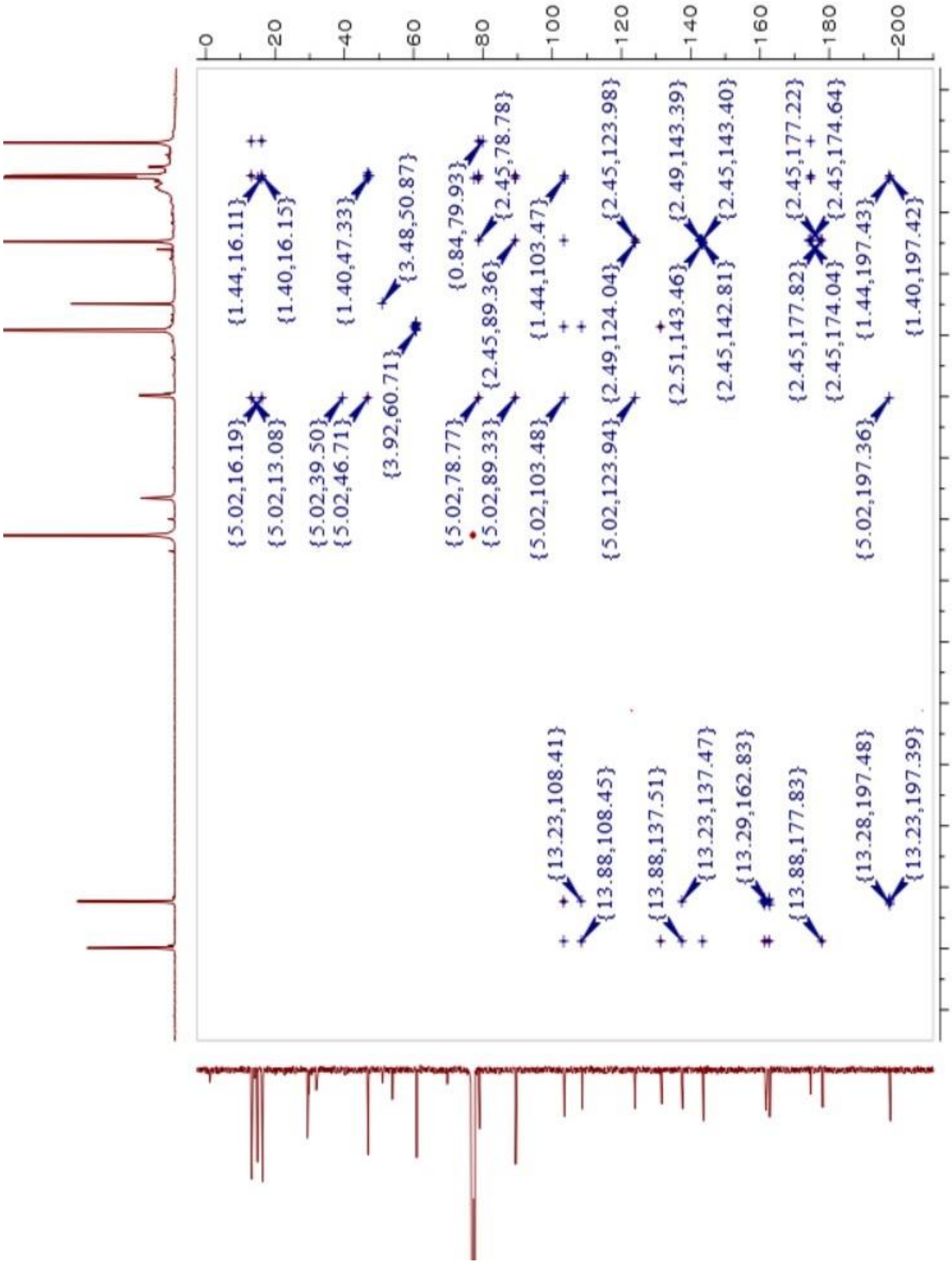

Figure S9. The D-HMBC (800 MHz, J_{CH} = 1 Hz, $CDCl_3$) spectrum of **1**

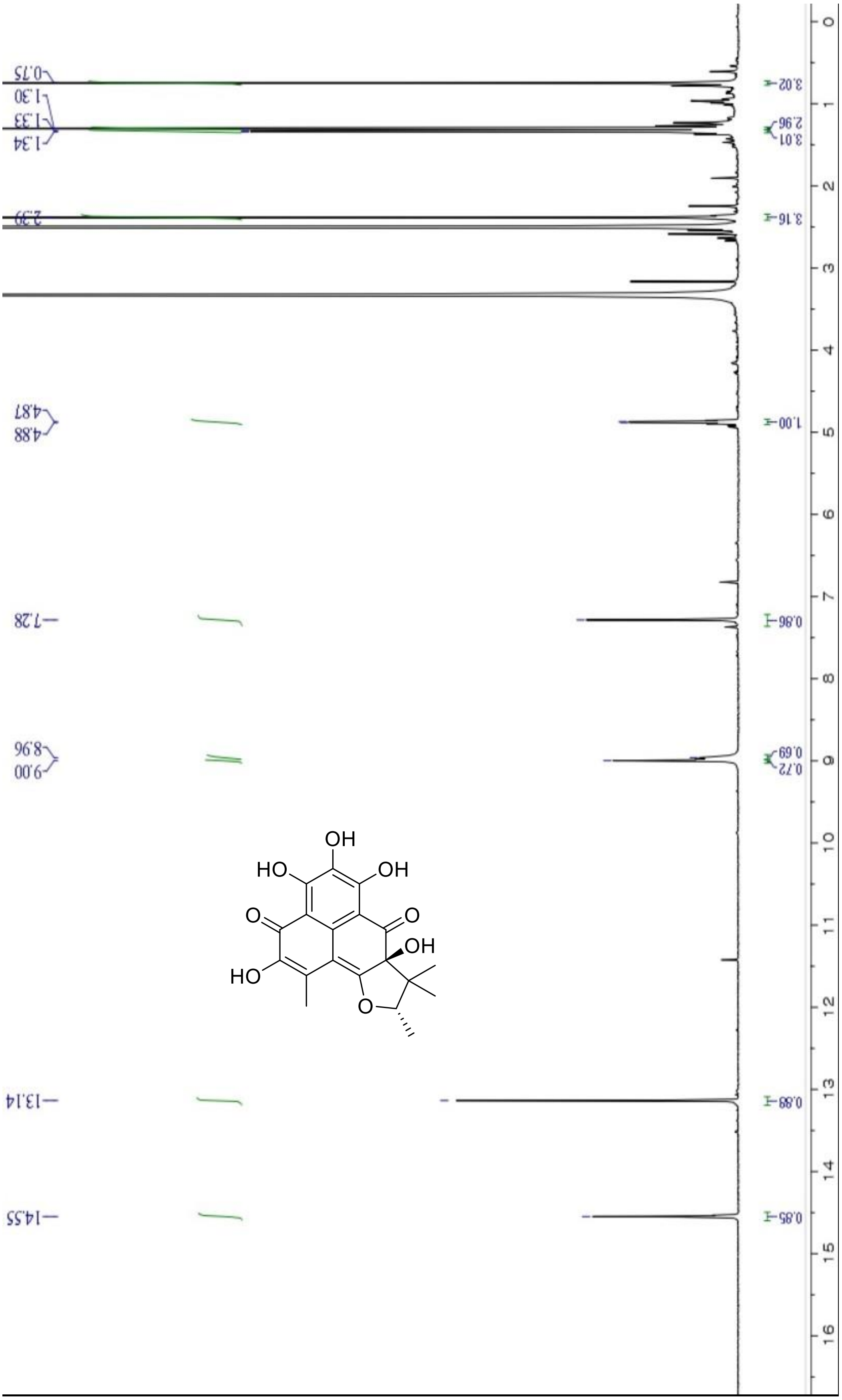

Figure S10. The ^{1}H NMR (600 MHz, DMSO-d_6) spectrum of **2**

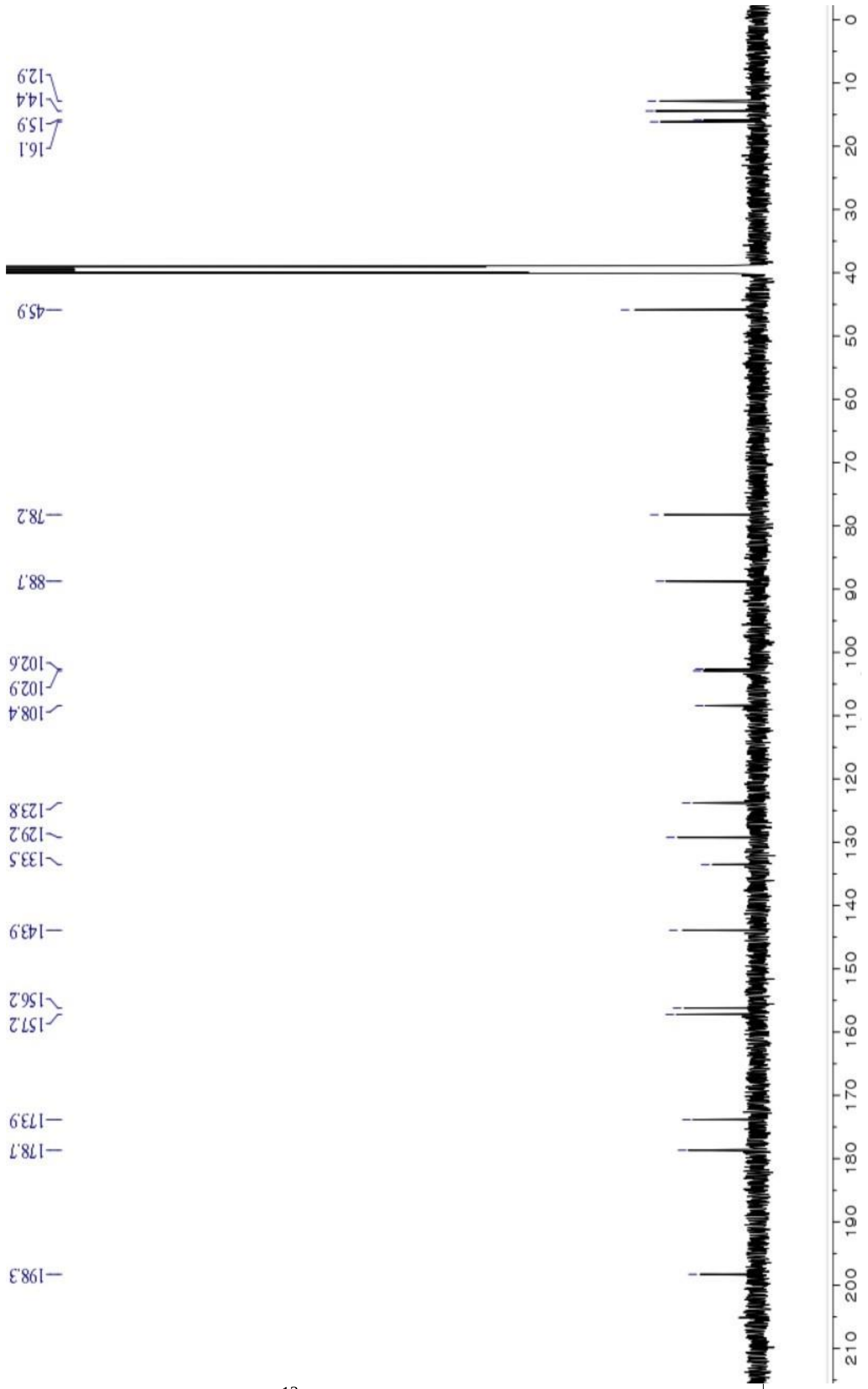

Figure S11. The ^{13}C NMR (150 MHz, DMSO-d_6) spectrum of **2**

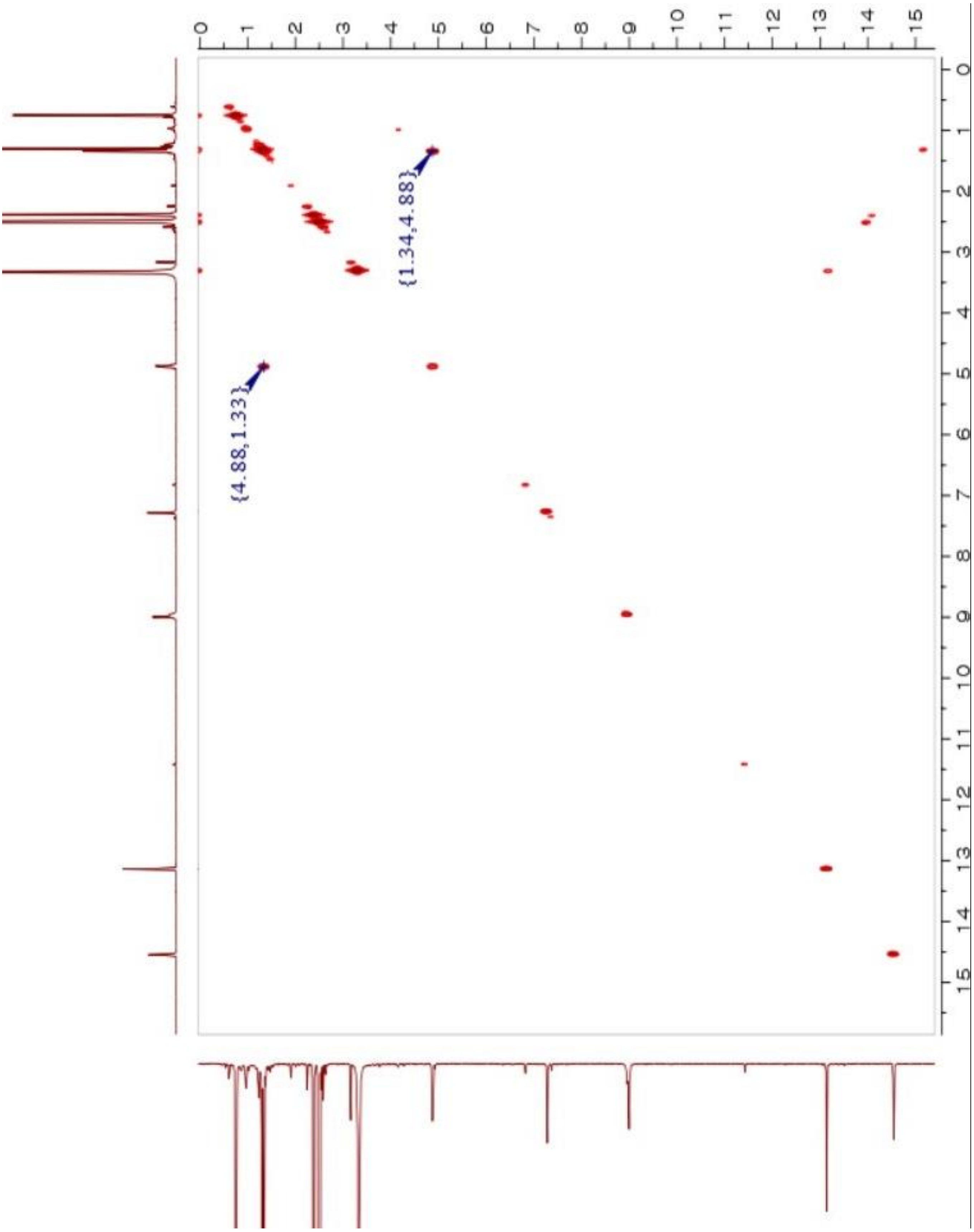

Figure S12. The COSY (600 MHz, DMSO-d_6) spectrum of **2**

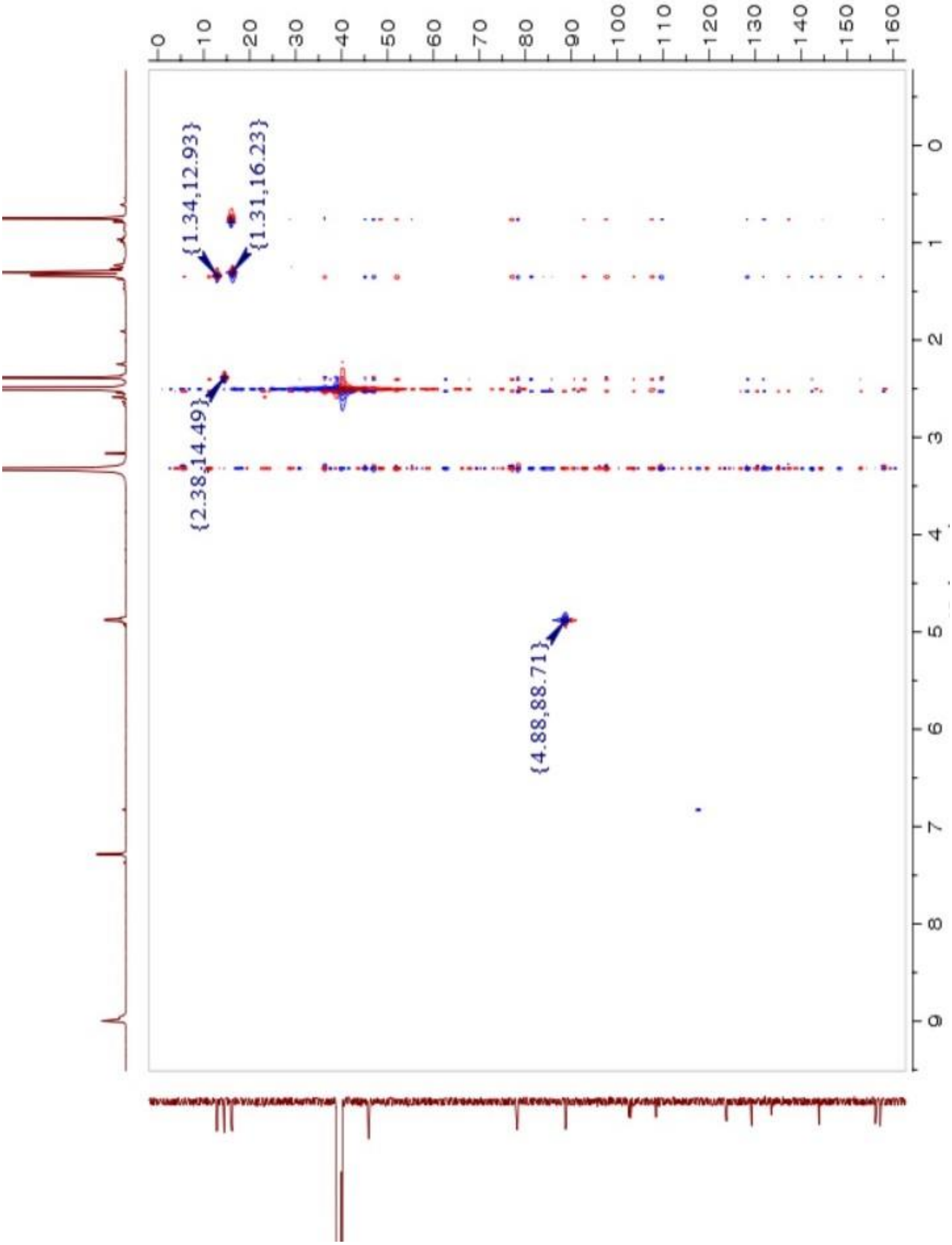

Figure S13. The HSQC (600 MHz, DMSO-d_6) spectrum of **2**

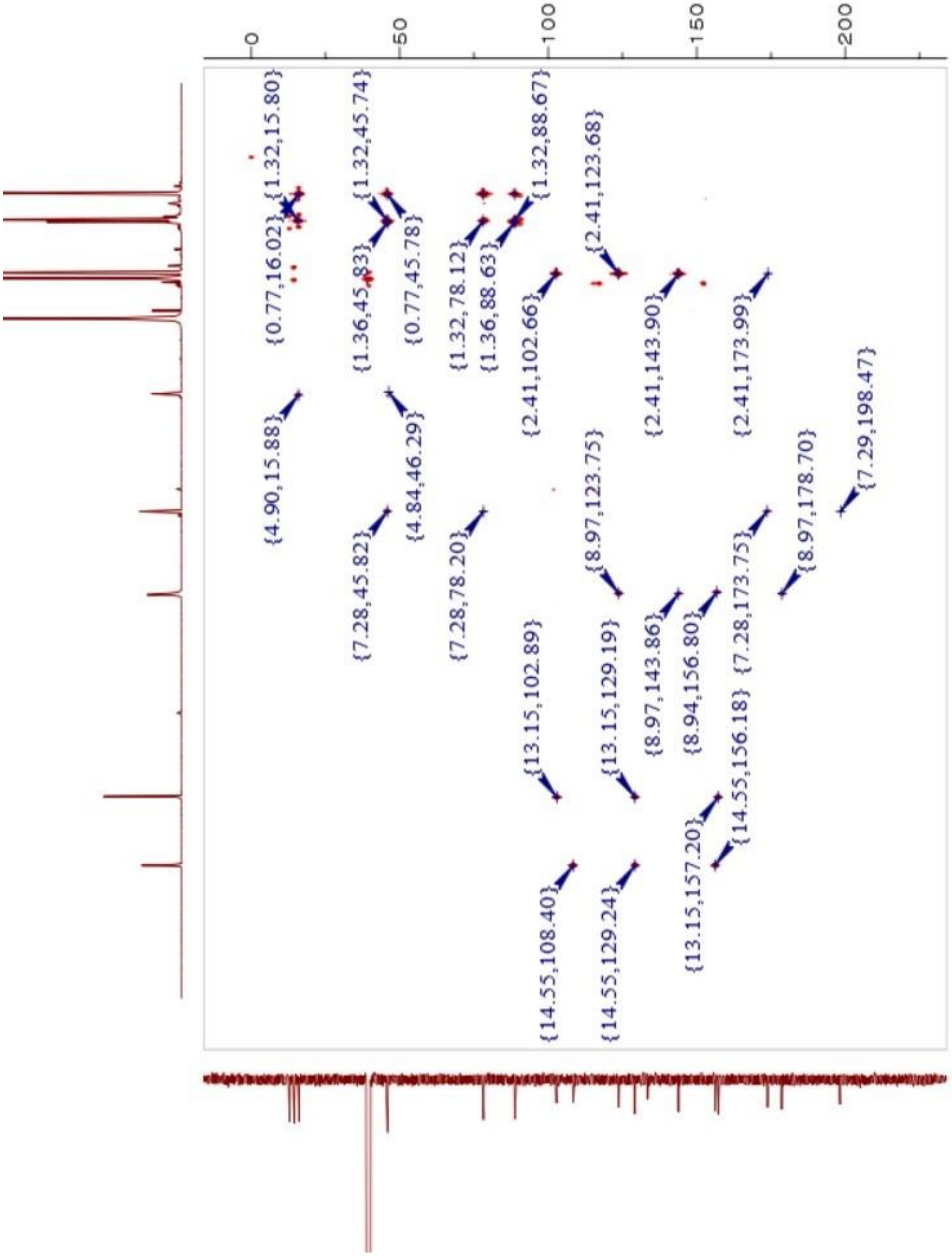

Figure S14. The HMBC (500 MHz, DMSO-d_6) spectrum of **2**

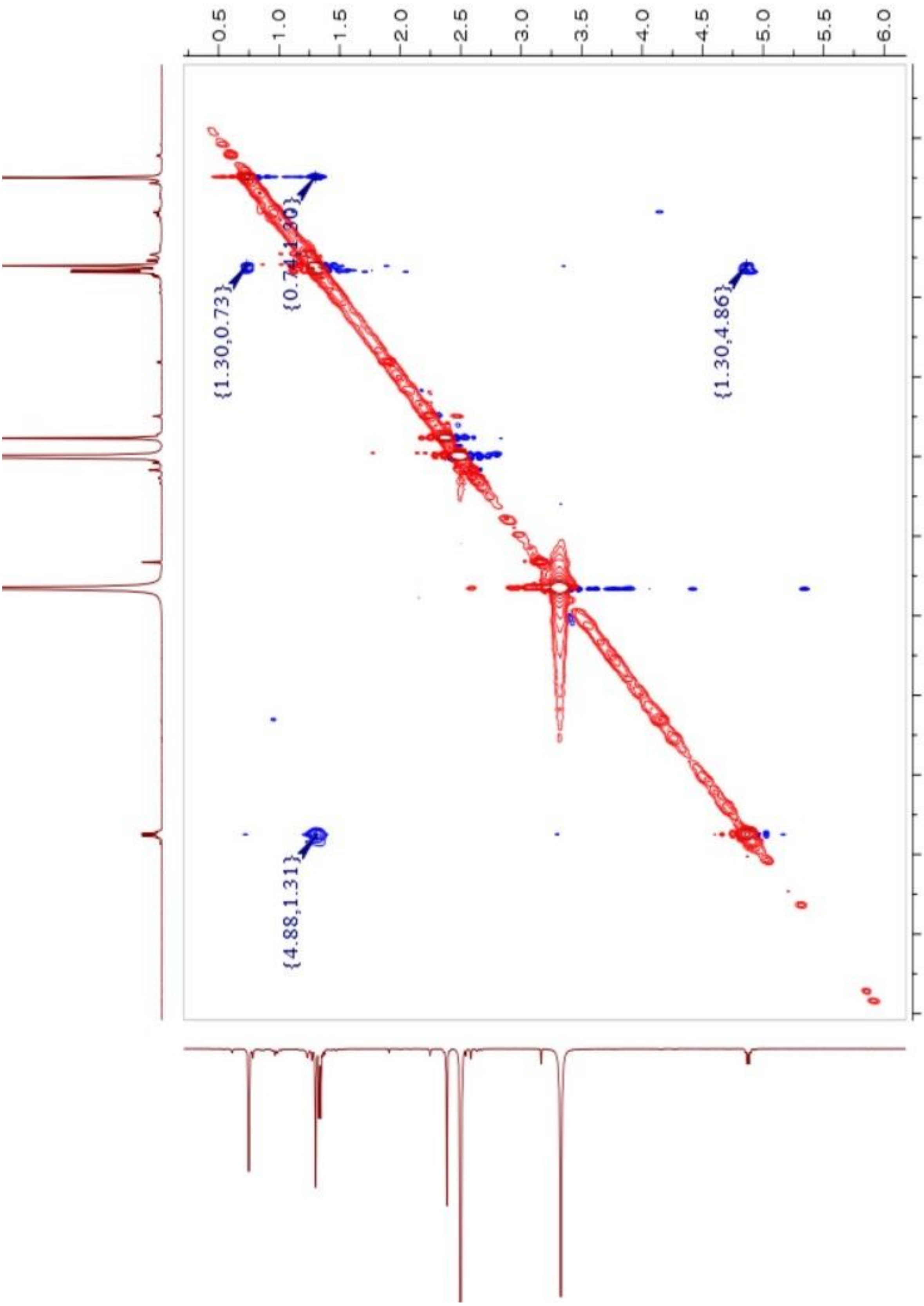

Figure S15. The NOESY (500 MHz, DMSO-d_6) spectrum of **2**

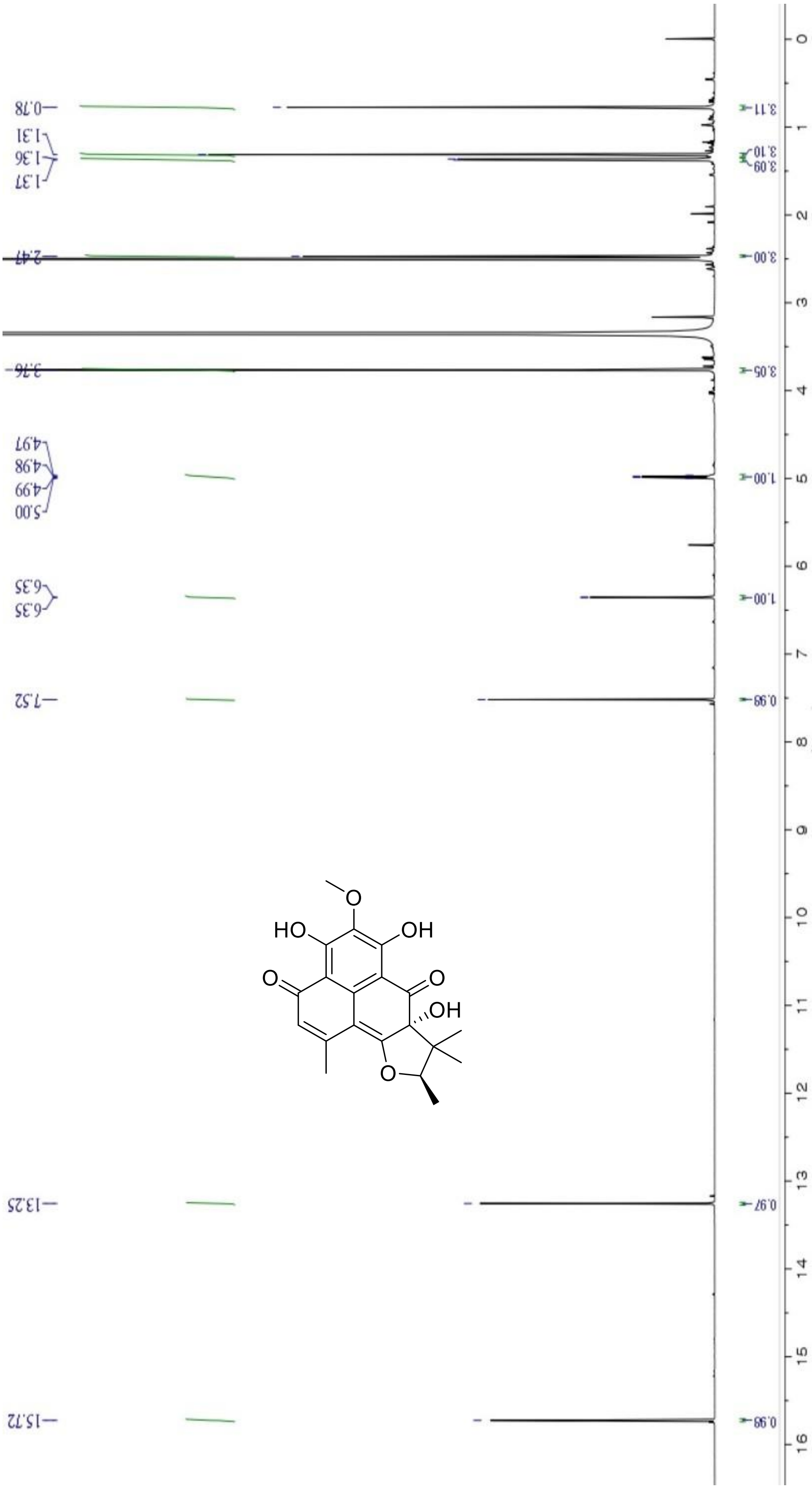

Figure S16. The ^{1}H NMR (600 MHz, DMSO-d_6) spectrum of **3**

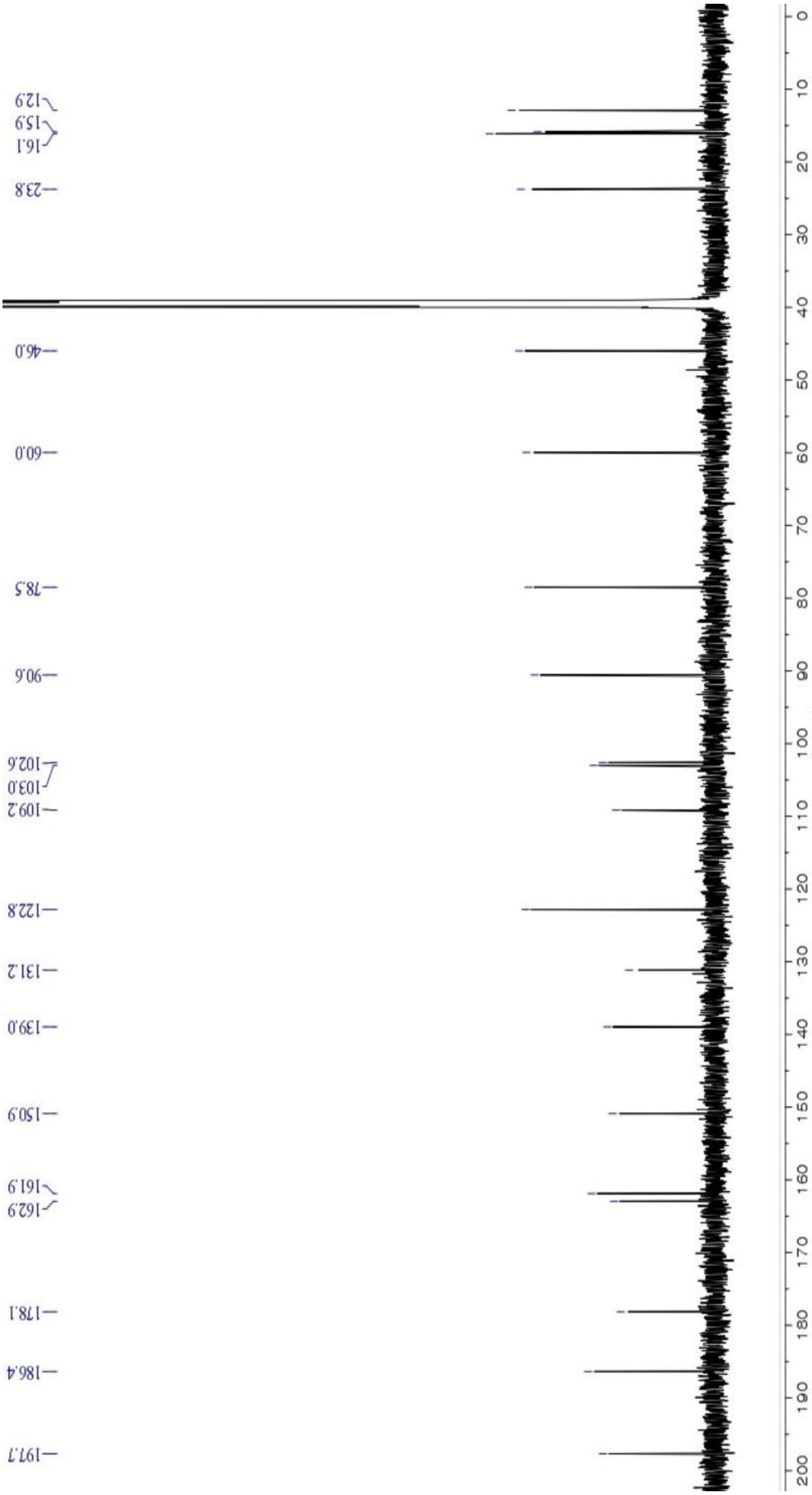

Figure S17. The ^{13}C NMR (150 MHz, DMSO-d_6) spectrum of **3**

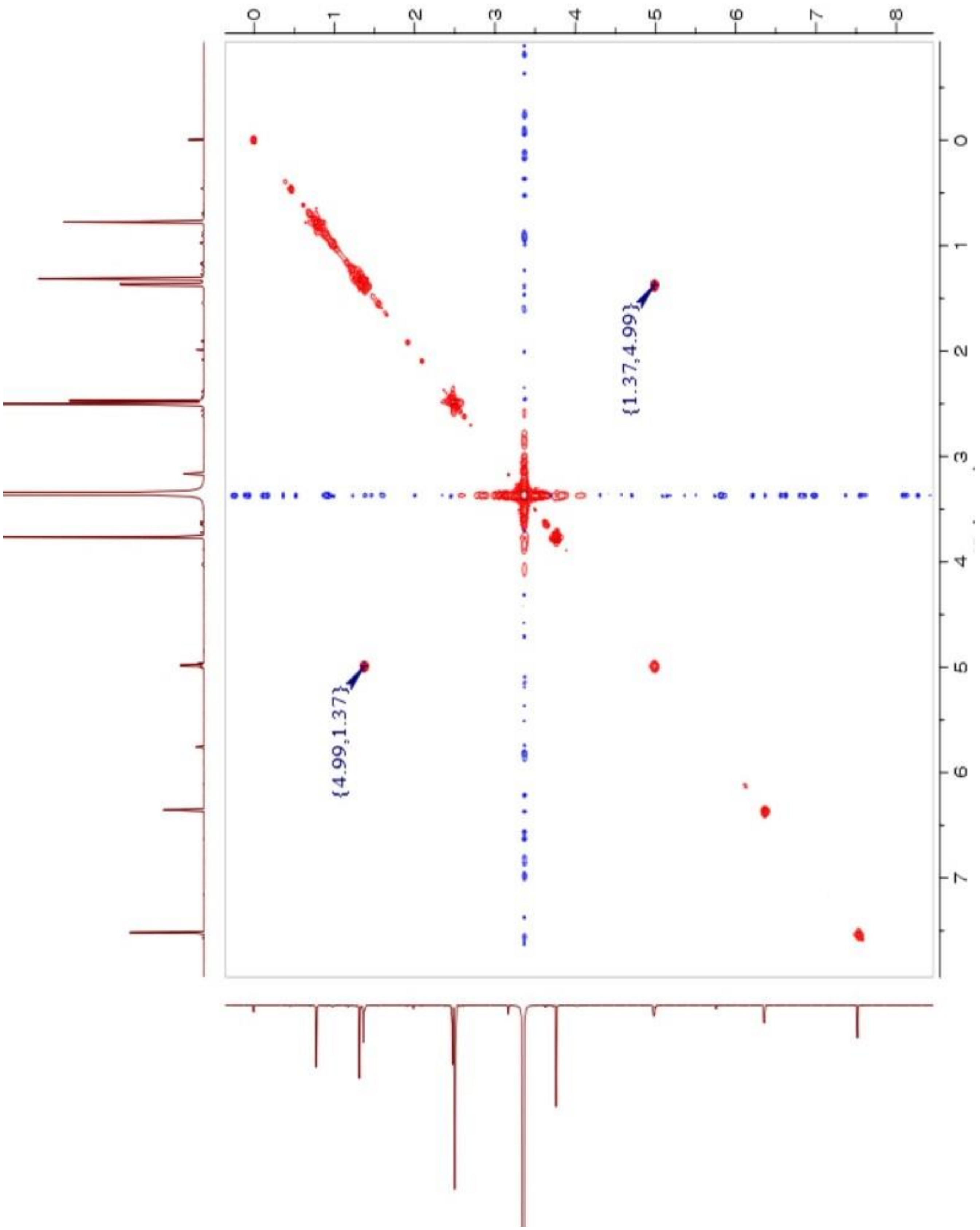

Figure S18. The COSY (600 MHz, DMSO-d_6) spectrum of **3**

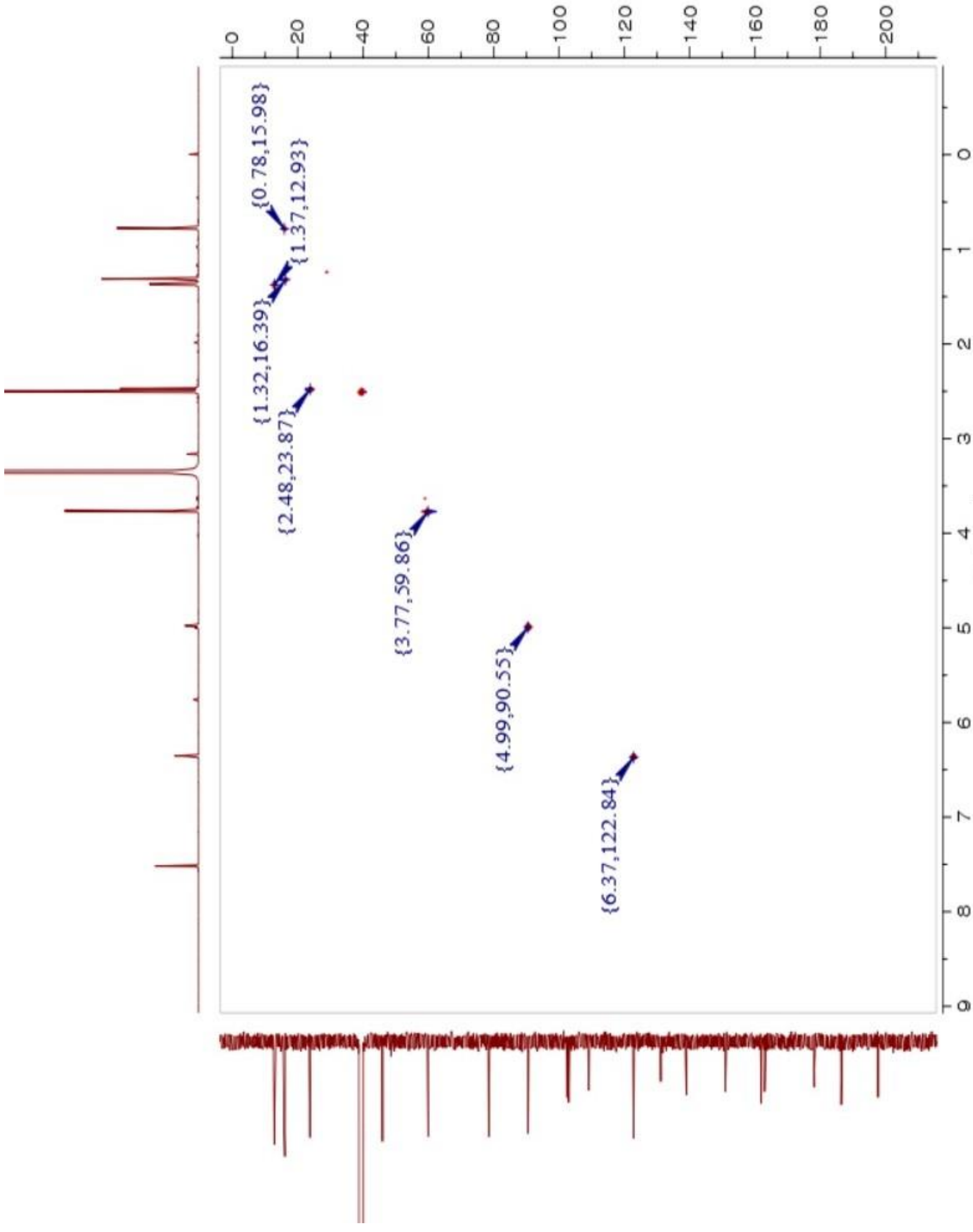

Figure S19. The HSQC (600 MHz, DMSO-d_6) spectrum of **3**

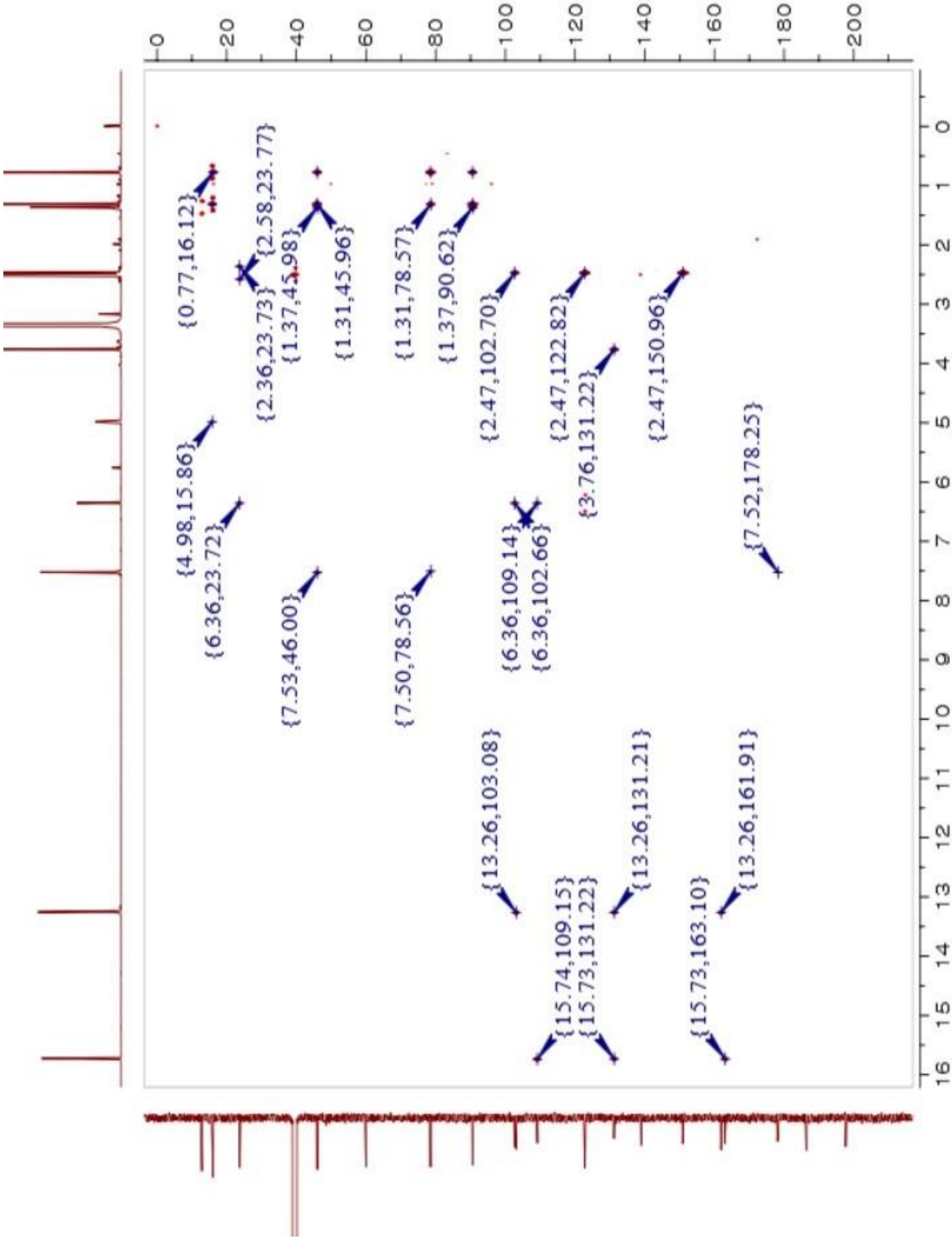

Figure S20. The HMBC (600 MHz, DMSO-d_6) spectrum of **3**

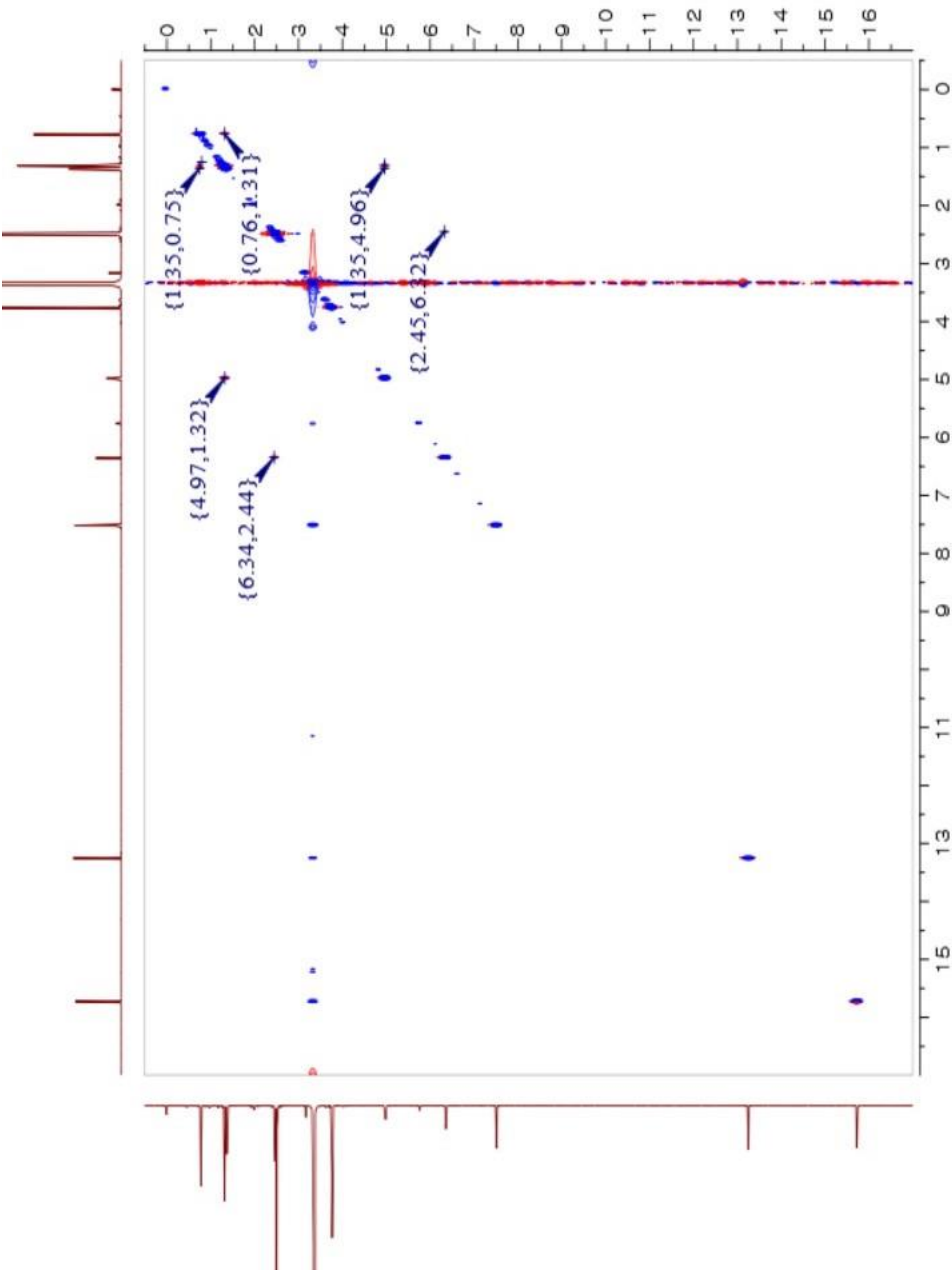

Figure S21. The NOESY (600 MHz, DMSO-d_6) spectrum of **3**

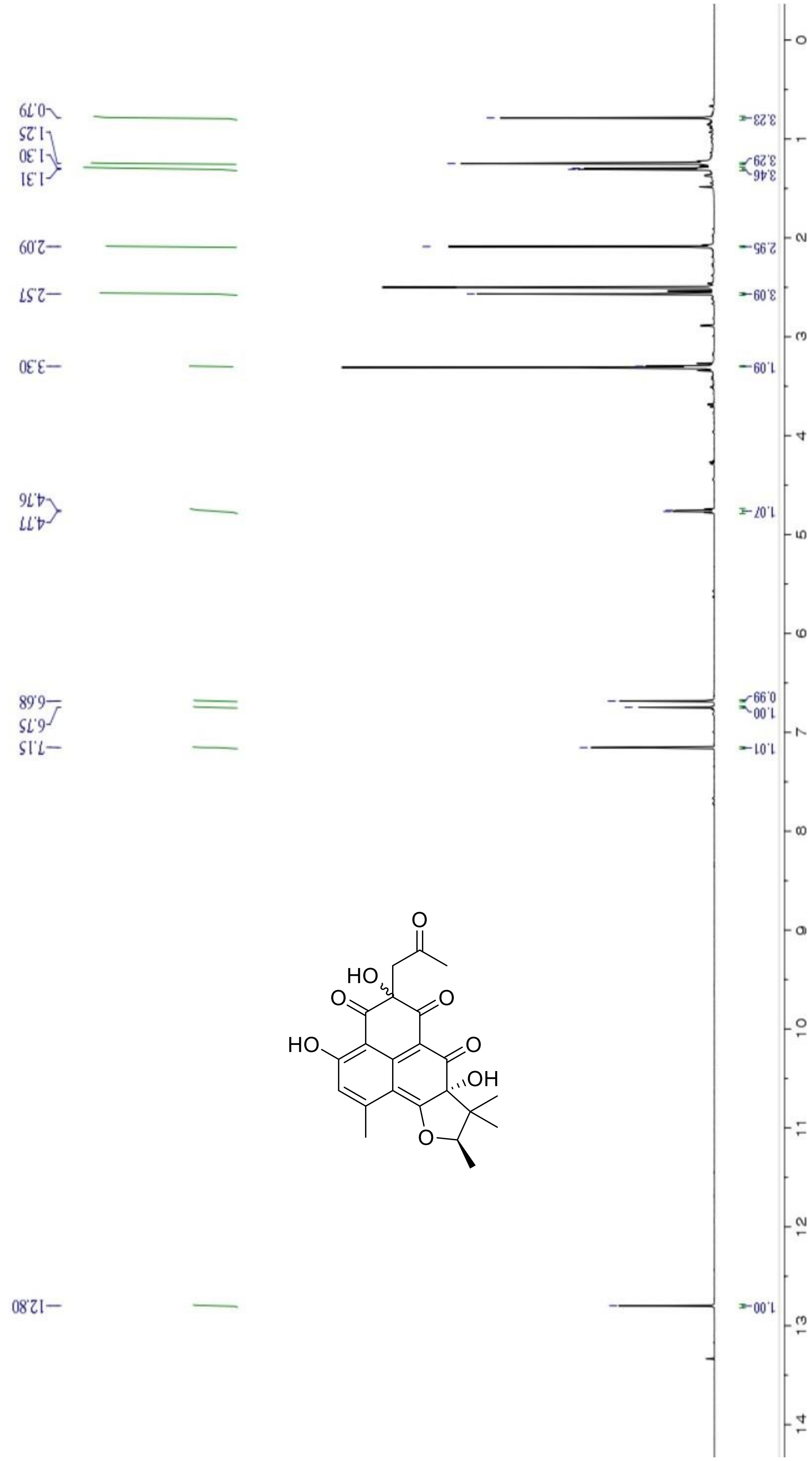

Figure S22. The ^{1}H NMR (600 MHz, DMSO-d_6) spectrum of **4**

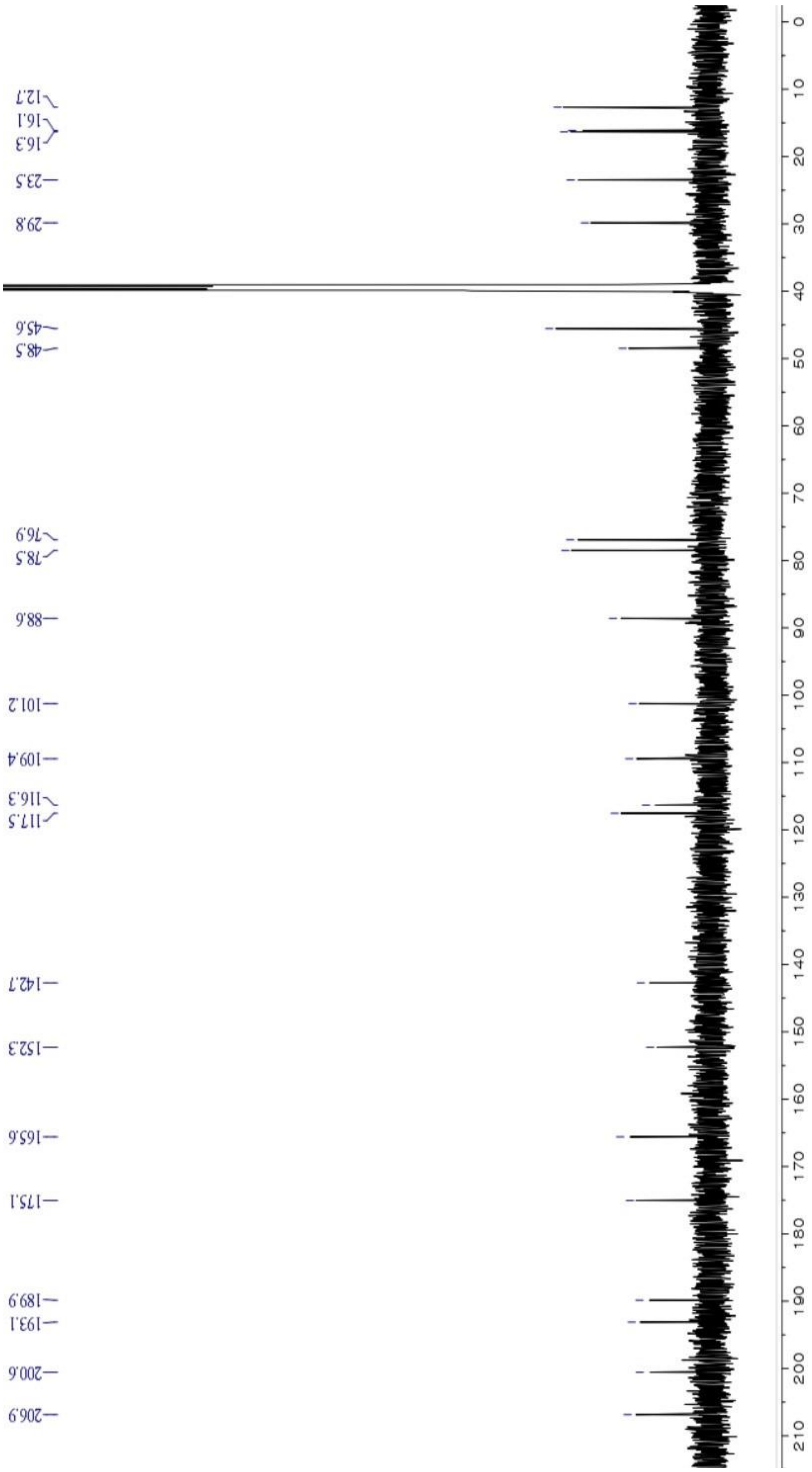

Figure S23. The ^{13}C NMR (150 MHz, DMSO-d_6) spectrum of **4**

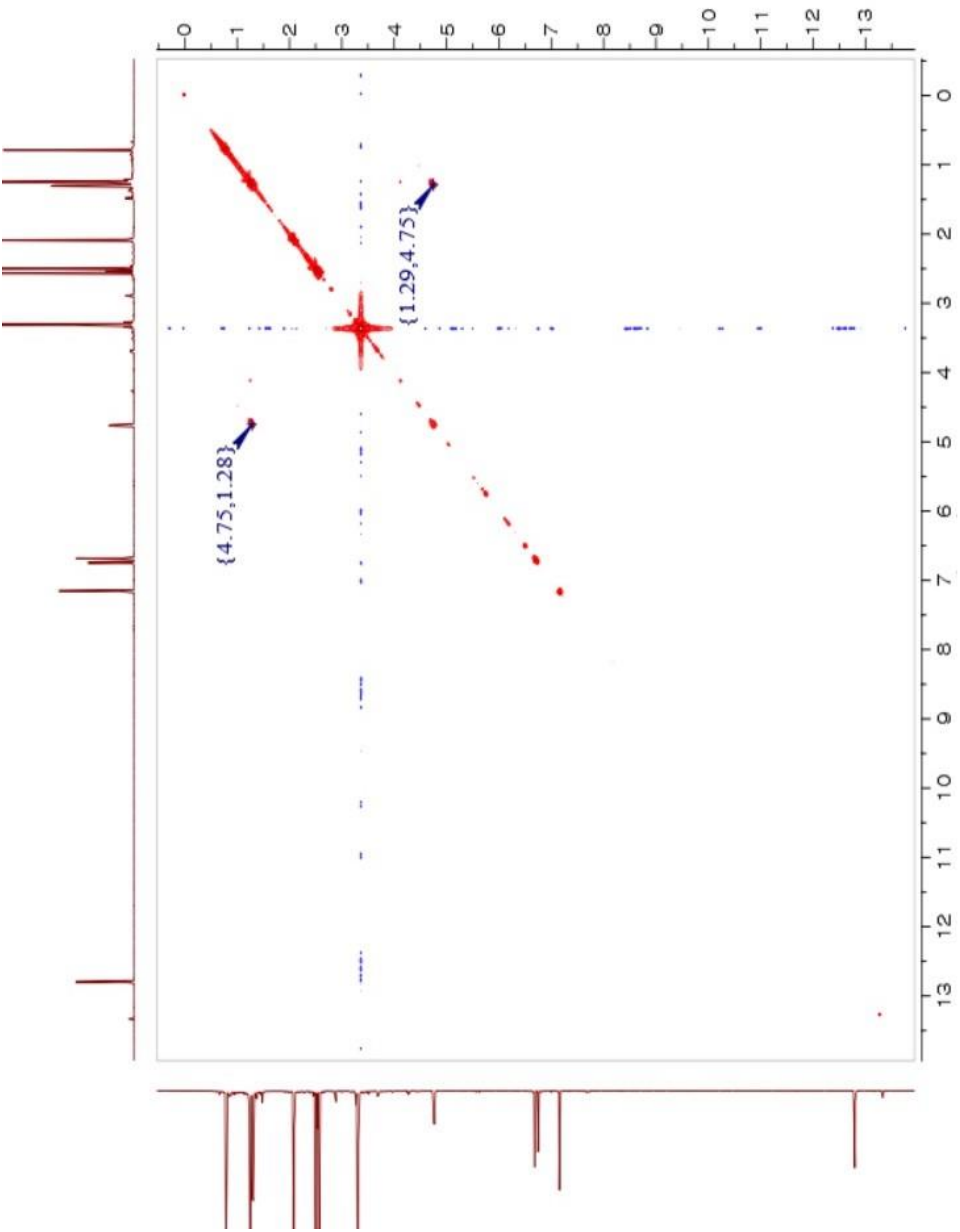

Figure S24. The COSY (600 MHz, DMSO-d_6) spectrum of **4**

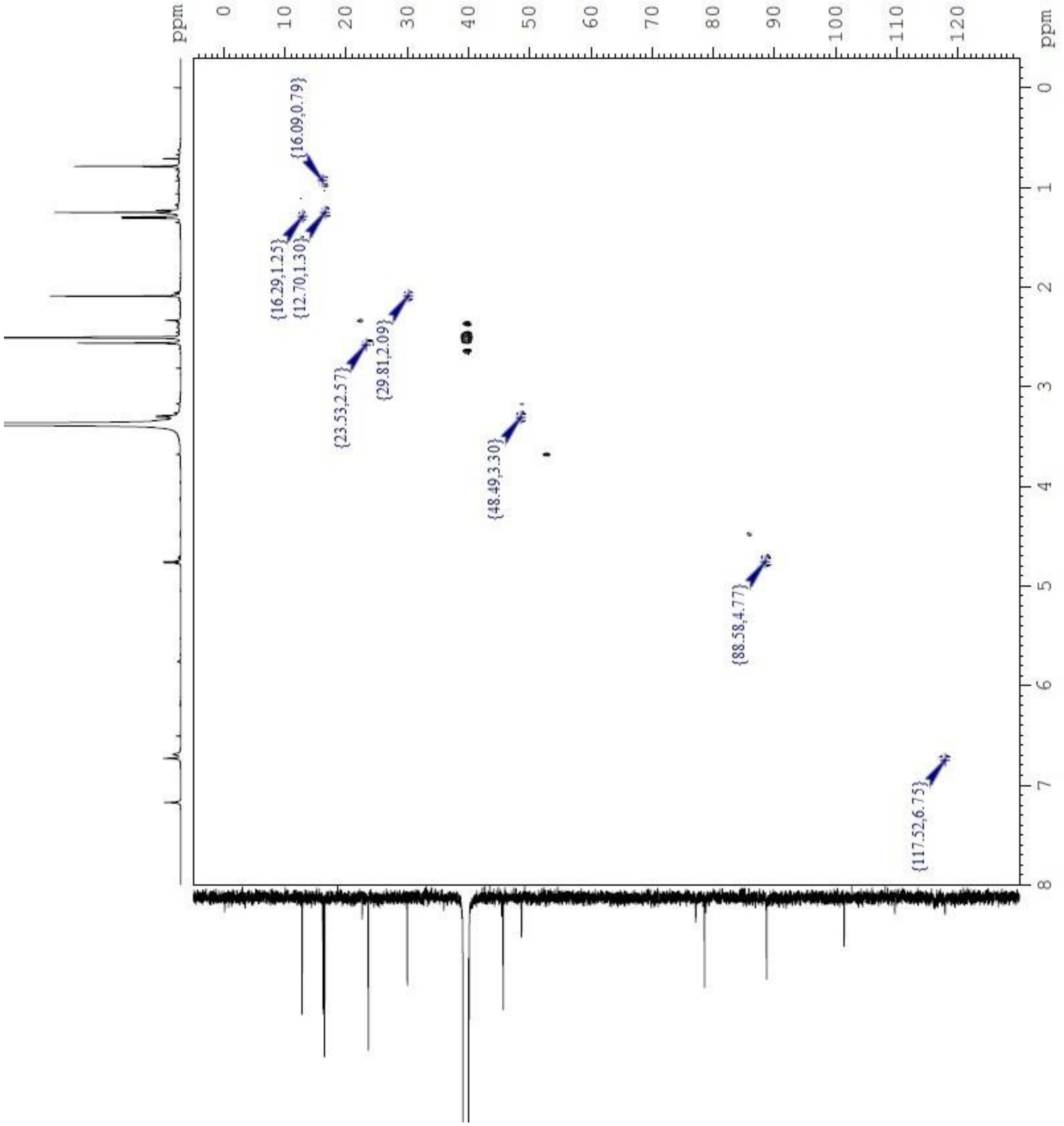

Figure S25. The HSQC (600 MHz, DMSO-d_6) spectrum of **4**

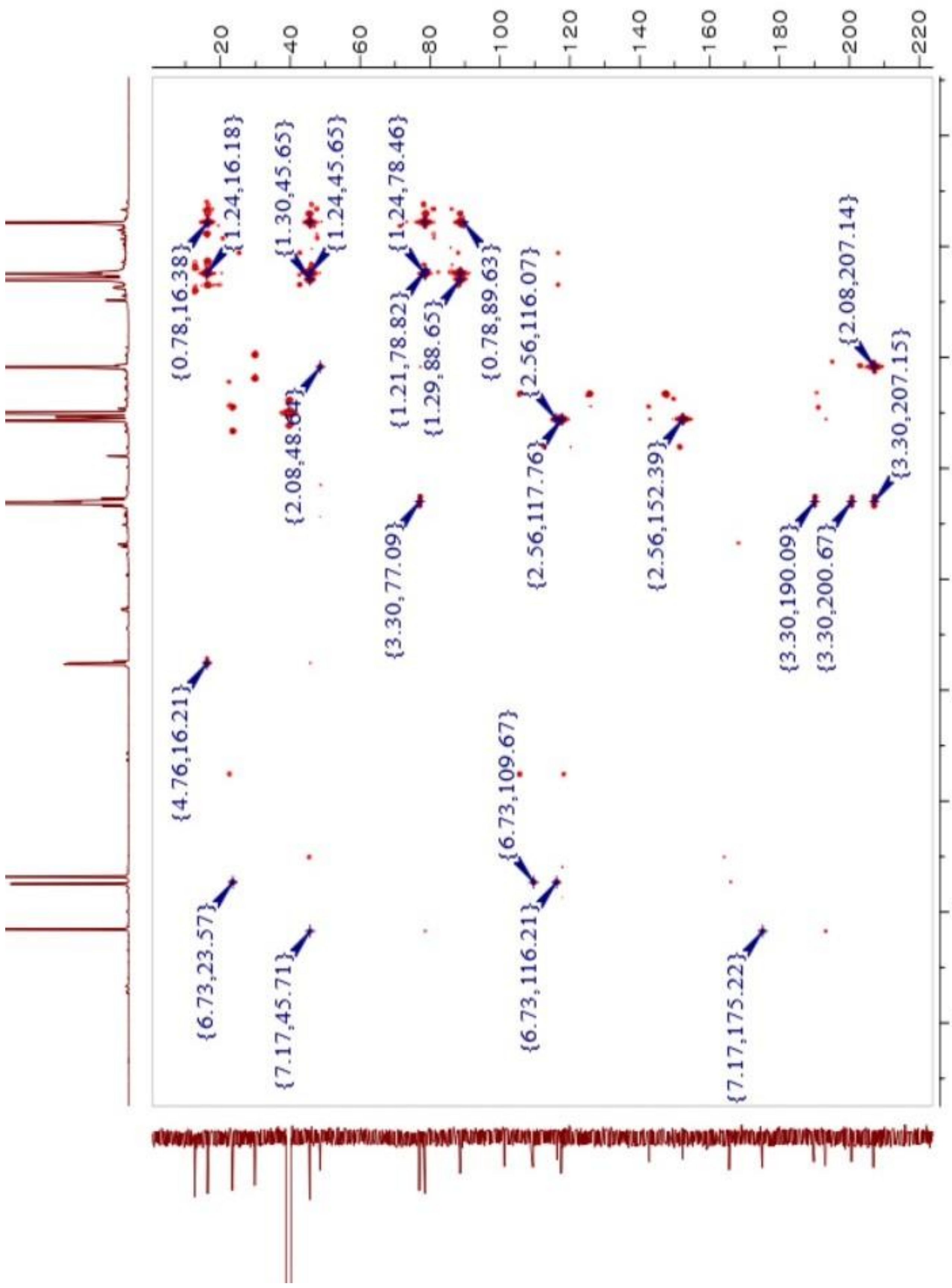

Figure S26. The HMBC (600 MHz, DMSO-d_6) spectrum of **4**

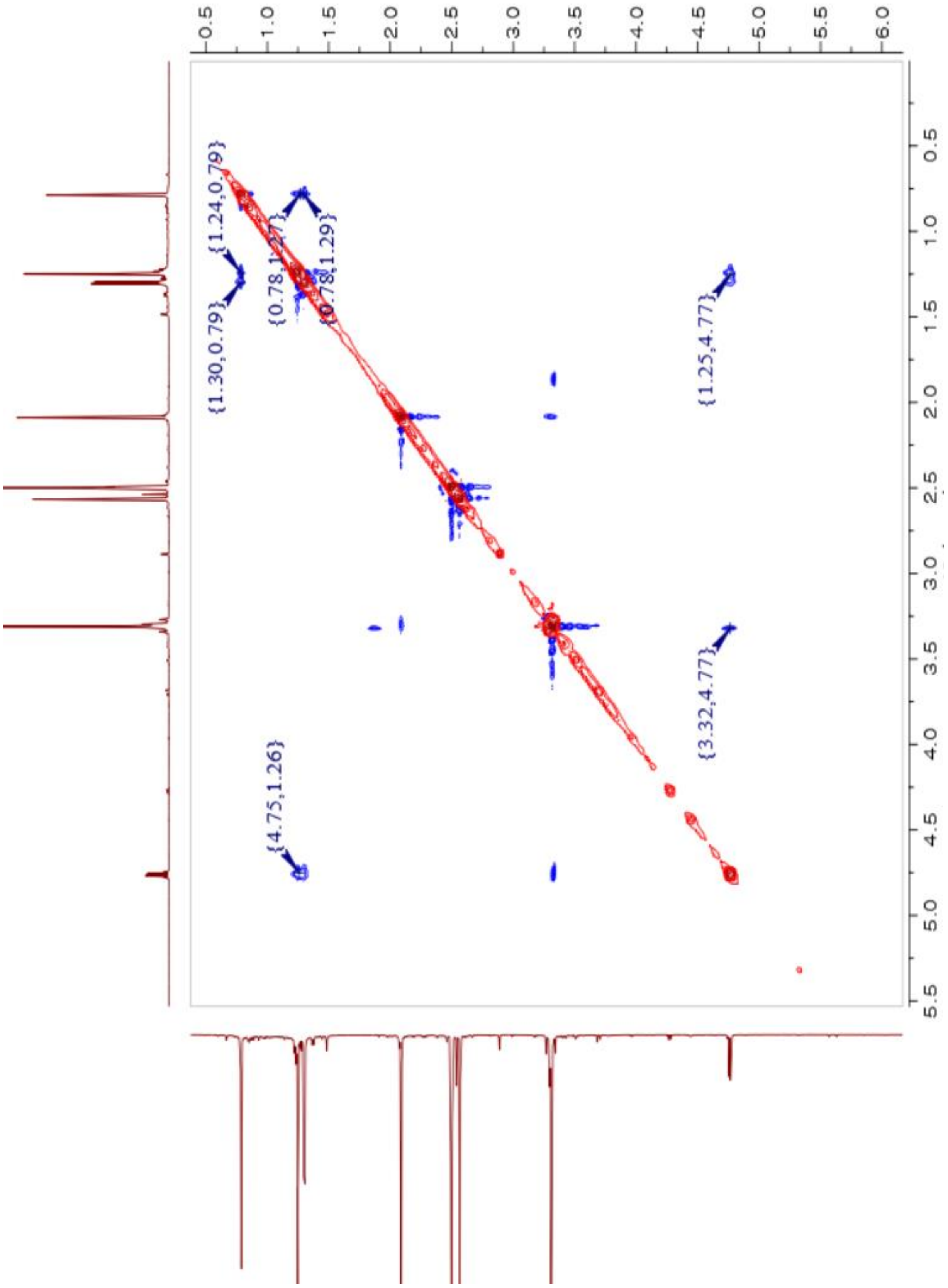

Figure S27. The NOESY (600 MHz, DMSO-d_6) spectrum of **4**

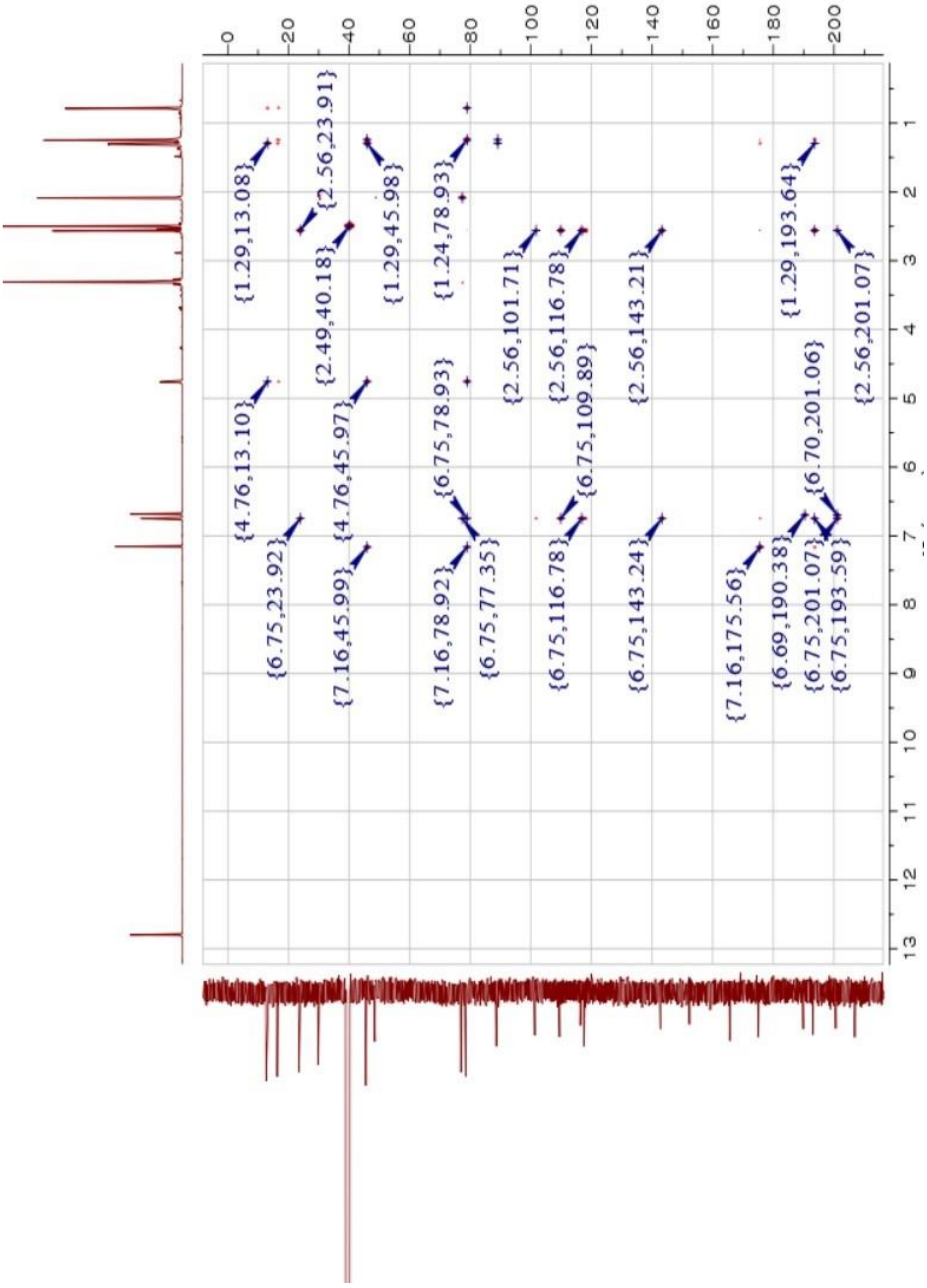

Figure S28. The D-HMBC (800 MHz, J_{CH} = 1 Hz, DMSO-d_6) spectrum of **4**

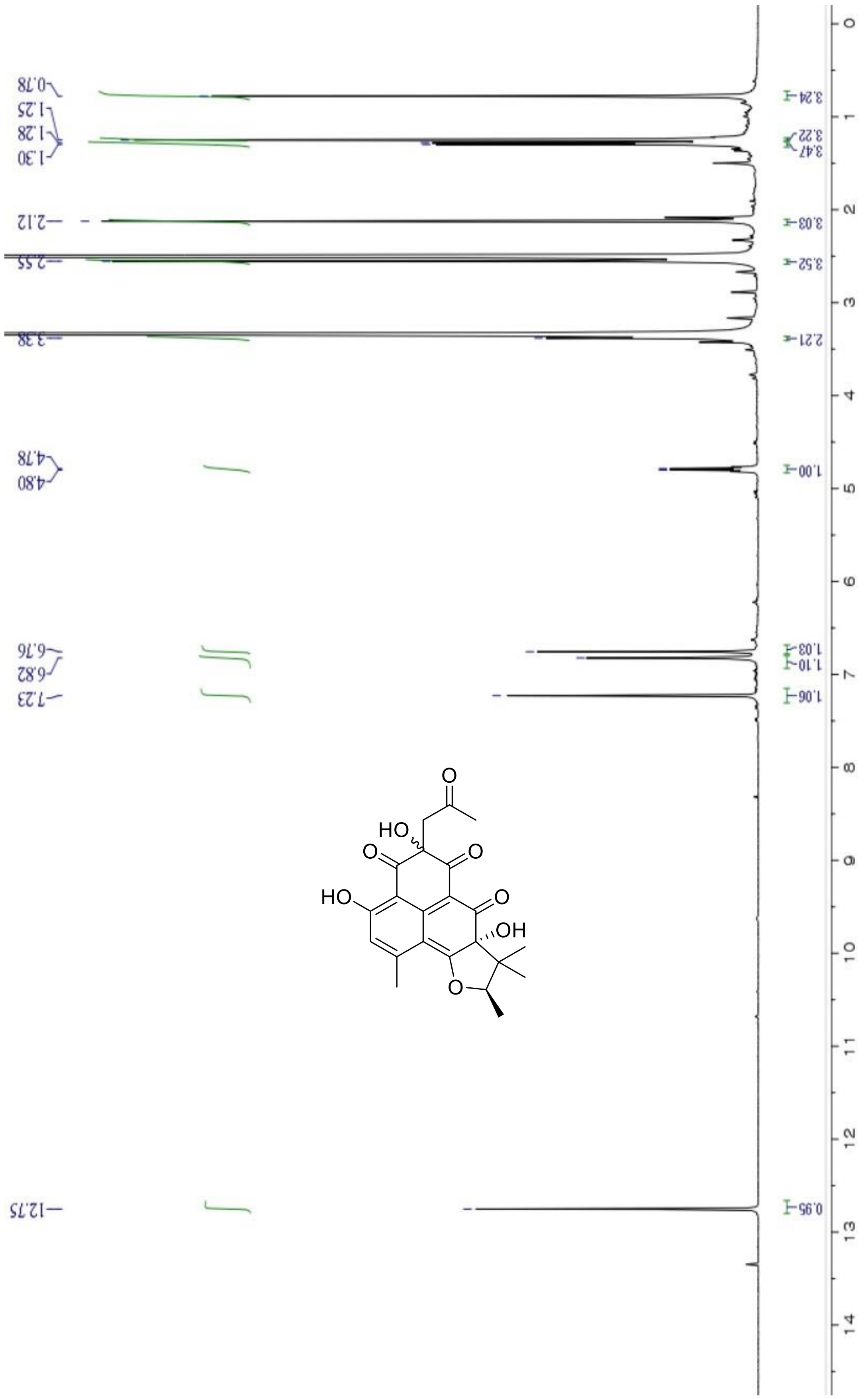

Figure S29. The ^{1}H NMR (600 MHz, DMSO-d_6) spectrum of **5**

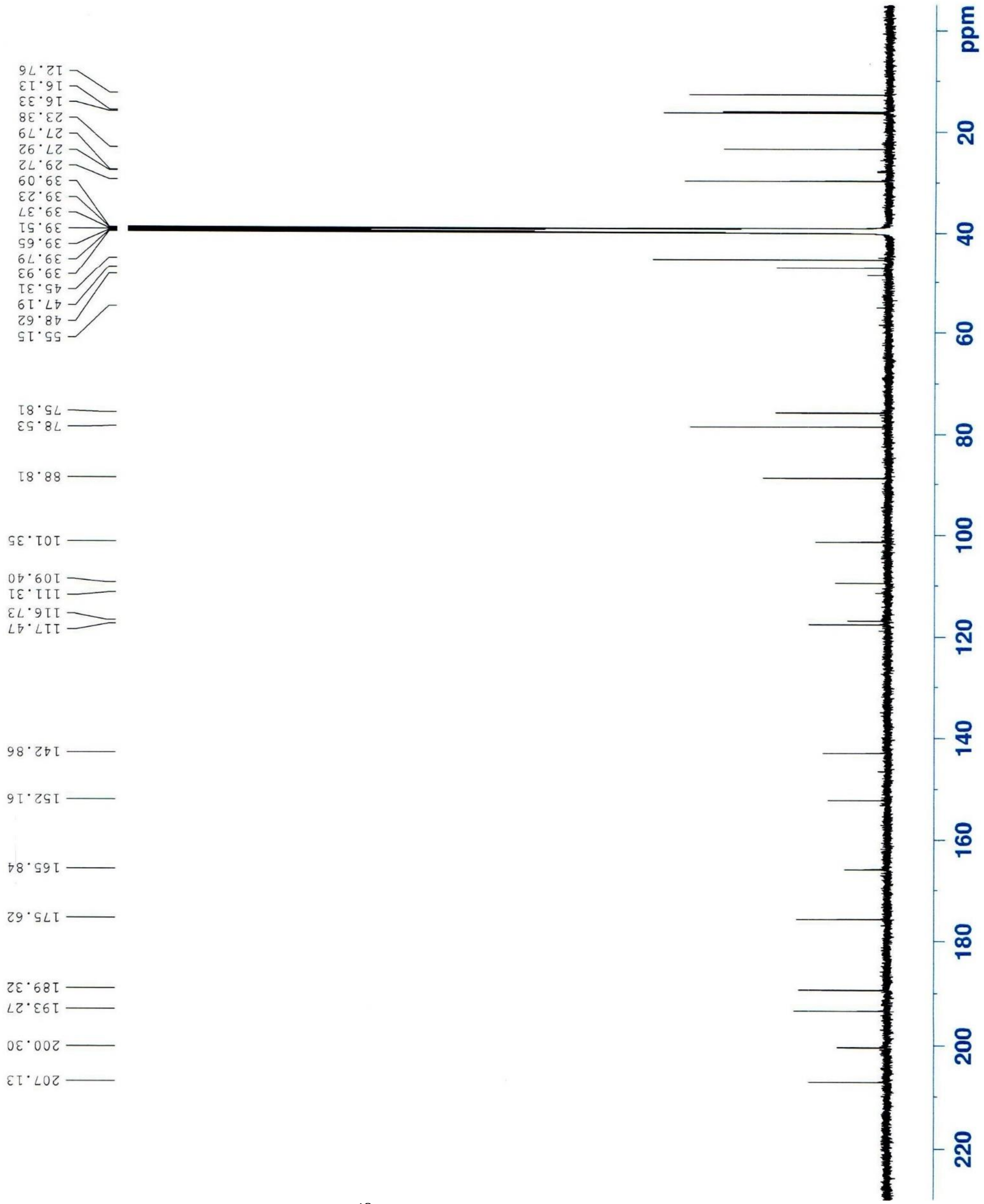

Figure S30. The ^{13}C NMR (150 MHz, DMSO-d_6) spectrum of **5**

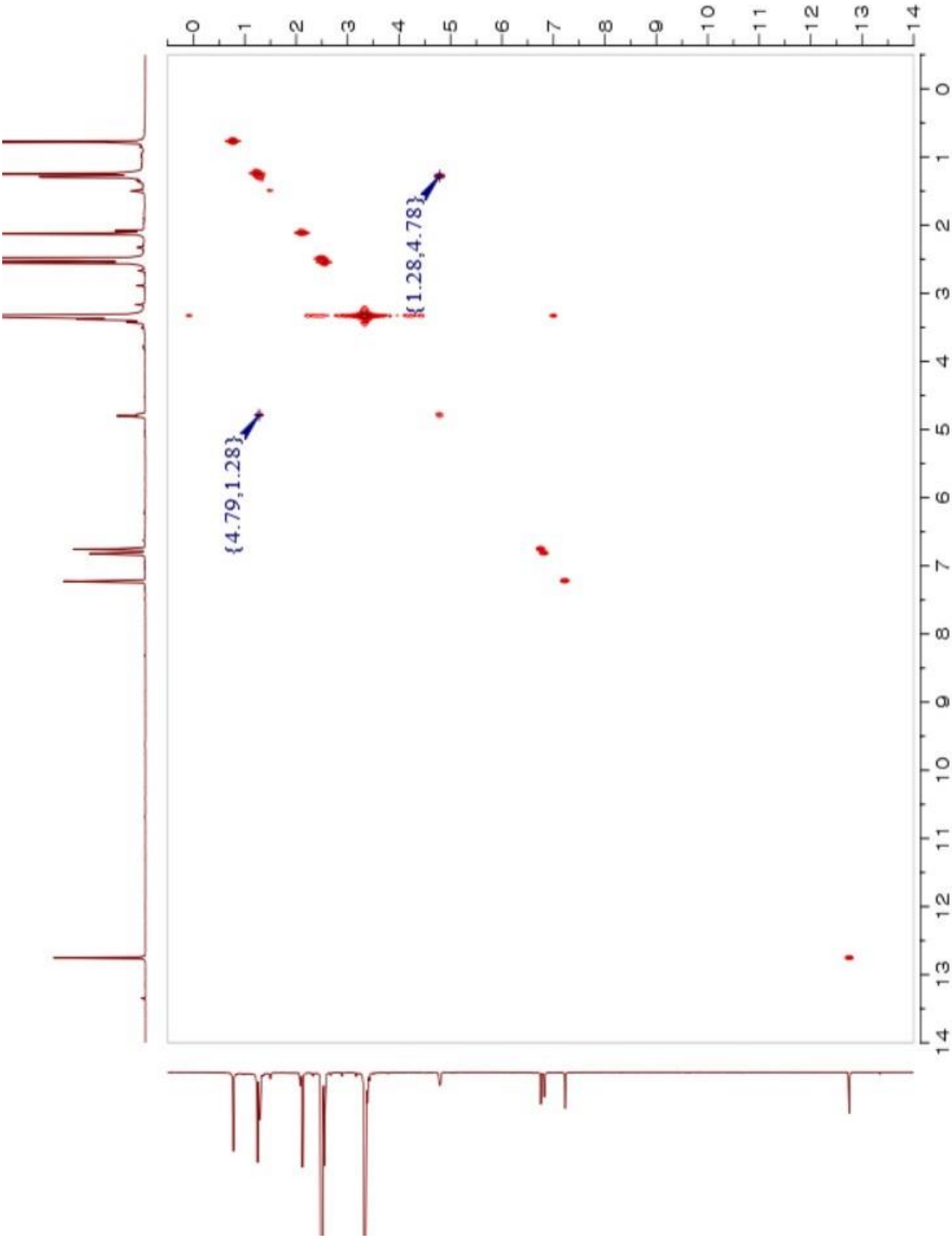

Figure S31. The COSY (600 MHz, DMSO-d_6) spectrum of **5**

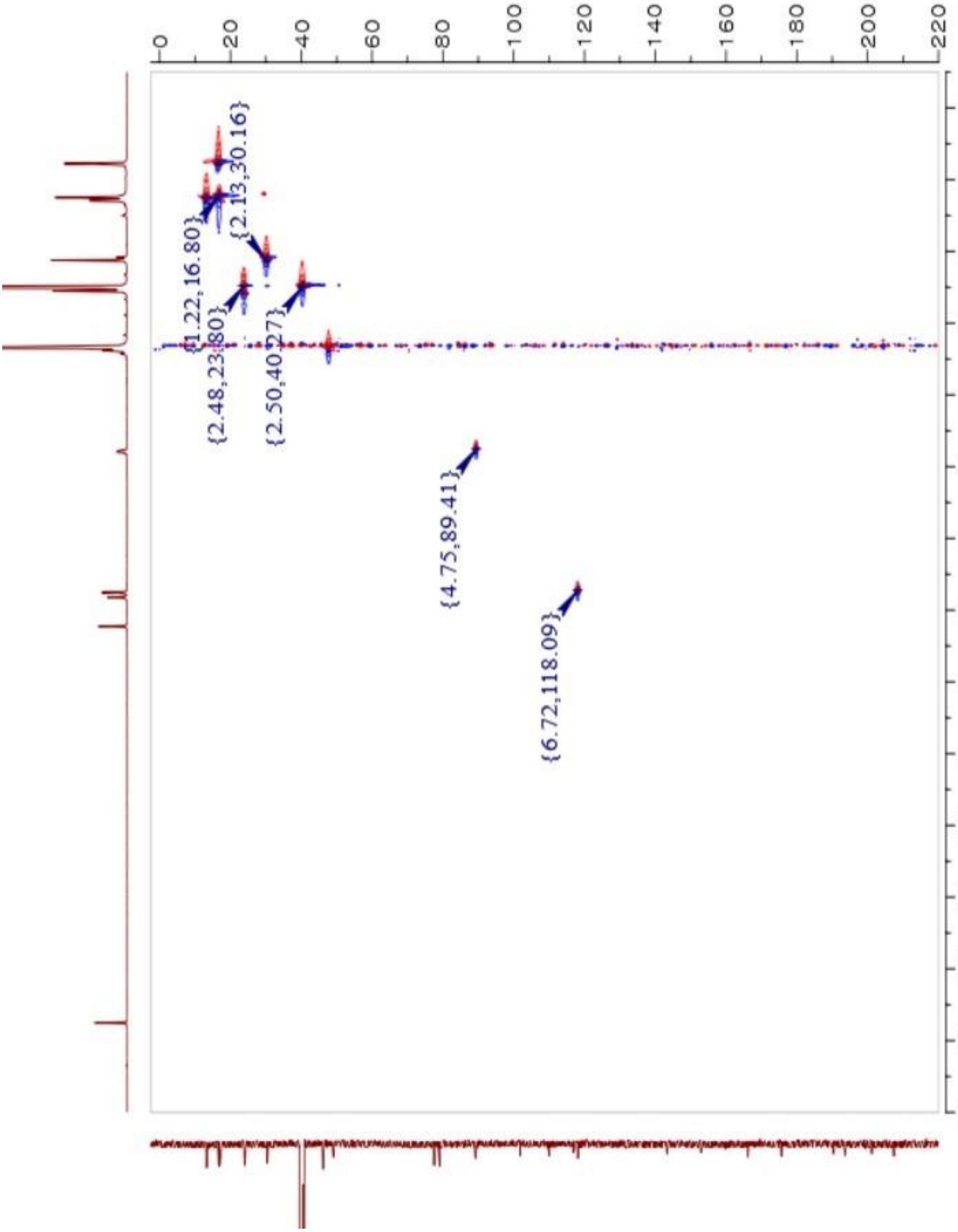

Figure S32. The HSQC (600 MHz, DMSO-d_6) spectrum of **5**

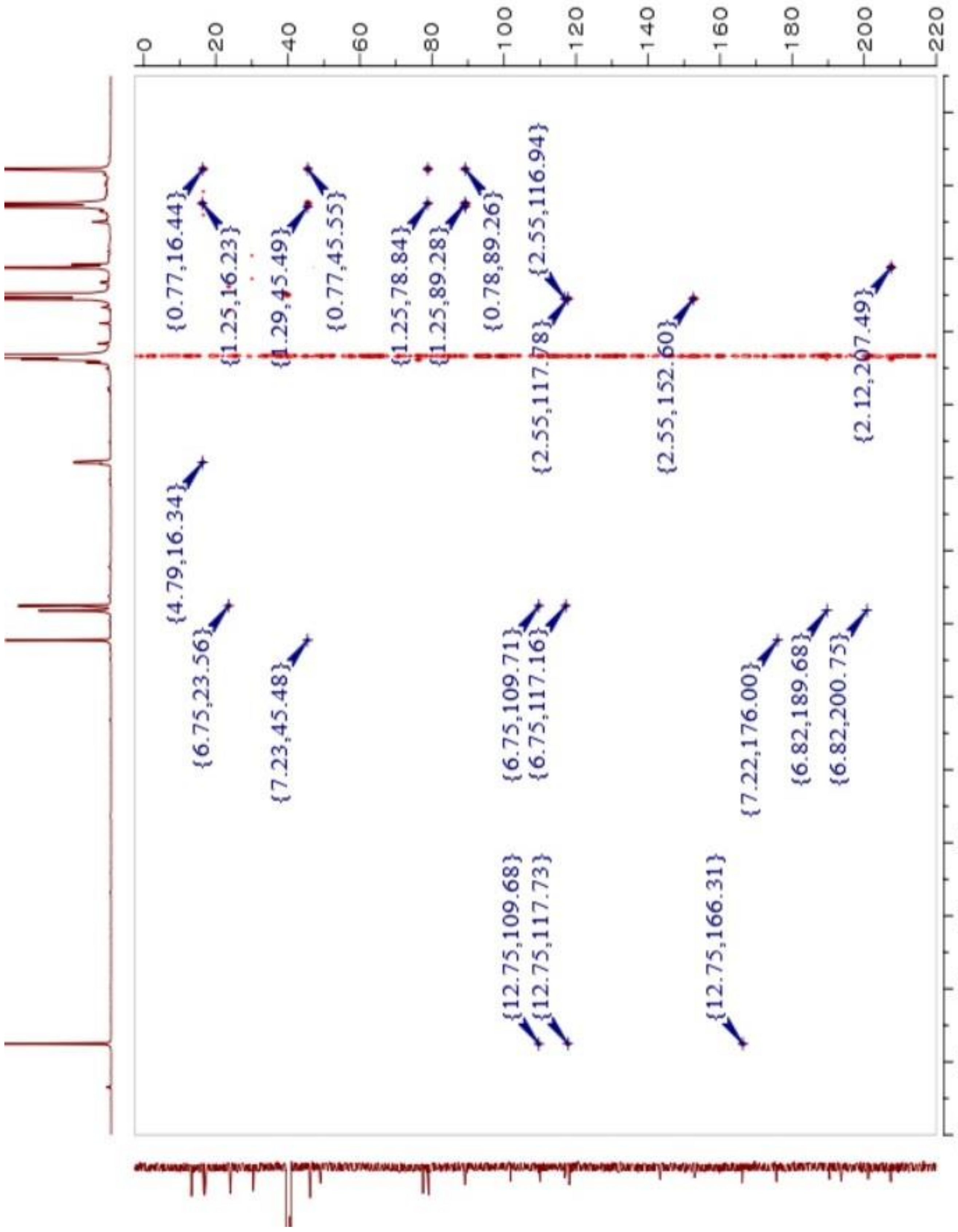

Figure S33. The HMBC (600 MHz, DMSO-d_6) spectrum of **5**

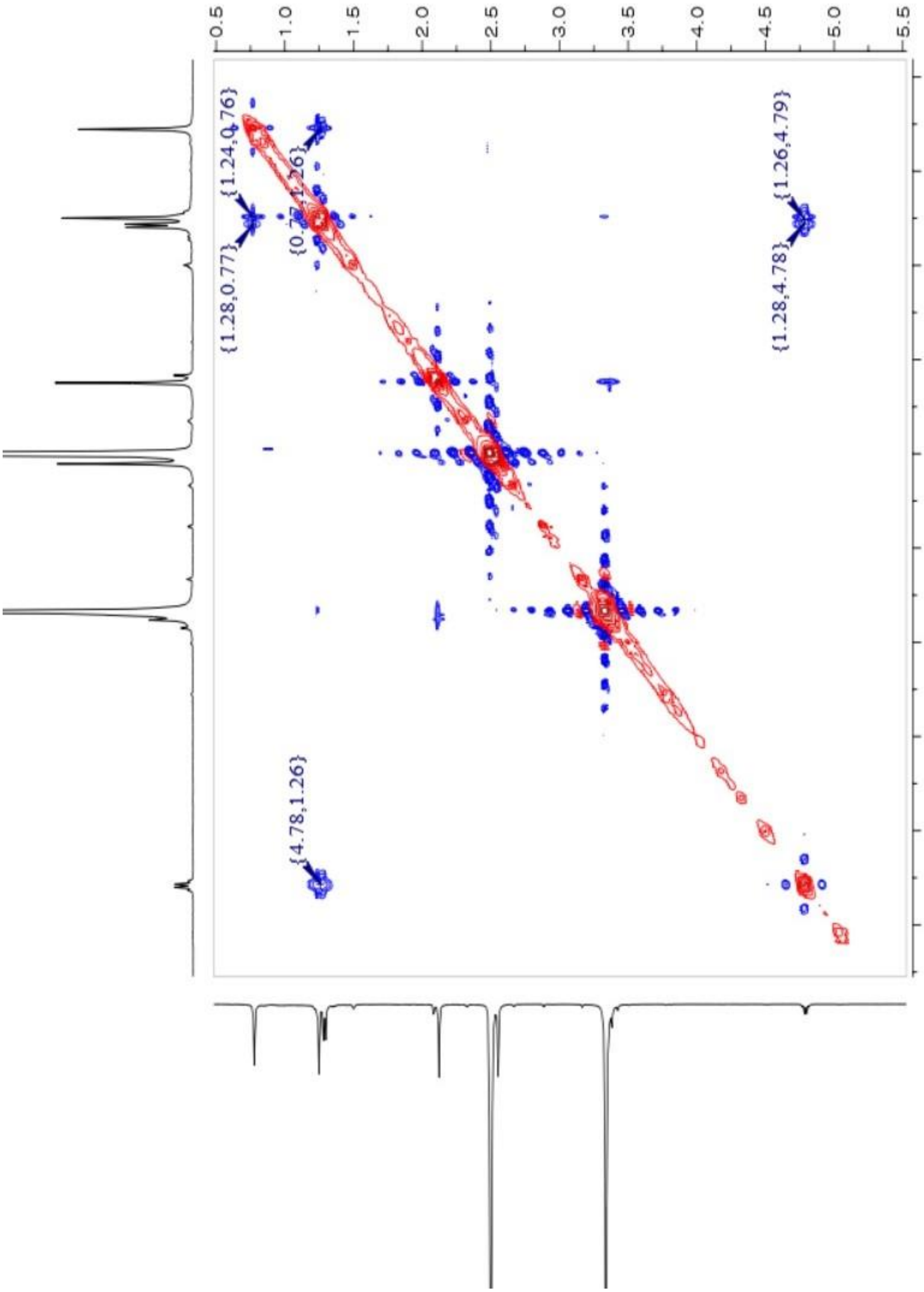

Figure S34. The NOESY (400 MHz, DMSO-d_6) spectrum of **5**

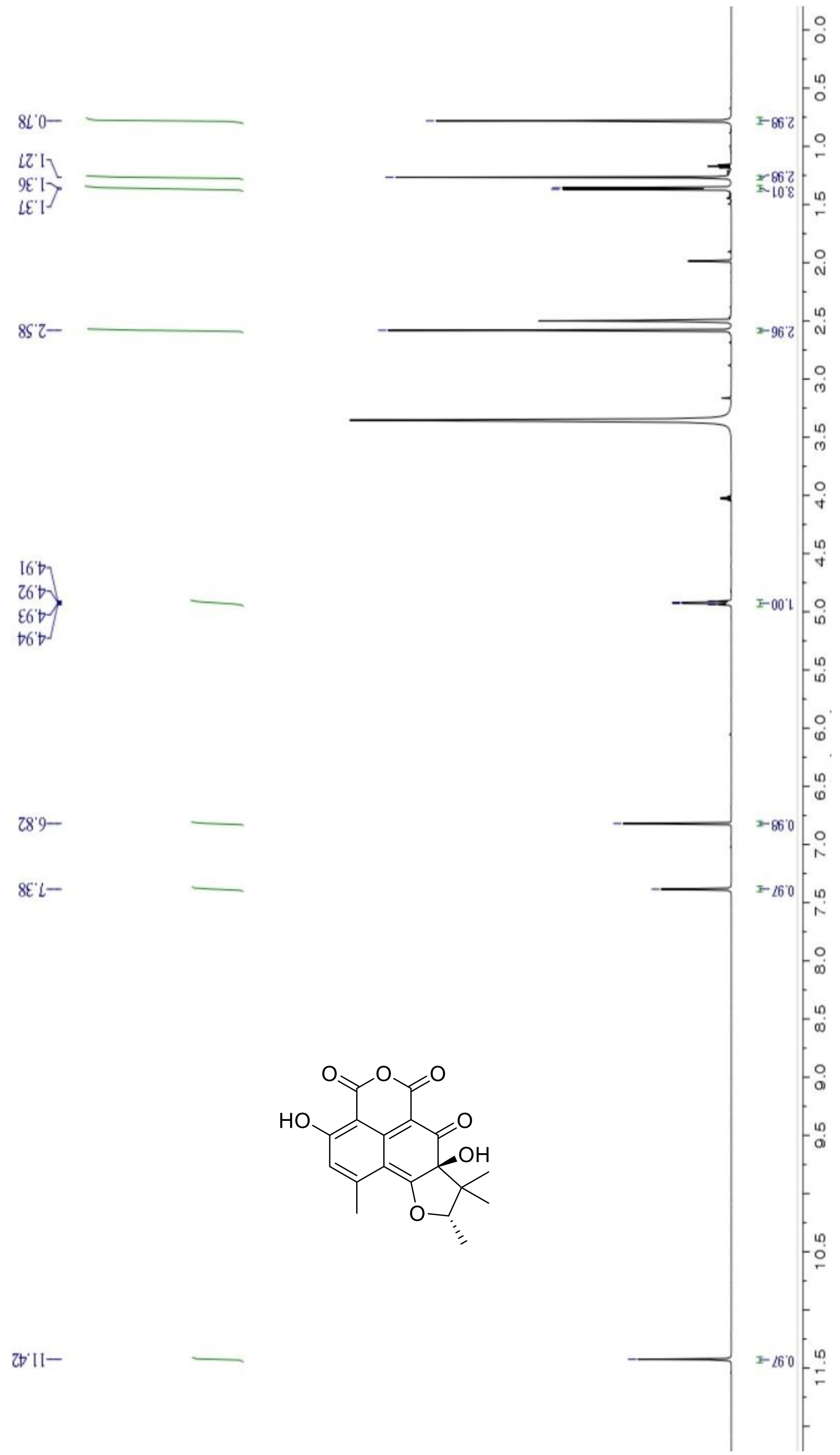

Figure S35. The ^{1}H NMR (600 MHz, DMSO-d_6) spectrum of **6**

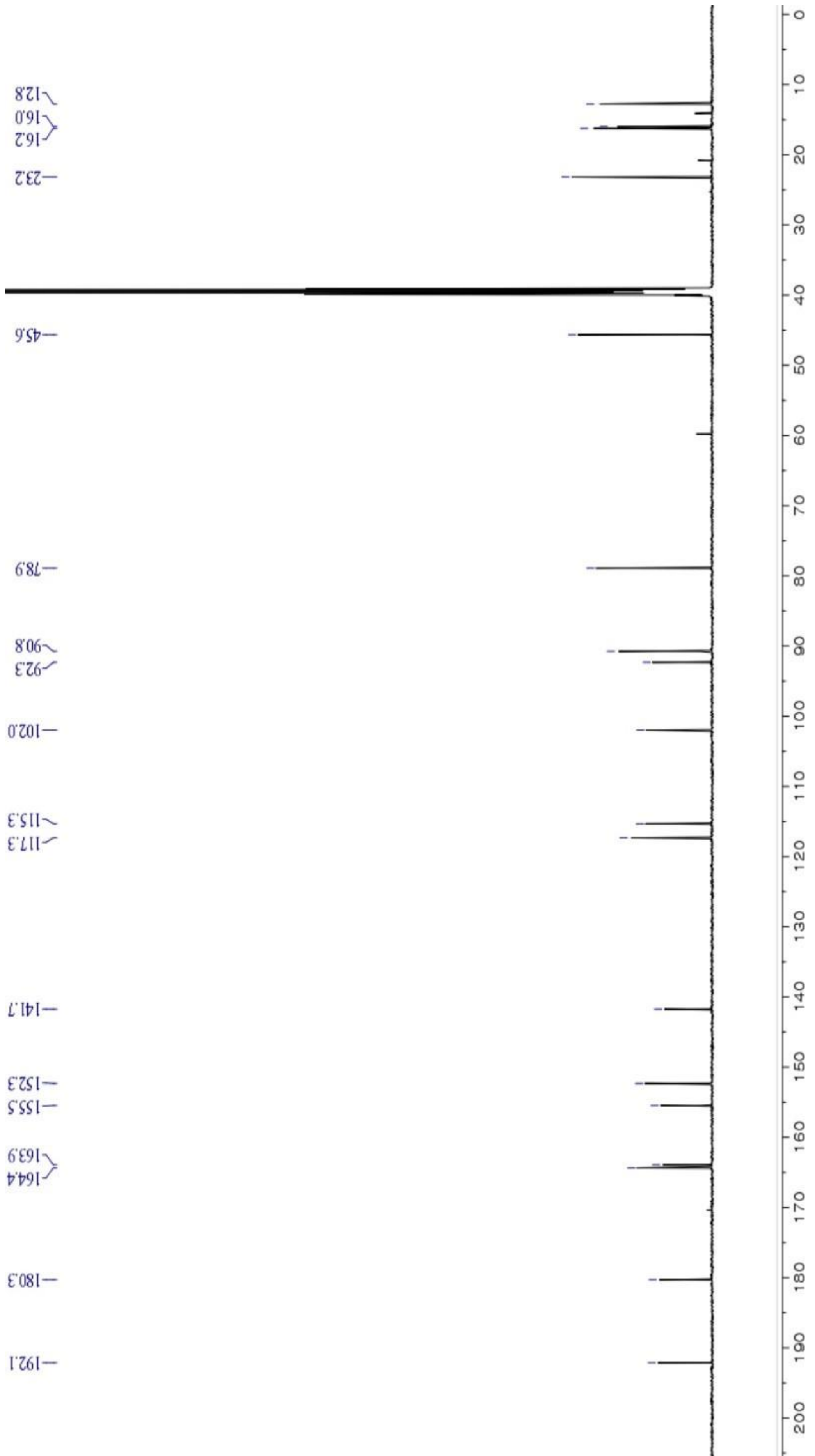

Figure S36. The ^{13}C NMR (150 MHz, DMSO-d_6) spectrum of **6**

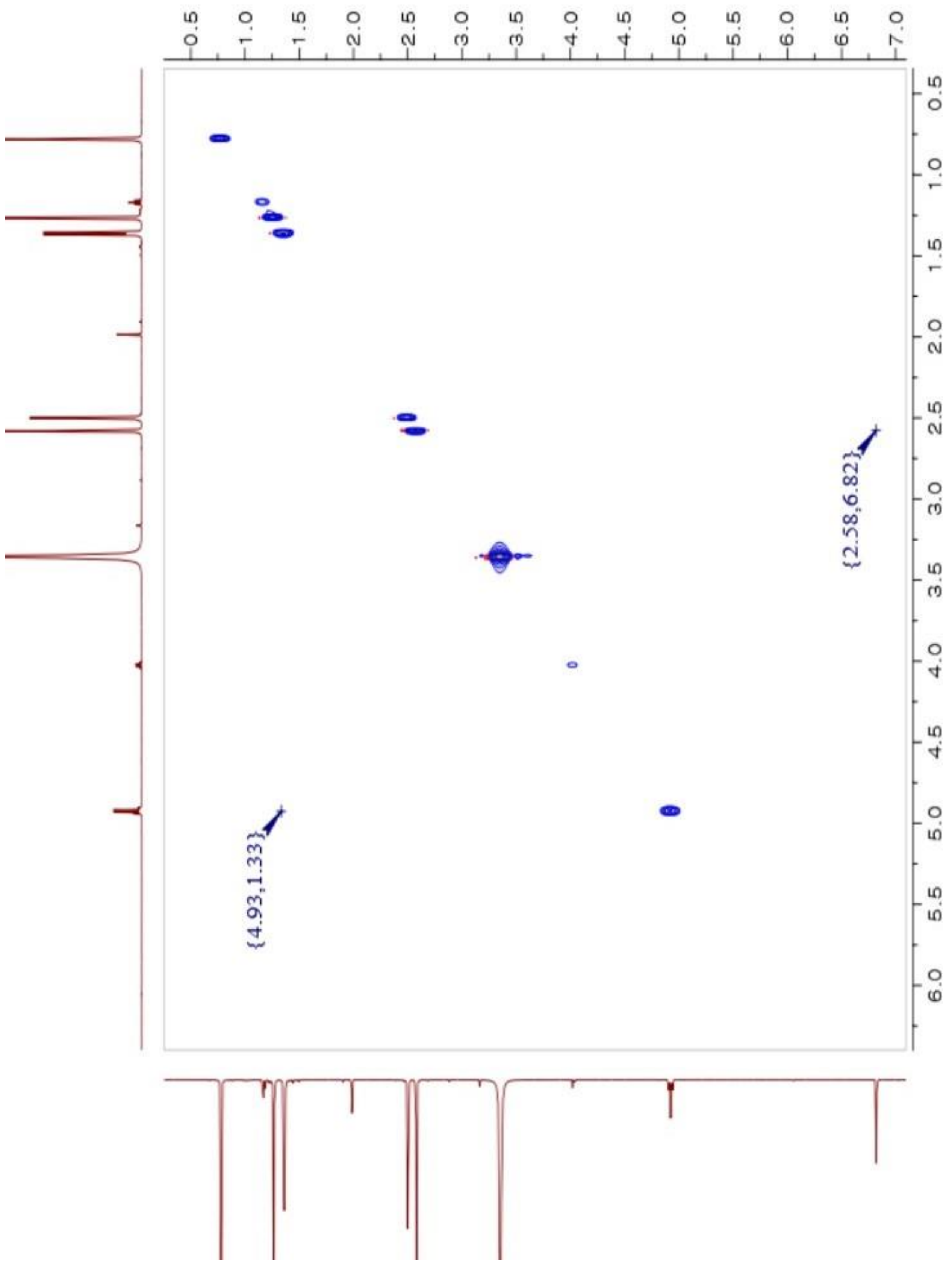

Figure S37. The COSY (600 MHz, DMSO-d_6) spectrum of **6**

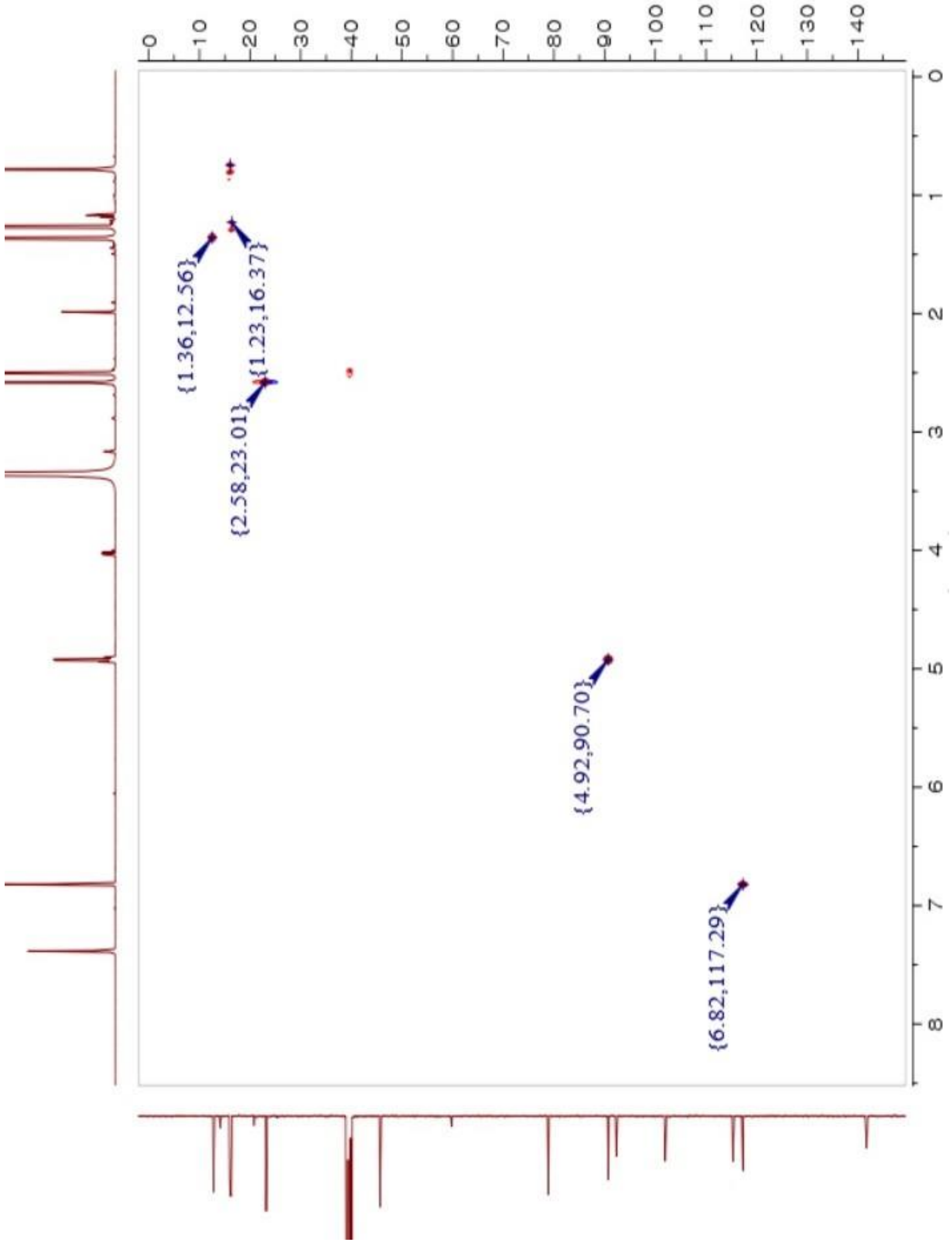

Figure S38. The HSQC (600 MHz, DMSO-d_6) spectrum of **6**

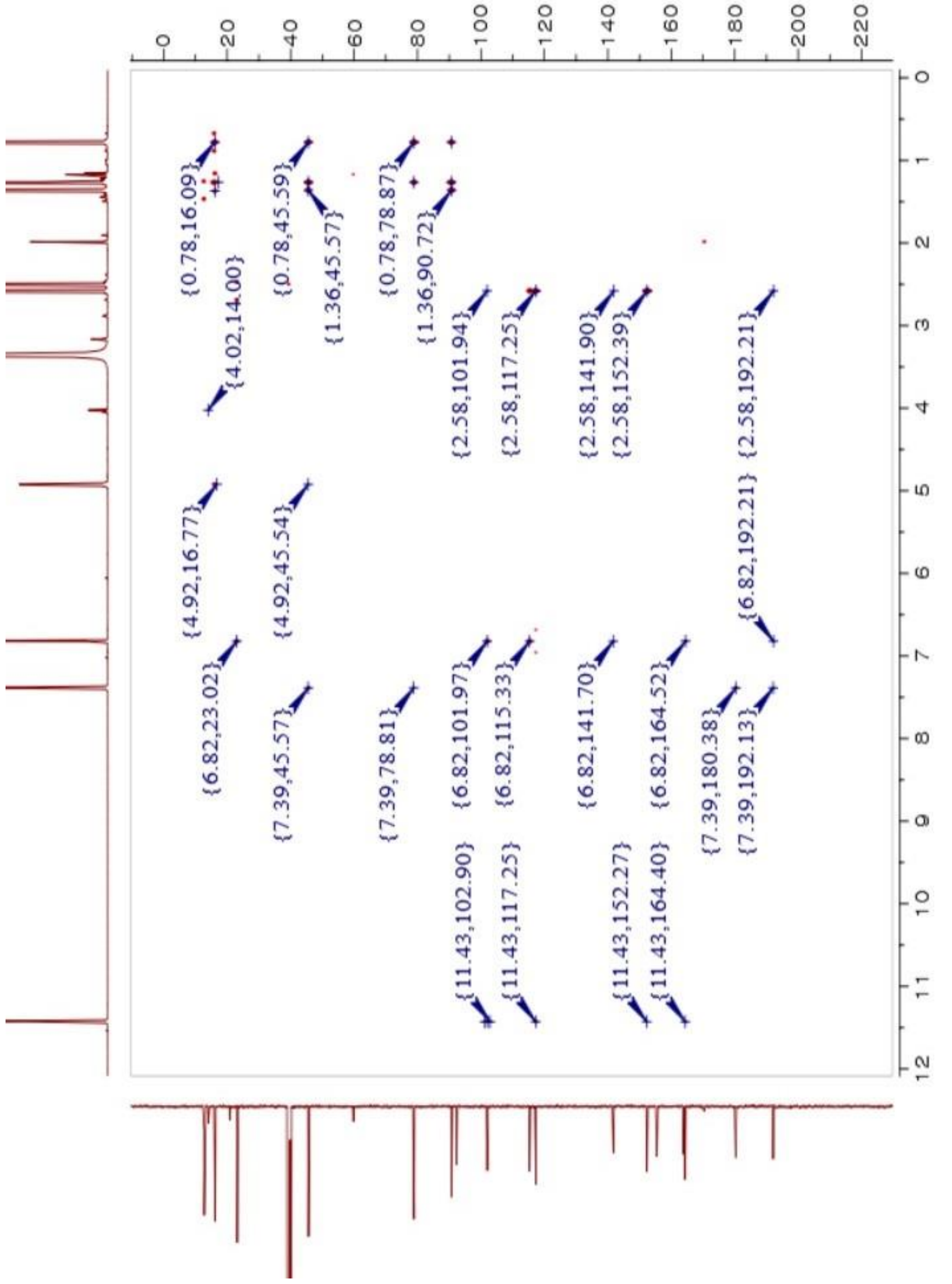

Figure S39. The HMBC (600 MHz, DMSO-d_6) spectrum of **6**

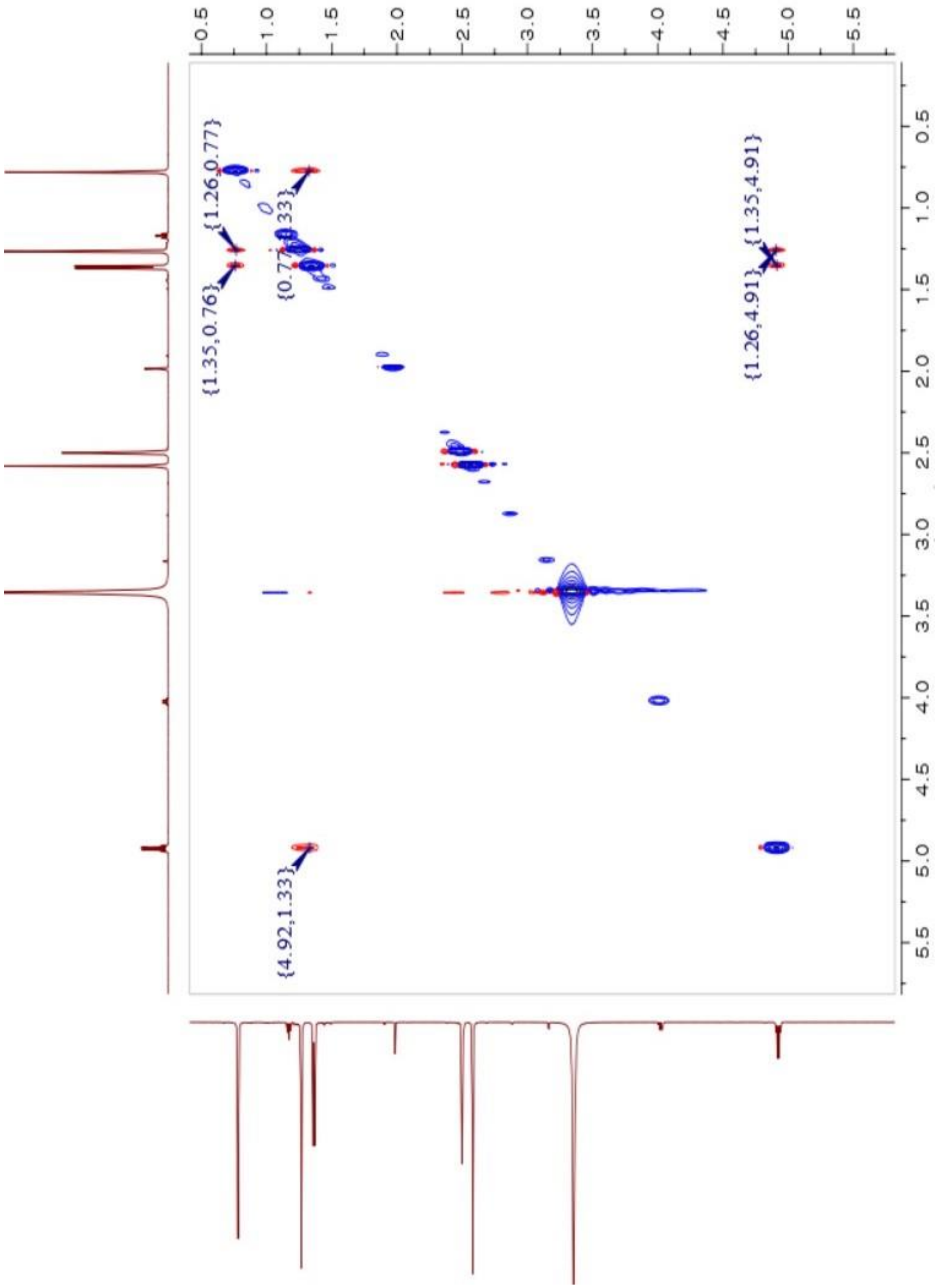

Figure S40. The NOESY (600 MHz, DMSO-d_6) spectrum of **6**

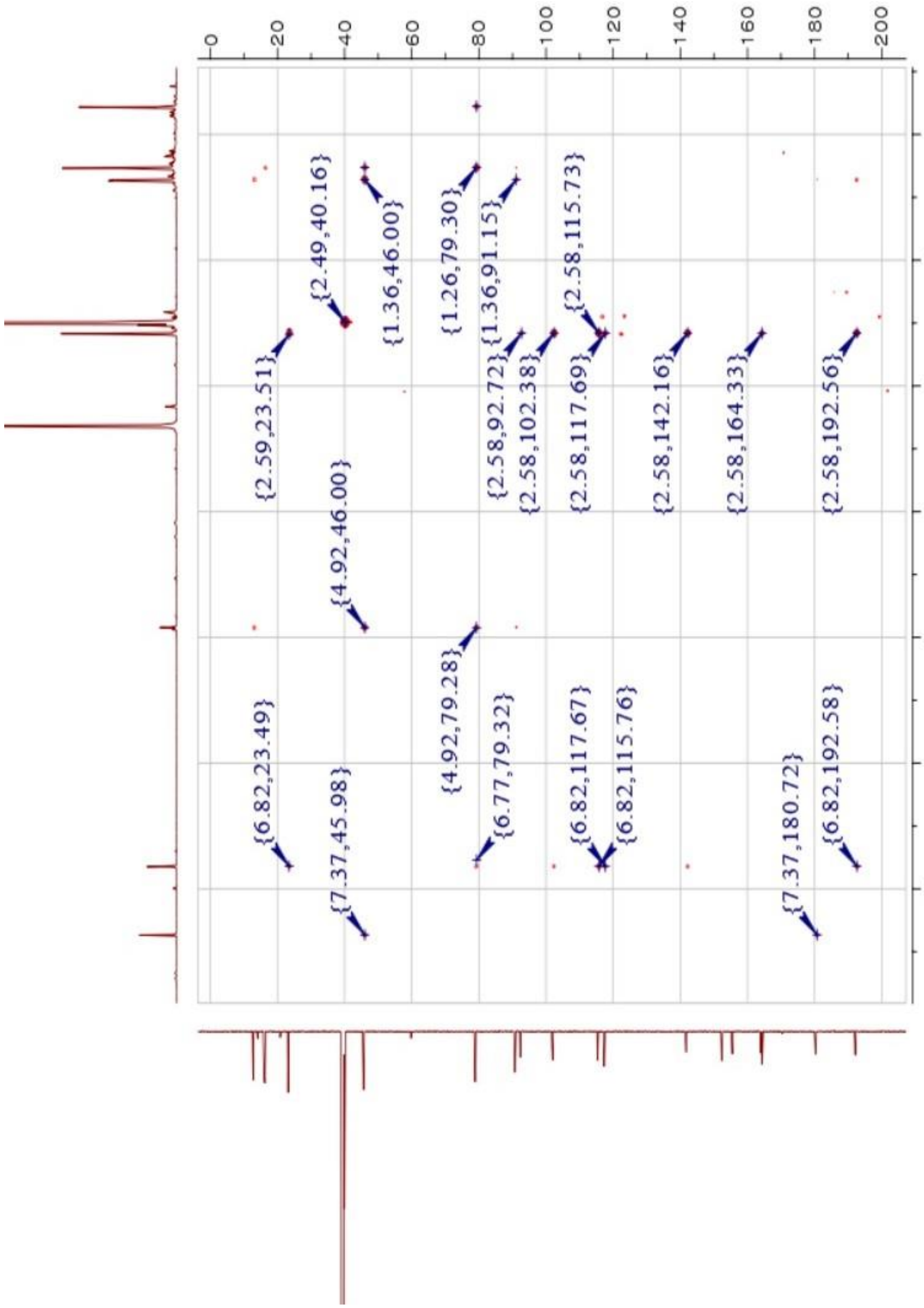

Figure S41. The D-HMBC (800 MHz, J_{CH} = 1 Hz, DMSO-d_6) spectrum of **6**

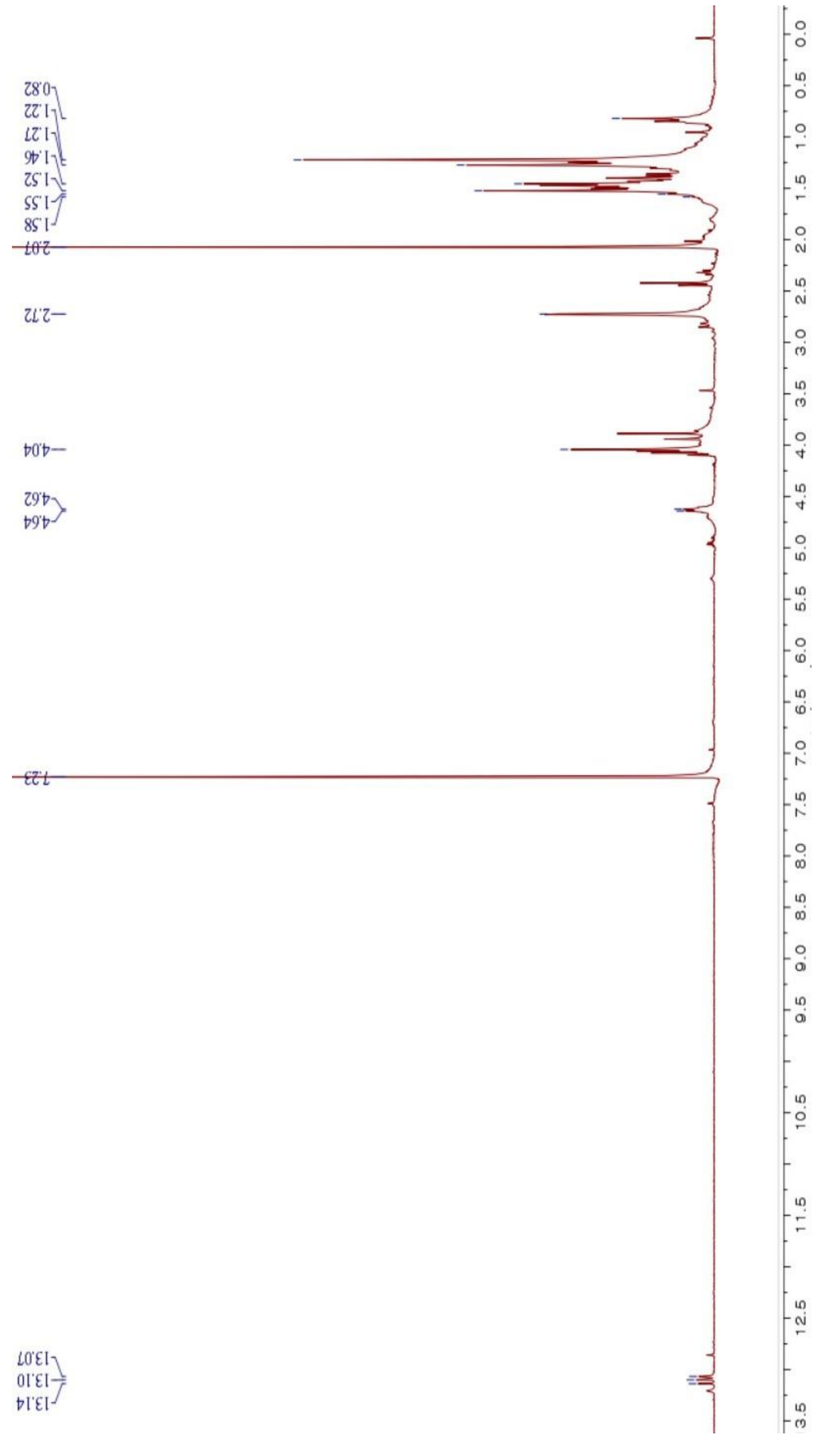

Figure S42. The ^{1}H NMR (400 MHz, $CDCl_3$) spectrum **1a**

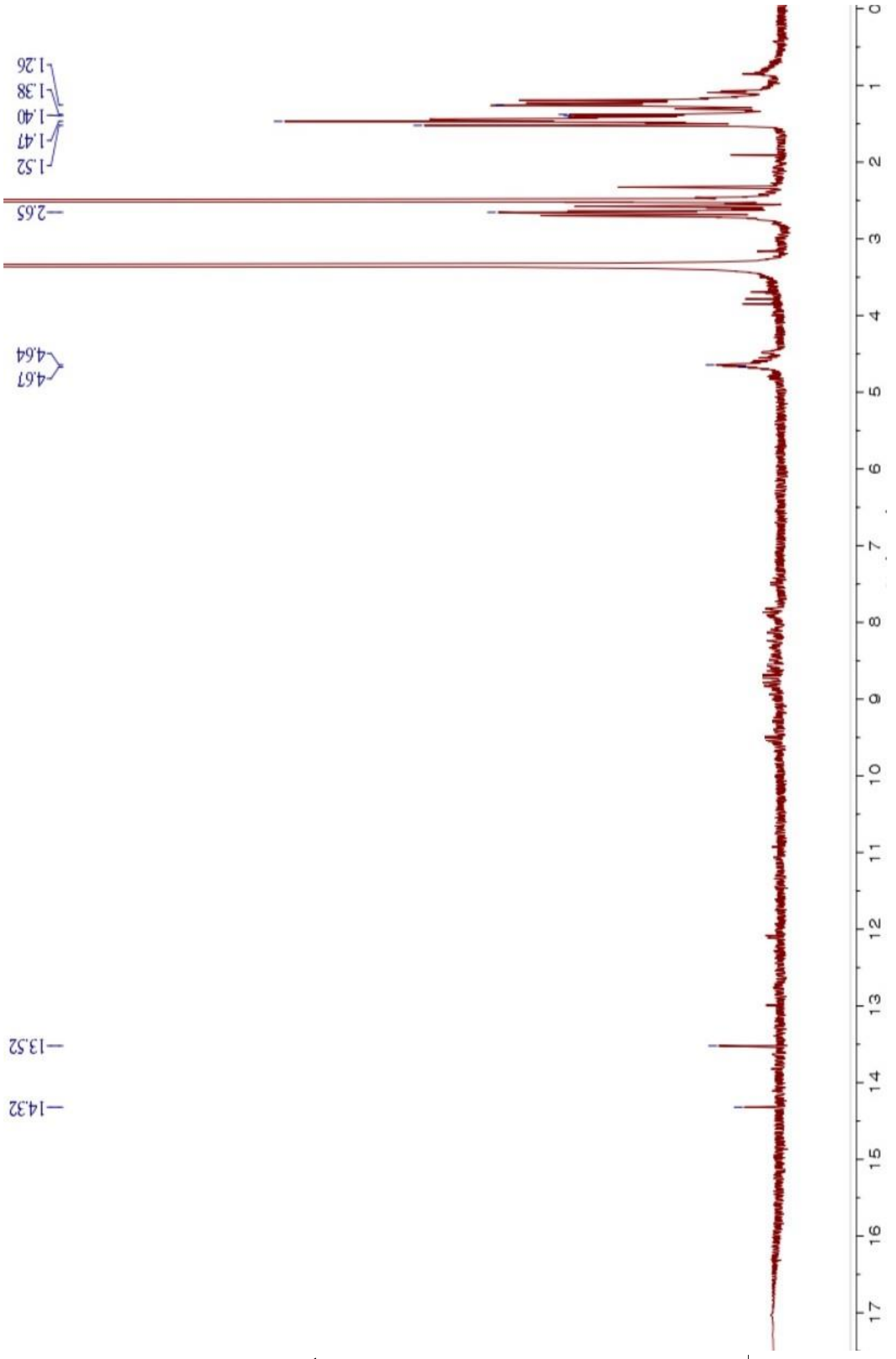

Figure S43. The ^{1}H NMR (400 MHz, DMSO-d_6) spectrum **2a**

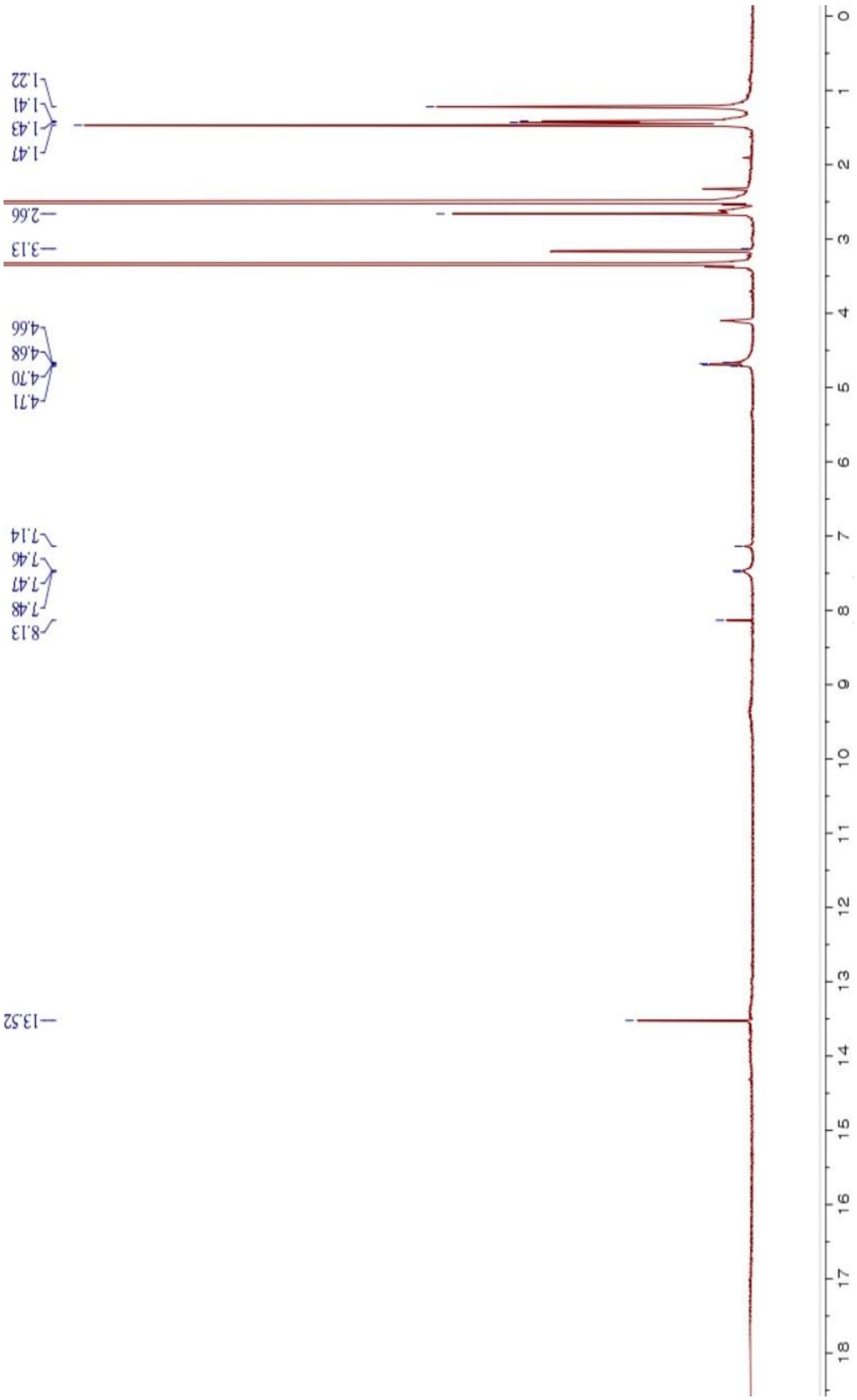

Figure S44. The ^{1}H NMR (400 MHz, DMSO-d_6) spectrum **3a**

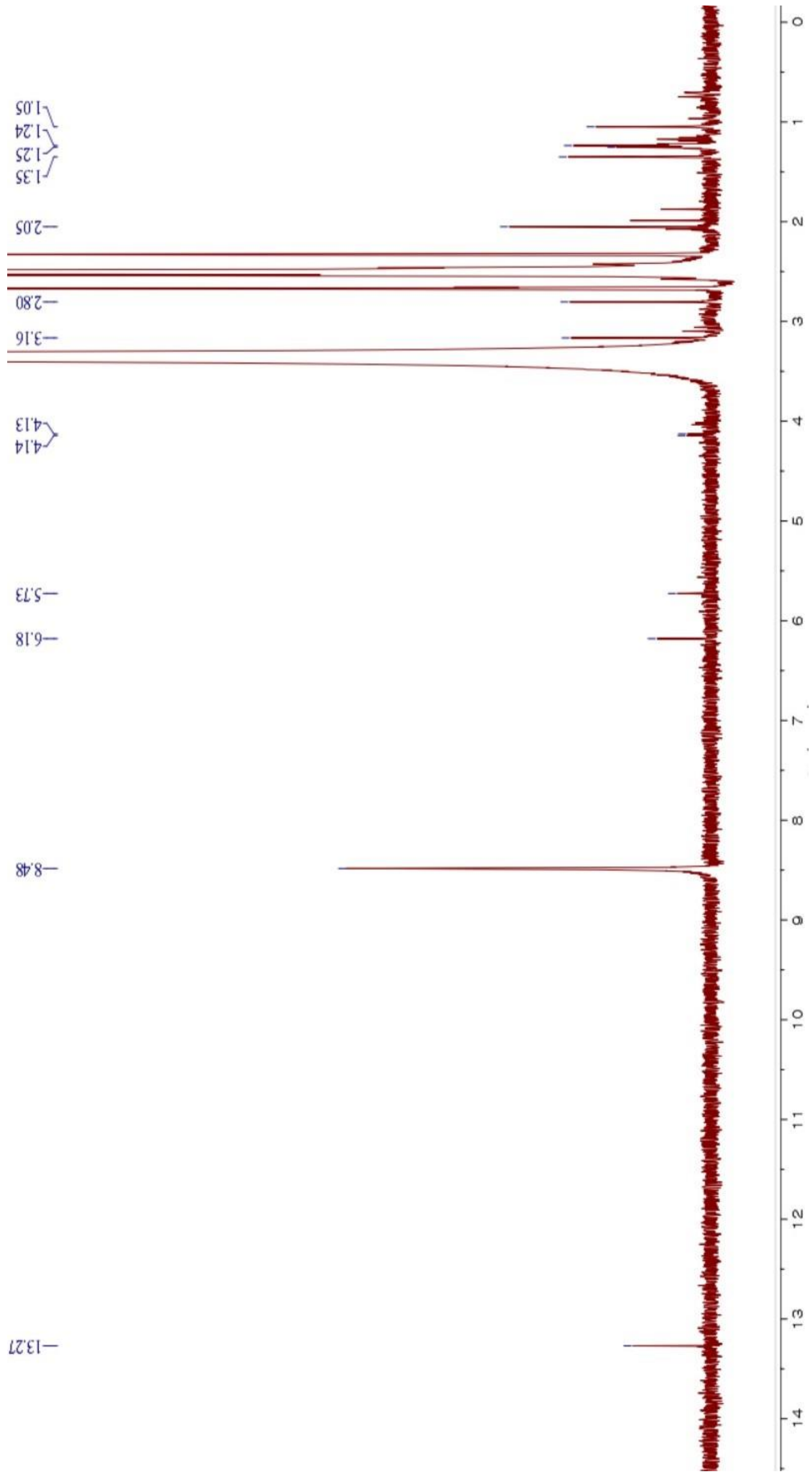

Figure S45. The ^{1}H NMR (400 MHz, DMSO-d_6) spectrum **4a**

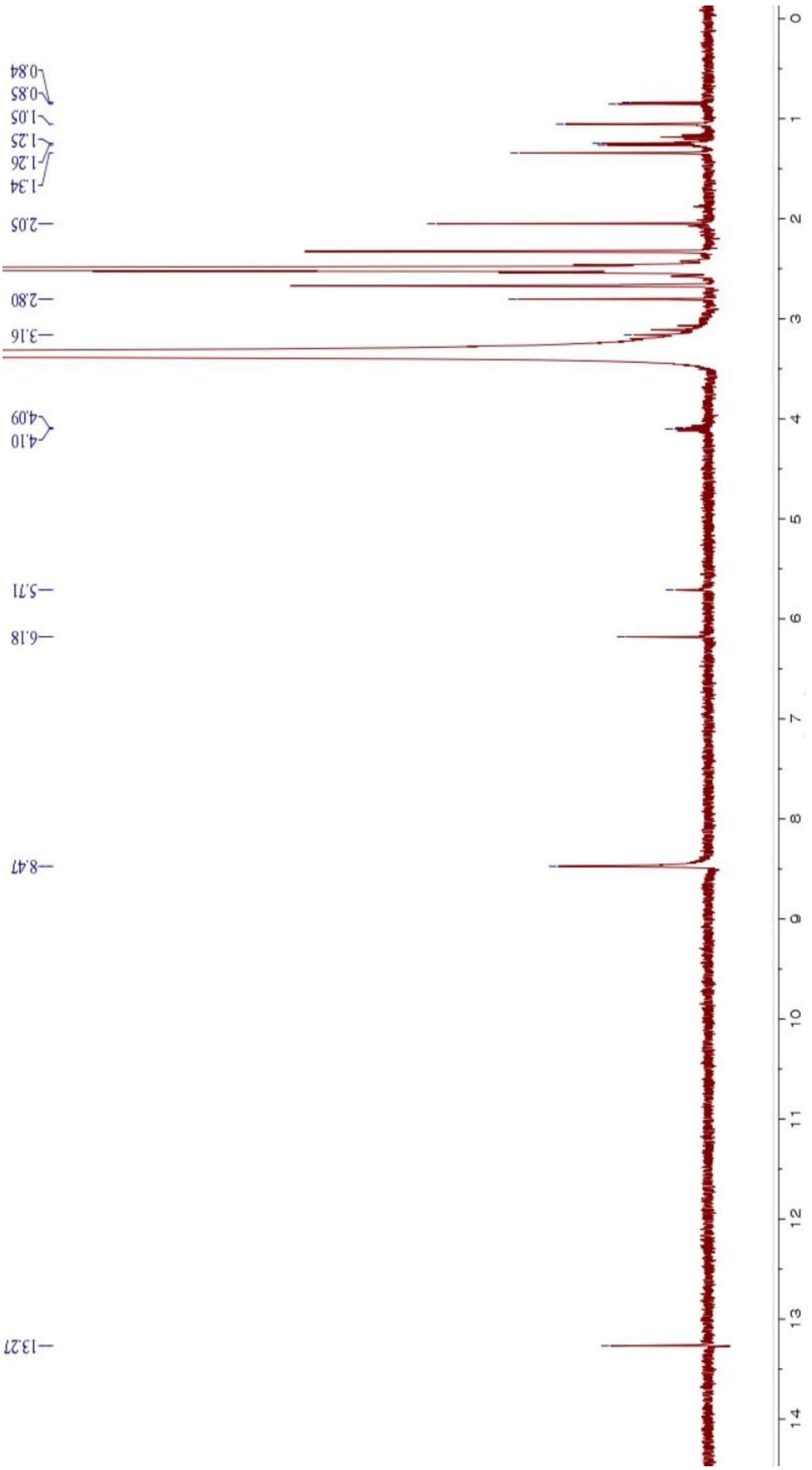

Figure S46. The ^{1}H NMR (400 MHz, DMSO-d_6) spectrum **5a**

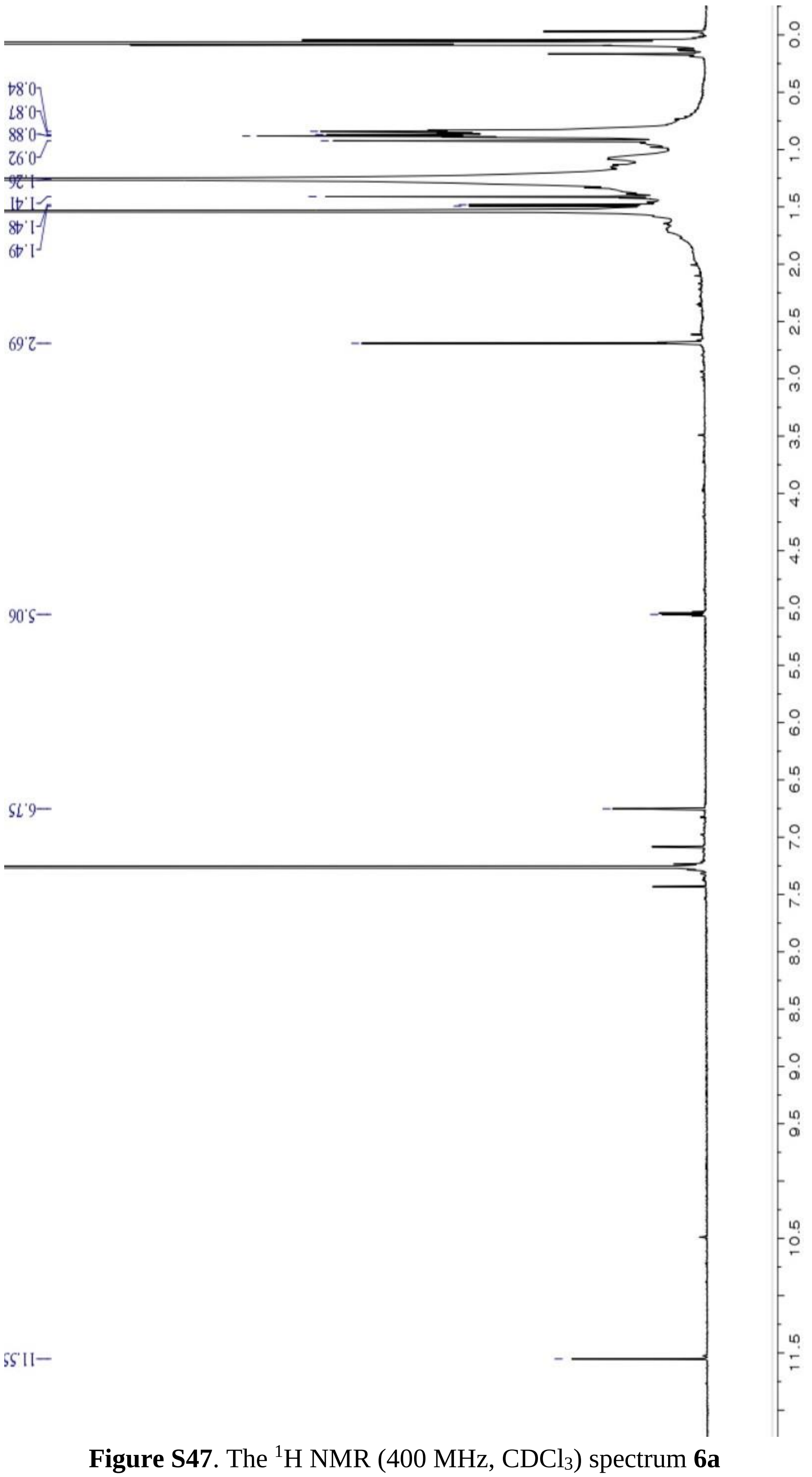

Figure S47. The ^{1}H NMR (400 MHz, $CDCl_3$) spectrum **6a**

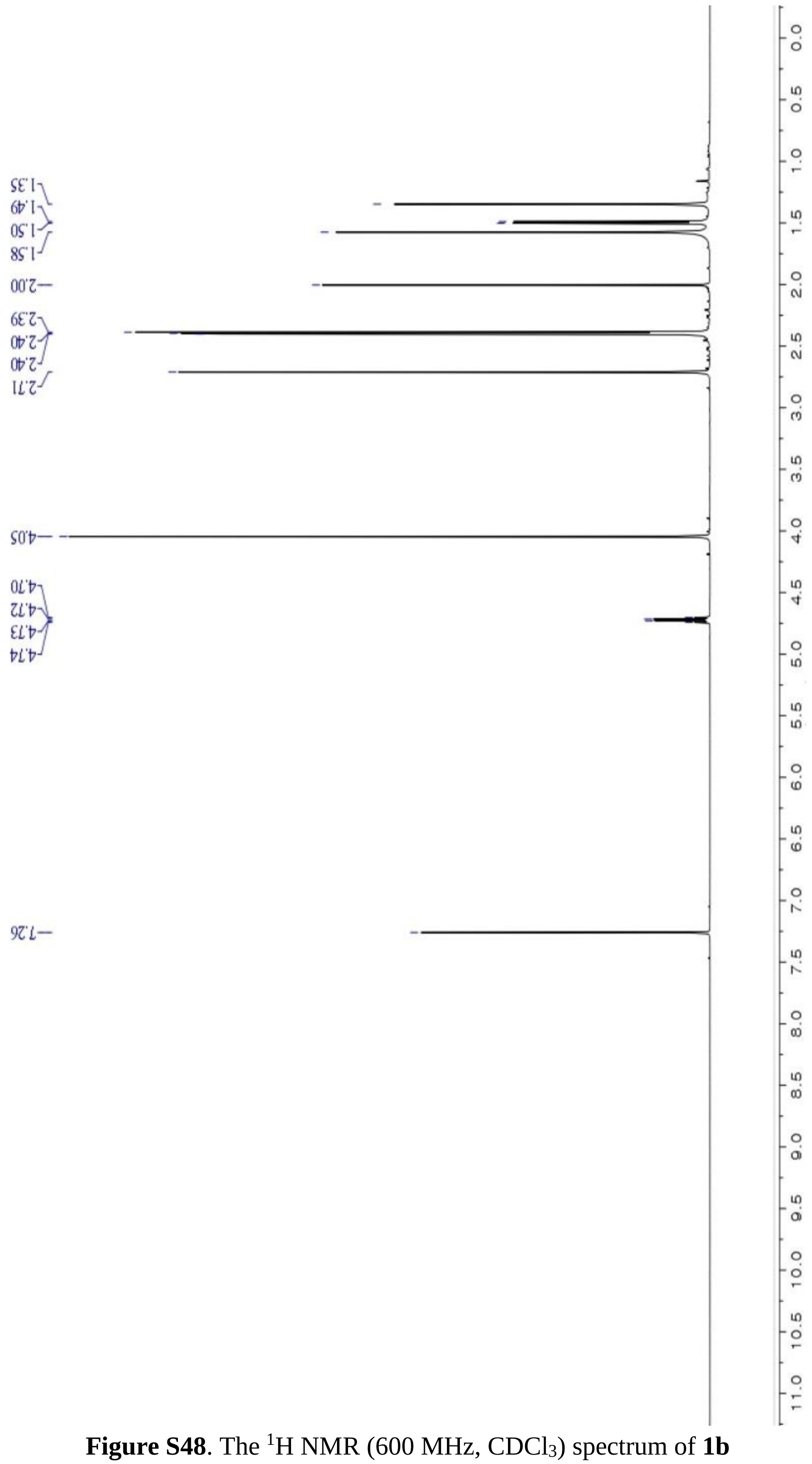

Figure S48. The ^1H NMR (600 MHz, $CDCl_3$) spectrum of **1b**

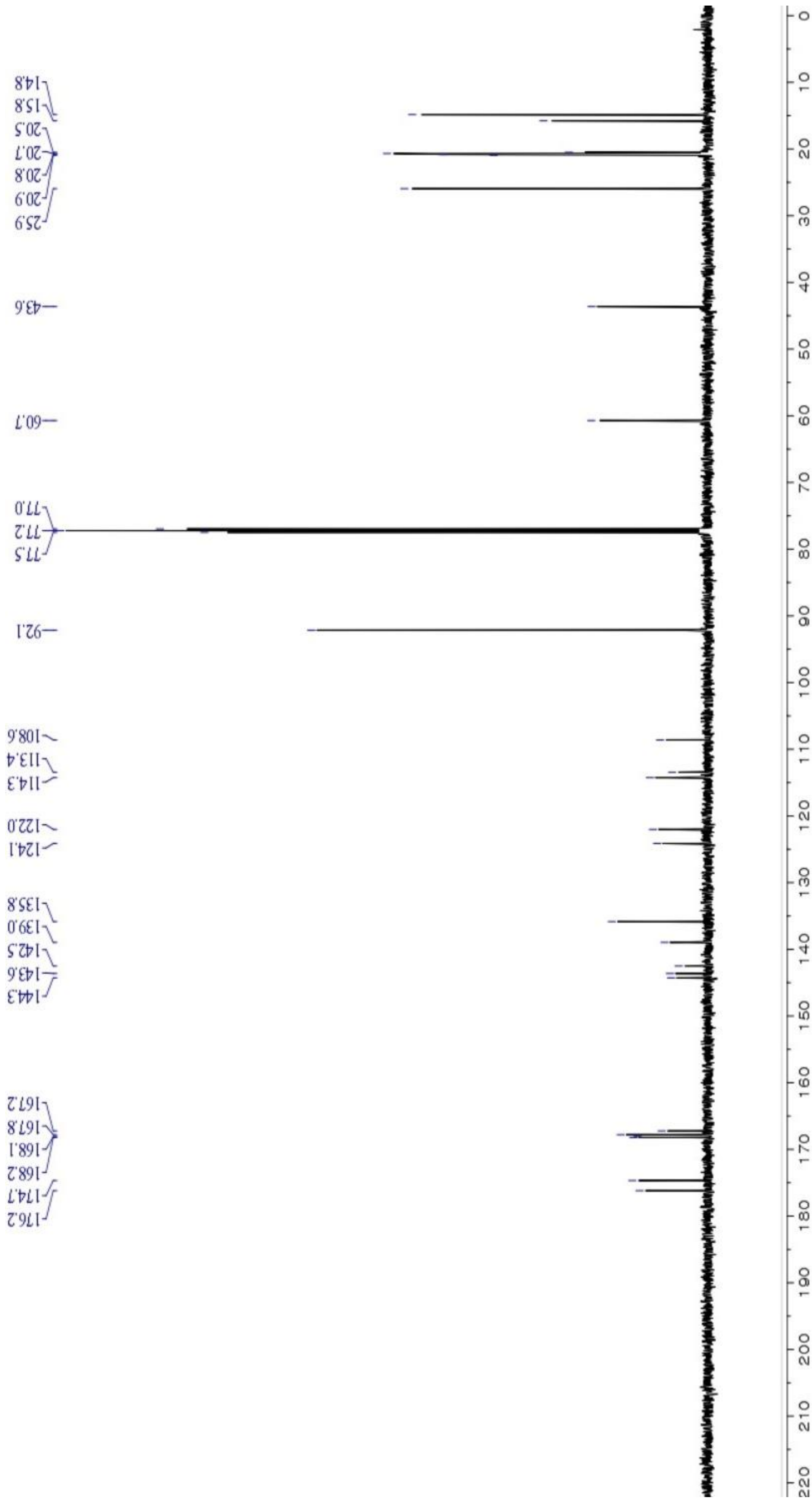

Figure S49. The ^{13}C NMR (150 MHz, $CDCl_3$) spectrum of **1b**

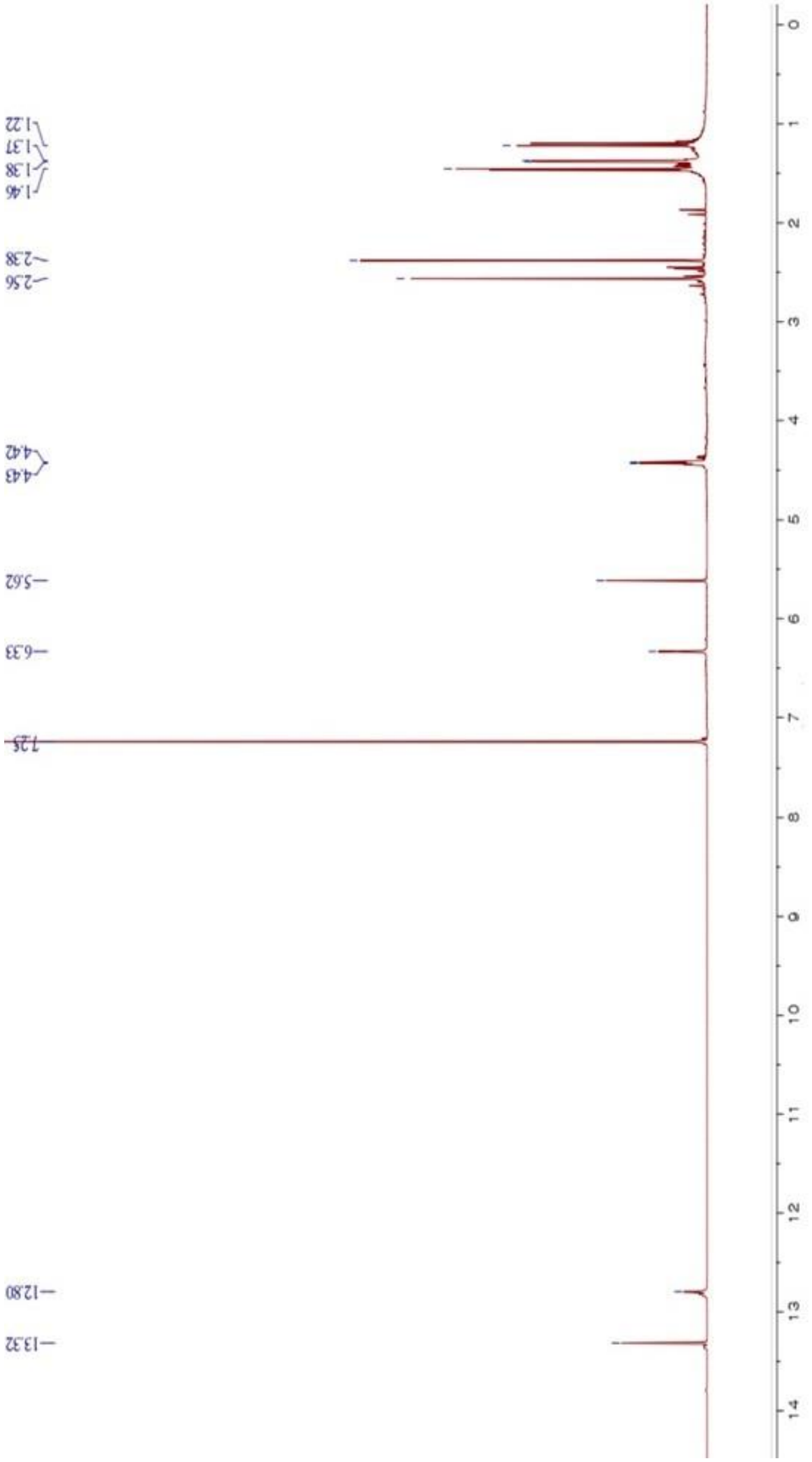

Figure S50. The ^{1}H NMR (400 MHz, $CDCl_3$) spectrum of **4b** (**5b**, **7a**)

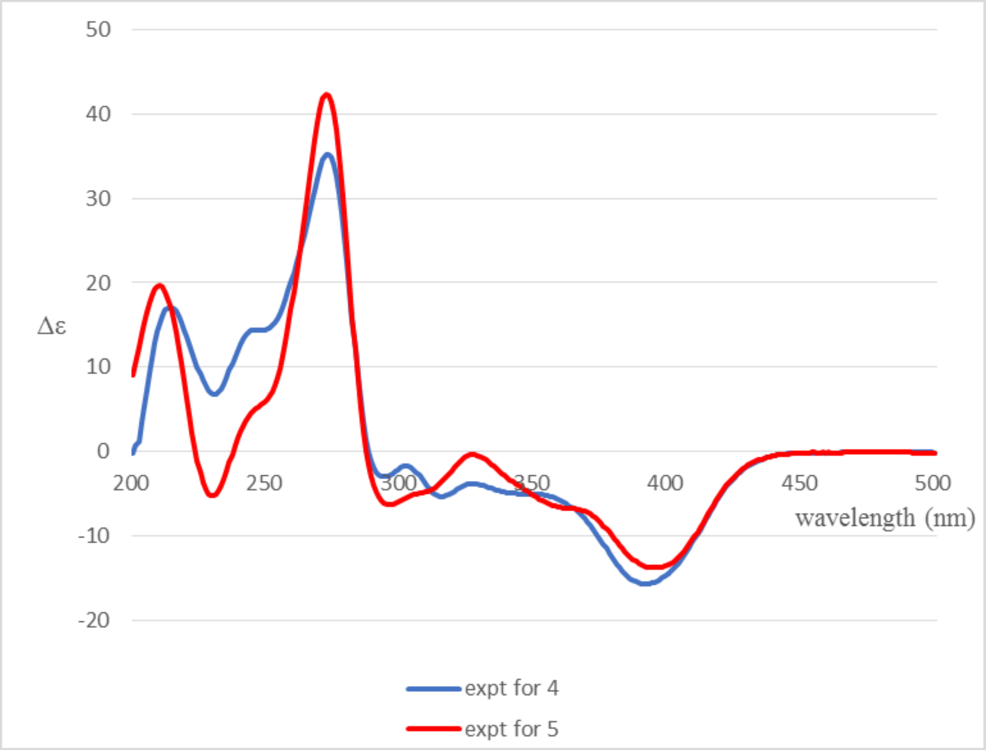

Figure S51. The CD spectra of **4** and **5**

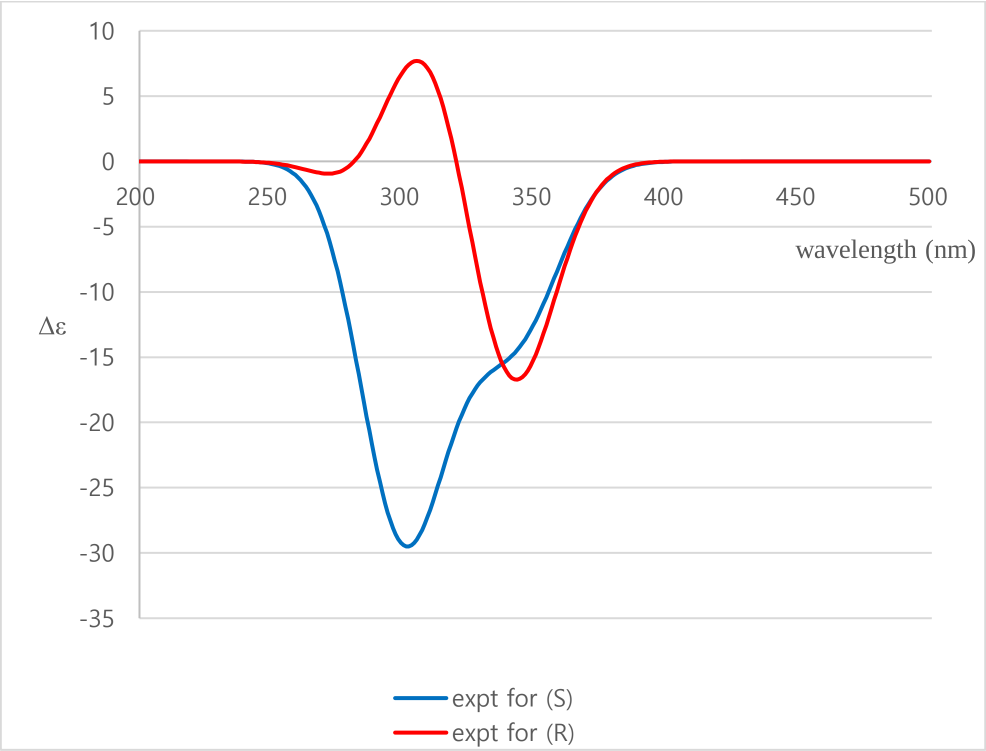

Figure S52. The ECD spectra of **4** and **5**

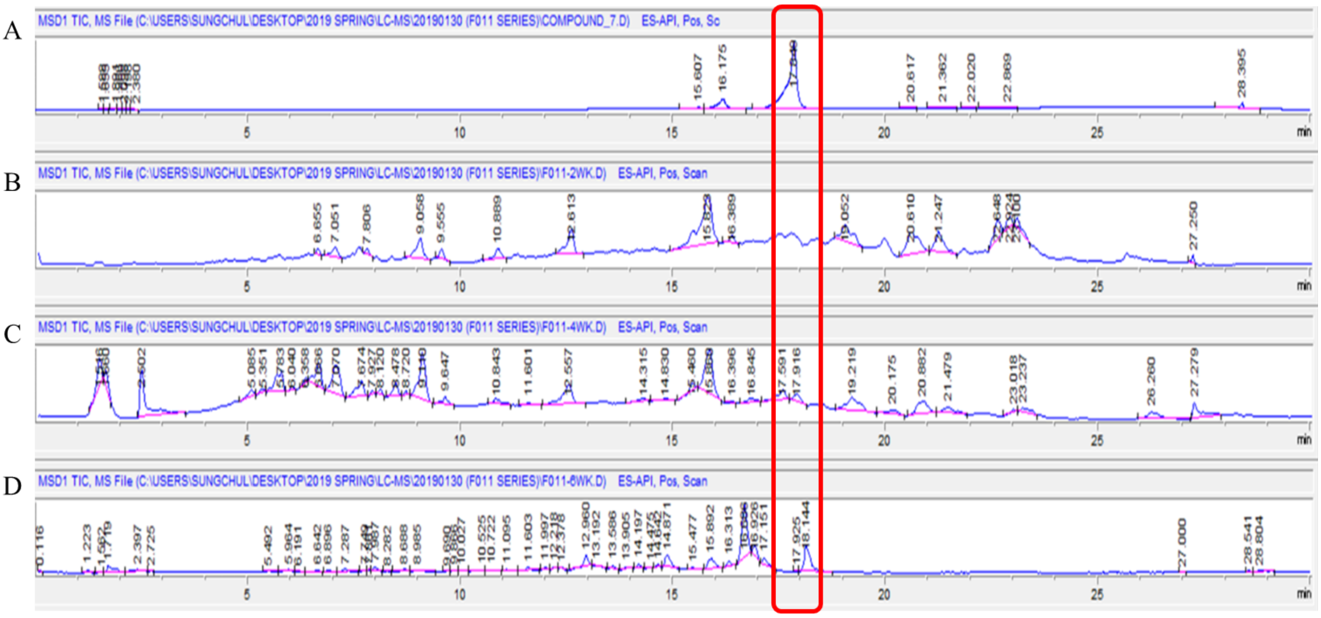

Figure S53. The time-scale LC-MS analysis of **7** (A) Compound **7** (B) Extract from 2 weeks of incubation (C) Extract from 4 weeks of incubation (D) Extract from 6 weeks of incubation

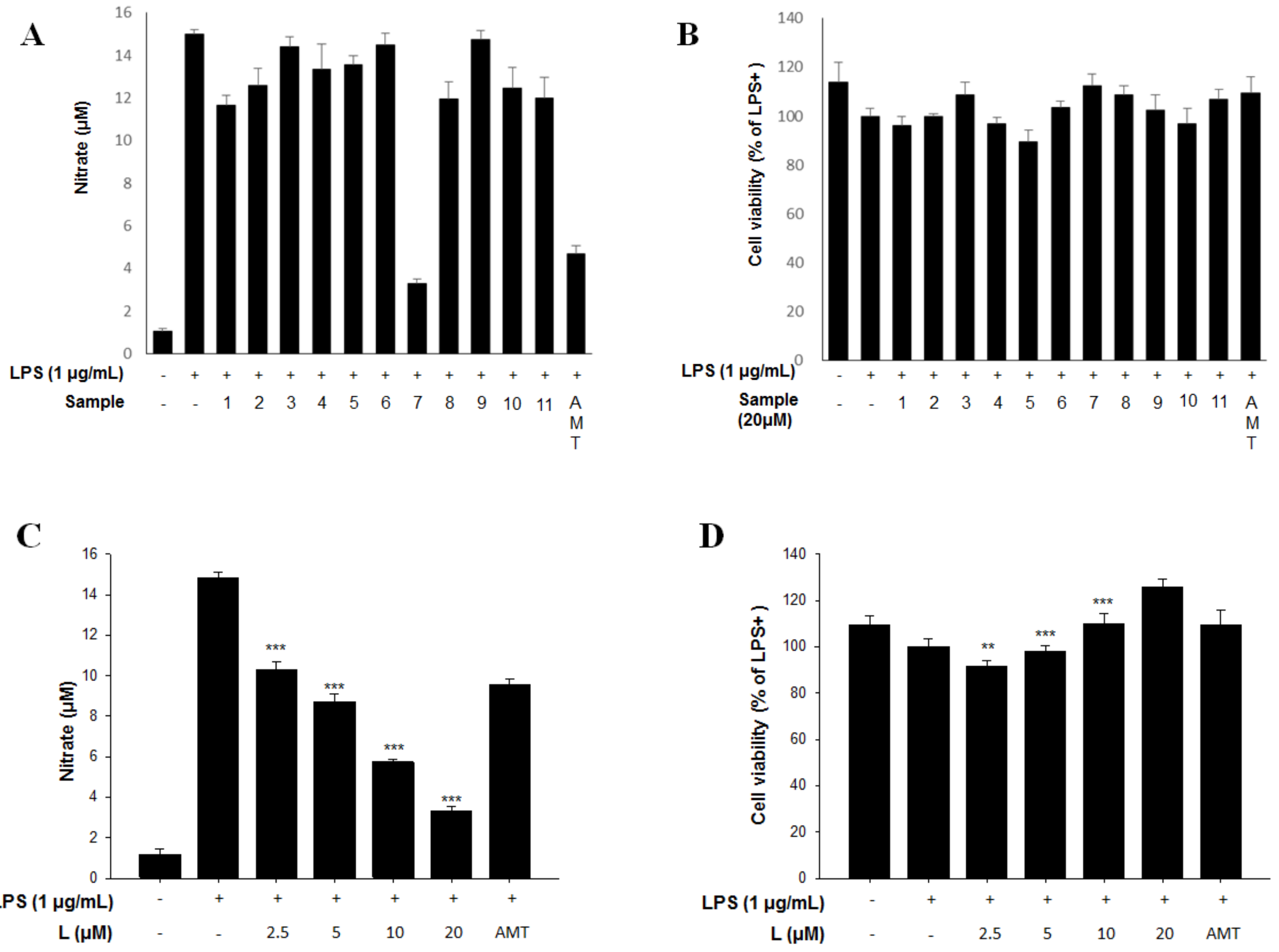

Figure S54. Inhibition of NO production of herqueinone compounds purified from *Penicillium herquei*. The effects of compounds on NO production in RAW 264.7 cells. (A) Cells were pre-treated with compounds (20 uM) and AMT (0.2 uM) for 1 h and LPS (1 ug/mL) was added further incubated for 18 h. The amount of iNOS was examined by Griess reaction. (B) After iNOS level was measured, cell viability was tested using MTT assay. The values are expressed as the means ± SD of triplicate tests. (C) RAW 264.7 cells were pretreated with various concentrations of compound **7** and AMT (0.2 uM) for 1 h, and LPS (1 ug/mL) was added further incubated for 18 h. The amount of iNOS was examined by Griess reaction. (D) Cell viability was tested by MTT assay. The values are expressed as the means ± SD of triplicate tests.

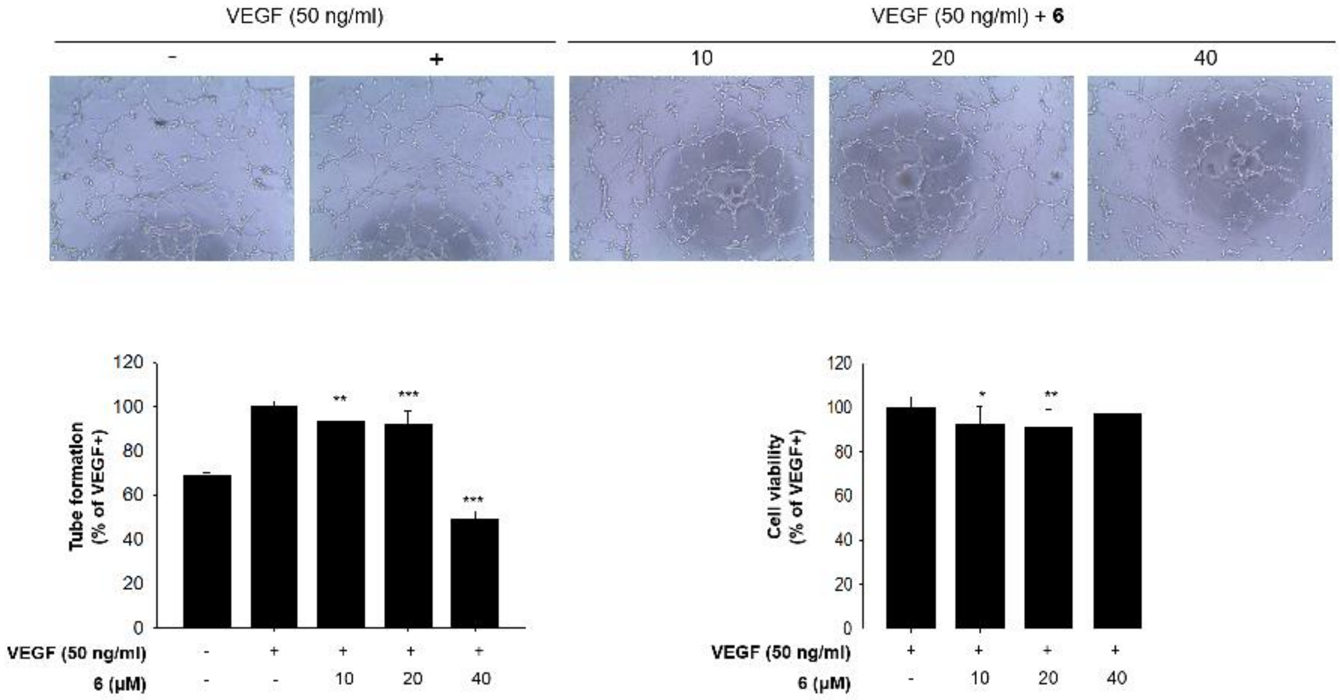

Figure S55. Angiogenesis activities of herqueinone compounds purified from *Penicillium herquei*. Effects of **6** on VEGF-induced tube formation and cell viability. Tube formation activity was measured after cells were incubated with indicated concentrations of **6** on matrigel-coated 96wells for 6 h in the presence of VEGF (50 ng/mL) compared to control in the absence of VEGF. Cell viability was measured by MTT assay after treat with indicated concentrations of **6** in the presence of VEGF (50 ng/mL) for 24 h. The values are expressed as the means ± SD of triplicate tests. * $p \leq 0.05$, ** $p \leq 0.01$ and *** $p \leq 0.001$.

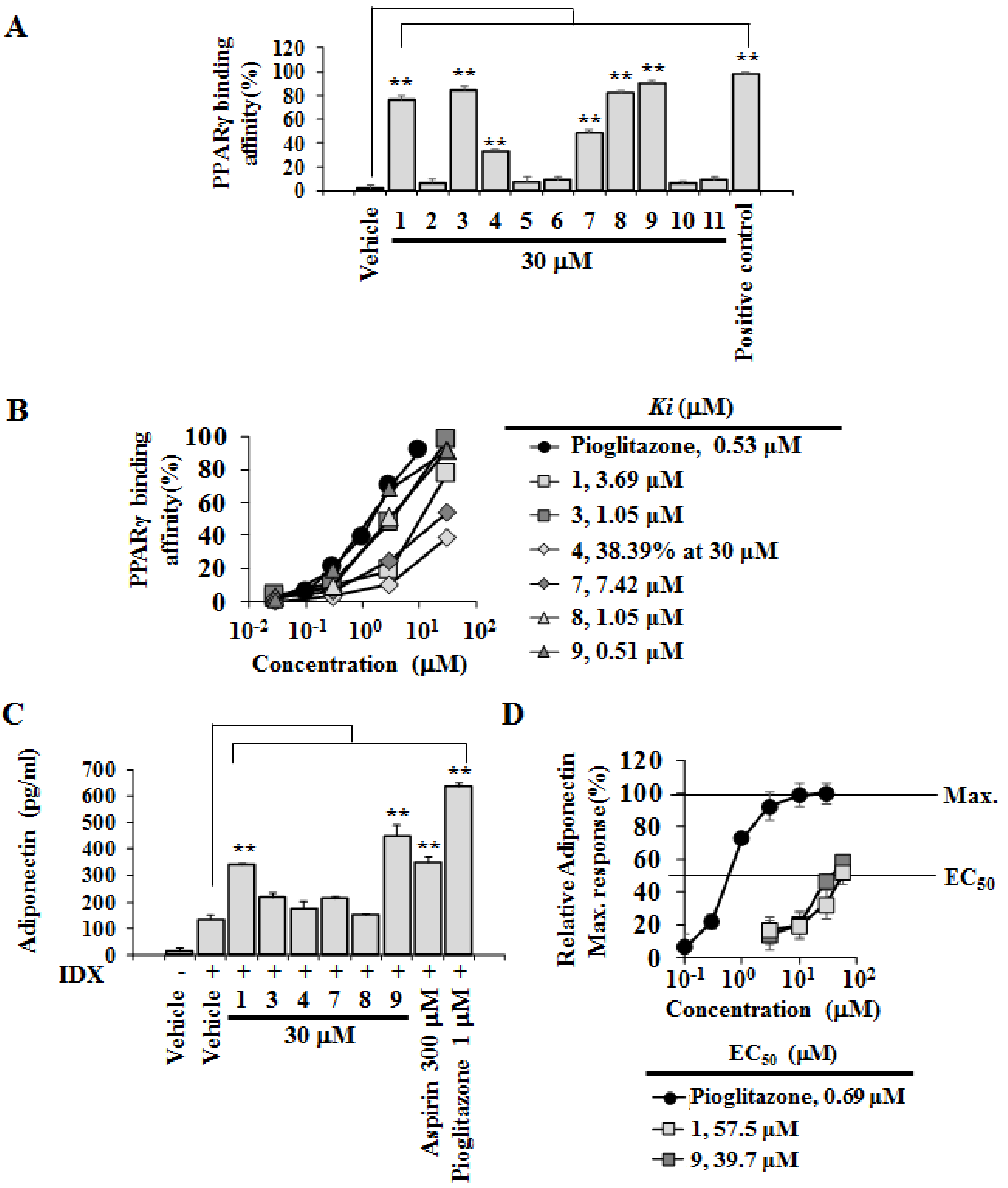

Figure S56. PPARγ binding and adiponectin secretion-promoting activities of herqueinone compounds purified from *Penicillium herquei*. (A) TR-FRET based competitive PPARγ binding assays were performed for herqueinone compounds **1-11**. (B) Ki values for compounds **1**, **3**, **4**, **7**, **8** and **9** were calculated based on the Cheng and Prusoff analysis. (C) Herqueinone compounds were added to the IDX condition when adipogenesis was induced in hBM-MSCs. On the fifth day, the cell culture supernatants were harvested. ELISA was performed to measure the level of adiponectin. Positive controls were pioglitazone and aspirin. (D) The concentration dependency of herqueinone compounds **1**, and **9** on adiponectin secretion-promoting activity was determined. Values represent means ± SD (n=3); * $p \leq 0.05$ and ** $p \leq 0.01$.

Table S1. Results of bioactivity tests

	IC50 (μM)		EC50 (μM)
Compound	RAW 264.7	HUVECs	hBM-MSC(B7)
**1**	>20	>40	57.5
**2**	>20	>40	ND
**3**	>20	>40	ND
**4**	>20	>40	ND
**5**	>20	>40	ND
**6**	>20	20.9	ND
**7**	3.2	>40	ND
**8**	>20	>40	ND
**9**	>20	>40	39.7
**10**	>20	>40	ND
**11**	>20	>40	ND
AMT	0.2		
Sunitinib		1.5	
Pioglitazone			0.69
